# Synthesis and characterisation of rylene diimide dimers using molecular handcuffs†
†Electronic supplementary information (ESI) available: Synthetic and characterisation methods are described in detail; ^1^H and ^13^C NMR spectra; further electrochemical, spectroelectrochemical, EPR, near- and mid-IR spectroscopy data; details of molecular dynamics (MD) studies, including MD movies, and DFT calculations. See DOI: 10.1039/c9sc00167k


**DOI:** 10.1039/c9sc00167k

**Published:** 2019-02-15

**Authors:** Lixu Yang, Philipp Langer, E. Stephen Davies, Matteo Baldoni, Katherine Wickham, Nicholas A. Besley, Elena Besley, Neil R. Champness

**Affiliations:** a School of Chemistry , University of Nottingham , University Park , Nottingham NG7 2RD , UK . Email: Neil.Champness@nottingham.ac.uk

## Abstract

A strategy for positioning, and loosely connecting, molecules in close proximity using mechanically interlocked handcuffs is described. The strategy is demonstrated using rylene diimides, creating dimeric structures in which two components are linked through pillar[5]arene/imidazolium rotaxanes. Investigation of the resulting molecules demonstrates intriguing and new properties that arise from placing these redox active dye molecules together, allowing interactions, whilst allowing the molecules to separate as required. In particular we observe excimer emission from a perylene diimide dimer handcuff and the formation of an unusual radical anion π-dimer upon double reduction of the same molecule. The latter exhibits a unique visible absorption profile for a PDI-based molecule. We demonstrate the flexibility of our approach by making an unprecedented mixed perylene diimide/naphthalene diimide dimer which also reveals interactions between the two components. Our synthetic strategy facilitates the creation of unusual dimeric structures and allows the investigation of intermolecular interactions and the effects they have on electronic and magnetic properties.

## Abstract 2

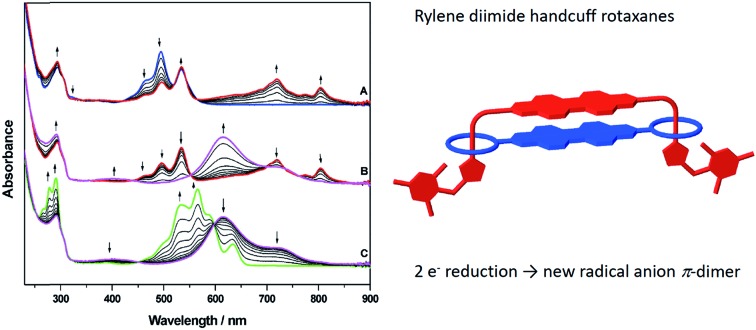
Mechanically interlocked handcuffs provide a strategy to study rylene diimide dimers and to investigate their electronic and magnetic properties.

## Introduction

The ability to organise molecules into predefined configurations lies at the heart of supramolecular chemistry. The use of the mechanical bond,^1^ through the synthesis of interlocked structures such as rotaxanes and catenanes, represents a particularly attractive approach to the organisation of molecular species providing a relatively simple route to the synthesis of highly complex structures that would be challenging to prepare through covalent strategies.^2^,^3^ The stacking interaction between aromatic molecules is fundamentally important^4^ in determining the properties of biological systems, including DNA,^5^ through to organic materials^6^ with electronic and photophysical properties. Indeed, the interaction between adjacent aromatic molecules as they undergo changes in redox state can underpin the conductivity of stacked systems. Herein, we report the use of handcuff rotaxanes^7^–^11^ as a scaffold for the organisation of dimers of rylene diimide dyes, namely perylene (PDIs) and naphthalene diimides (NDIs). We describe a potentially widely applicable strategy for arranging aromatic molecules such that the molecules are both organised in a co-facial manner and yet are free to separate with change of redox state. We believe that our system more closely mimics freely stacked molecules, in comparison to more rigid covalently-bound systems or other interlocked structures such as catenanes,^12^,^13^ and, due to the modularity of our approach, our strategy allows the organisation of different aromatic species that typically would be convoluted to combine in a stacked arrangement.

Rylene diimides are highly important dye molecules that have been extensively studied due to their potential use across a wide variety of applications.^14^ One direction of research that has received attention is the synthesis and study of dimers of such dyes, including PDIs^14^–^26^ and NDIs.^15^,^27^–^31^ The propensity of these large, flat aromatic dyes to aggregate into larger structures is not only of interest from a fundamental viewpoint but also affects the properties of the resulting systems. Thus, extensive effort has been made to synthesise discrete dimers of PDIs in order to evaluate the processes through which co-facial rylene diimides interact and how such interactions contribute to the modification of their properties. The most widely studied approach to the synthesis of dimers has been through covalent coupling at the imide position which leads to comparatively rigid structures, including macrocyclic structures,^15^–^22^,^28^–^33^ with defined and restricted separations and orientations between the diimide moieties. Such a strategy has mostly been applied to perylene diimides and NDIs and most recently to terrylene diimides.^34^ Supramolecular approaches through the design of hydrogen-bonded systems^23^–^25^ or through metal coordination^26^ have also been reported which, in principle, allow a degree of flexibility in terms of perylene diimide organisation.

This study exploits the topological arrangement induced by handcuff rotaxanes to position two rylene diimides in a co-facial manner whilst not restricting the arrangement of the aromatic molecules through the use of covalent or coordination bonds. In contrast to related supramolecular systems that use hydrogen-bonding interactions, the mechanical bonds of the handcuff rotaxanes allow flexibility and yet do not allow the aromatic species to fully disassociate. Our investigations reveal extensive interactions between the handcuffed molecules which lead to unusual spectroscopic, redox and magnetic properties.

## Results and discussion

**Fig. 1:**
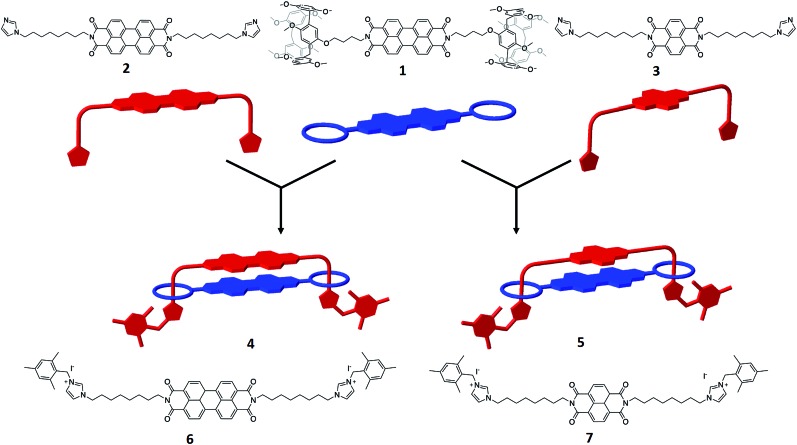
PDI (**1**, **2**) or NDI (**3**), containing components that are used to synthesise handcuff rotaxanes. Note the simple design of each component, either a bis-pillar[5]arene or bis-imidazole appended to an aromatic core. Handcuff formation is readily achieved through the formation of pseudo-rotaxanes between imidazole and pillar[5]arene moieties followed by stoppering through reaction of the imidazole groups and benzyl iodides. Model bis-imidazolium rods, **6** and **7**, are studied for comparison.

The target handcuff rotaxanes exploit the robust interaction between pillar[5]arenes and imidazole and imidazolium groups.^35^–^39^ The interaction between such moieties provides a reliable method for the formation of pseudo-rotaxanes that can be readily locked in place through reaction with appropriate stopper groups.^40^,^41^ In order to form handcuff structures our strategy requires functionalization of the desired aromatic group with imidazole appendages for one component and with pendant pillar[5]arenes for the other component. Reaction of perylene tetracarboxylic dianhydride with an amino-functionalised pillar[5]arene leads to the formation of a bis-pillar[5]arene-functionalised species (**1**) in which the two macrocycles are separated by the PDI aromatic core (Fig. 1). Similarly reaction of perylene tetracarboxylic dianhydride with an imidazole group functionalized with an alkylamine arm leads to the formation of a bis-imidazole species (**2**) in which, and similarly to the pillar[5]arene-functionalised species, the two arms are separated by the PDI aromatic core (Fig. 1). The simplicity of this approach allows, in principle, modification of the core of either of the handcuff components and we demonstrate this through the synthesis of a bis-imidazole functionalized NDI (**3**).

**Fig. 2:**
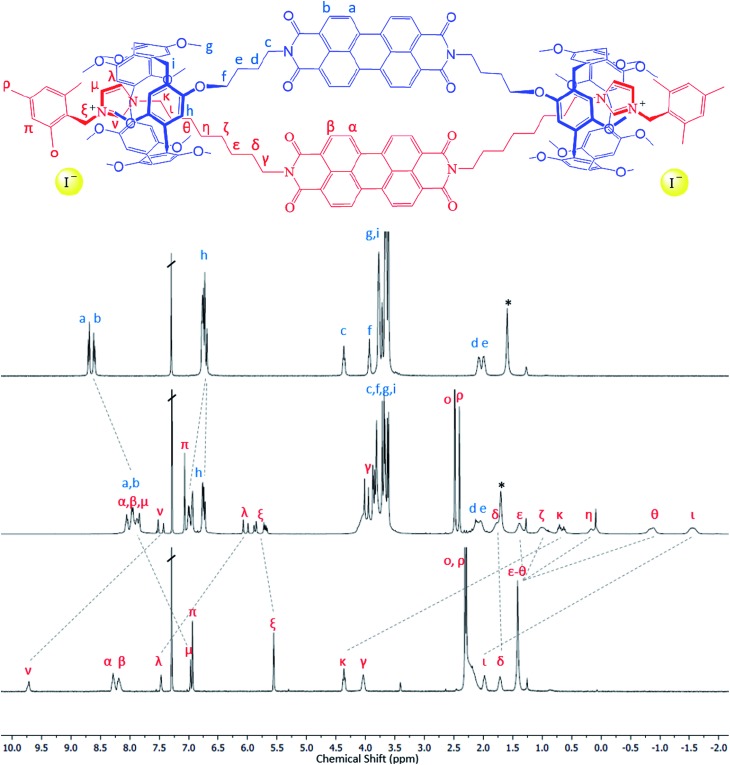
^1^H NMR spectra illustrating formation of the handcuff structure, **4**. Top: bis-pillararene component, **1**; middle: handcuff **4**; bottom – non-interlocked bis-imidazolium rod, **6**. See Fig. S25† for ^1^H NMR spectra of handcuff **5**.

Reaction of the bis-pillar[5]arene, **1**, with either bis-imidazole functionalized species, **2** or **3**, gives rise to pseudo-rotaxanes in which each imidazole group sits within a pillar[5]arene host. Subsequent reaction with a bulky aryl iodide, 2-(iodomethyl)-1,3,5-trimethylbenzene, at –15 °C leads to alkylation of the available imidazole nitrogen and stoppering of the rotaxane to form a handcuff structure in high yield (**4**, 81%; **5**, 89%). Whereas combination of **1** with the PDI-containing species **2** leads to the formation of a PDI–PDI dimer, **4**, reaction of **1** with the NDI-containing rod **3** affords an NDI–PDI dimer, **5**. ^1^H NMR spectroscopy, including NOESY spectra, confirms the formation of the handcuff structure through strong upfield shifts of the protons associated with the imidazoles and the alkyl chain linking the imidazole to the PDI/NDI core (*κ*, *η*, *θ*, *ι*) (Fig. 2). Such shifts are characteristic of rotaxanes formed between pillar[5]arenes and alkyl-imidazole groups.^39^ For the purposes of comparison model bis-imidazolium rods were prepared by reaction of either **2** or **3** with 2-(iodomethyl)-1,3,5-trimethylbenzene to give compounds **6** and **7** respectively. It is noteworthy that there is no evidence for the formation of polymeric structures, presumably as a result of favourable inter-rylene interactions and entropic contributions. The stability of the handcuff structures was assessed by VT NMR in CDCl_3_ over the temperature range 263–313 K (**4** and **5**, Fig. S14 and S19† respectively), by heating samples to 393 K in d_6_-DMSO (**4**) and by monitoring UV-visible spectra of **4** in either CHCl_3_ (338 K) or DMSO (368 K) (Fig. S38 and S39†, respectively). In all experiments the handcuffs were found to be stable with no indication of degradation, confirming that the 2-(methylene)-1,3,5-trimethylbenzene stopper was sufficiently large to prohibit dethreading processes over these temperature ranges.^42^

**Fig. 3:**
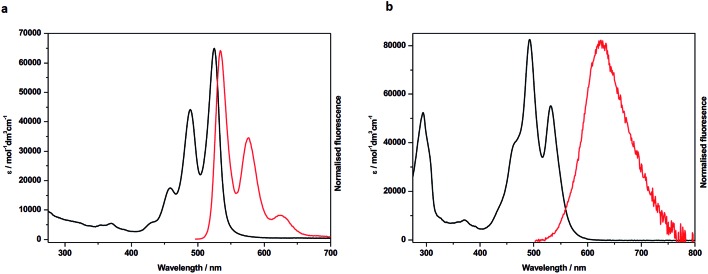
UV/vis absorption (black trace) and emission (red trace) of: (a) **6** and (b) **4** both in CH_2_Cl_2_.

In order to probe the nature of the interactions between the two rylene diimide components of the handcuffs in **4** and **5** each system was studied by UV-visible spectroscopy, fluorescence, cyclic voltammetry (CV) and by spectroelectrochemistry. The UV/vis spectra of the PDI precursors, **1** and **6**, in CH_2_Cl_2_ (Fig. 3a, S26 and Table S1†) show absorption profiles consistent with the presence of monomeric PDIs in solution.^23^ The visible spectrum of **4** in CH_2_Cl_2_ (Fig. 3b), CHCl_3_, MeCN or THF (Fig. S27†) shows an intensity reversal in the 0 → 0 and 0 → 1 transitions to give a spectral profile consistent with a π-stacked chromophore^43^ with PDI cores in a face-to-face arrangement.^44^,^45^ This is in contrast to the UV/vis spectrum of a 1 : 1 mixture of **1** and **6** which is essentially that of the sum of the individual components (Fig. S32†). In contrast to **4**, the PDI-related bands in **5** appear to indicate monomeric character, *i.e.* unstacked (Fig. S28†), as are the bands associated with the NDI component which are not significantly blue shifted relative to those in the NDI precursor **7** (Table S1, Fig. S26†). In a previous report a significant blue shift in these NDI bands has been attributed to exciton coupling in an H-aggregated NDI–NDI dimer.^27^

The emission spectra for **4** also reveal significant differences between the handcuff rotaxane structure and the component PDIs, **1** and **6**. For **1**, in which the PDI is linked to two pillararene groups *via* a butyl linker, the fluorescence in CHCl_3_ is significantly reduced (*Φ* = 0.002) indicating that an efficient non-radiative decay pathway exists between the PDI and pillararene appendages, as observed for related calixarene–PDI systems.^46^–^49^ A solution of **6** in CH_2_Cl_2_ exhibits fluorescence typical of a PDI (*λ*_em_ = 534 nm), with a small Stokes shift (*υ*_abs_ – *υ*_em_ = 321 cm^–1^), and vibronic features (Fig. 3a and Table S1†). The excitation of **4** in CH_2_Cl_2_ solution at 490 nm gives a broad, featureless spectral profile (Fig. 3b) centred at *λ*_em_ = 627 nm and slightly enhanced fluorescence (*Φ* = 0.016) relative to **1**. The lack of vibronic features, larger Stokes shift (*υ*_abs_ – *υ*_em_ = 2883 cm^–1^) and an enhancement in quantum yield are characteristics that have been attributed to emission from an excimer-like state in π-stacked molecules in which the π-systems are electronically coupled in the excited state.^50^ Furthermore, emission from **4** is sensitive to solvent polarity hence increasing solvent polarity, from that of CH_2_Cl_2_ (or CHCl_3_) to MeCN, increases the Stokes shift (*υ*_Abs_ – *υ*_Em_ = 3672 cm^–1^) and red-shifts the emission band to 655 nm. Reducing the solvent polarity leads to a significant drop in excimer intensity hence in THF the excimer band is very weak (Fig. S27†).

In contrast to the PDI–PDI handcuff rotaxane, **4**, the emission profile of the NDI–PDI system, **5**, in CH_2_Cl_2_, when excited at 490 nm, contains vibronic coupling and is weak (*λ*_em_ = 544 nm and *Φ* = 0.004) (see ESI, Fig. S28 and Table S1†). Emission at this wavelength is consistent with the weak emission observed for precursor **1**, discussed above, from a localised PDI based orbital. No evidence for emission from an excimer-like state was observed in either CH_2_Cl_2_ or MeCN. The absence of an excimer-like state may correlate with a lack of ground state π–π interactions between the PDI and NDI cores as inferred from absorption spectroscopy of **5**.

**Fig. 4:**
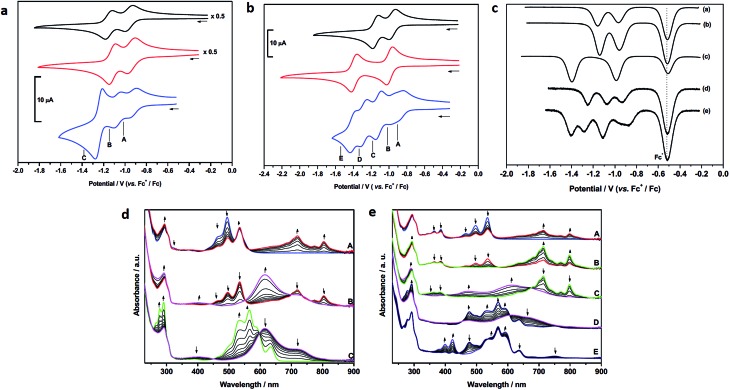
Cyclic voltammetry of: (a) **4** (blue trace) together with components **1** (black trace) and **6** (red trace); (b) **5** (blue trace) together with components **1** (black trace) and **7** (red trace). Points marked A–E represent potentials used in (spectro)electrochemical reduction experiments. Cyclic voltammograms were recorded at ambient temperature and 0.10 V s^–1^. (c) Square wave voltammetric data for: (a) **1**; (b) **6**; (c) **7**; (d) **4**; (e) **5**. The major peak at –0.52 V corresponds to the potential of the [(η^5^-C_5_Me_5_)_2_Fe]^+^/[(η^5^-C_5_Me_5_)_2_Fe] couple used as the internal standard. (d) UV-vis absorption spectra showing the inter-conversion of redox states between **4** and [**4**]^–^ (A); [**4**]^–^ and [**4**]^2–^ (B); [**4**]^2–^ and [**4**]^4–^ (C). (e) UV-vis absorption spectra showing the inter-conversion of redox states between **5** and [**5**]^–^ (A/B); [**5**]^–^ and [**5**]^2–^ (C); [**5**]^2–^ and [**5**]^3–^ (D); [**5**]^3–^ and [**5**]^4–^ (E). Arrows indicate the progress of reduction. See parts (a) and (b) for potentials applied. All spectroscopic measurements in CH_2_Cl_2_ containing [^*n*^Bu_4_N][BF_4_] (0.4 M) as supporting electrolyte at 273 K.

Cyclic (CV) and square-wave (SW) voltammetry and spectroelectrochemistry were used to probe how the proximity of the two rylene diimides in **4** and **5** affects the molecular orbitals of each species at each accessible oxidation state. The cyclic voltammograms of precursor **1** and model compound **6** are similar both in potentials and profiles and consistent with other PDIs unsubstituted in the bay region,^51^ (Table 1), each showing two one-electron reductions separated by *ca.* 0.2 V (Fig. 4).

**Table 1:** Cyclic Voltammetry (CV) and Square Wave (SW) data for compounds **1**, **4–7**^*a*^

Compound	1^st^ reduction		2^nd^ reduction		3^rd^ reduction		4^th^ reduction	
	CV, *E*_1/2_/V	SW/V	CV, *E*_1/2_/V	SW/V	CV, *E*_1/2_/V	SW/V	CV, *E*_1/2_/V	SW/V
**1**	–0.96 (0.07)	–0.97	–1.15 (0.07)	–1.16	—		—	
**4**	–0.93 (0.08)	–0.94	–1.07 (0.07)	–1.07	–1.25 (0.07)^*c*^	–1.25^*c*^	—	
**5**	^*b*^	–0.88^*d*^	–1.12 (0.07)	–1.11	–1.28 (0.08)	–1.28	^*b*^	–1.40
**6**	–0.94 (0.07)	–0.97	–1.12 (0.06)	–1.14	—		—	
**7**	–0.99 (0.07)	–0.99	–1.39 (0.06)	–1.40	—		—	

^*a*^Recorded in CH_2_Cl_2_ containing [^*n*^Bu_4_N][BF_4_] (0.4 M) as supporting electrolyte, at ambient temperature. Potentials quoted at 0.10 V s^–1^ against *E*_1/2_ Fc^+^/Fc. Values in brackets are Δ*E* (= *E*ap – *E*cp).

^*b*^Peak potentials were not resolved (see square voltammetric data).

^*c*^Corresponding to the formation of [**4**]^4–^.

^*d*^Additional unresolved shoulder to negative potential.

In contrast to **1** and **6**, the CV of **4** consists of three distinct redox couples indicating interactions between the two redox active PDI sites. The first and second reductions are of similar magnitude and assigned as two one-electron reversible reductions, whilst the third is significantly larger and assigned as a two-electron process. Thus, for **4** the addition of the first electron to each PDI occurs at two separate potentials (–0.93 and –1.07 V). The third reduction process of **4**, at –1.25 V, corresponding to the addition of a second electron to each PDI, is shifted more negative than the analogous reductions in **1** and **6**, respectively, and appears as a single process. The small shift to positive potential in the first reduction of **4** relative to components **1** and **6** suggests stabilisation of [**4**]^–^ in comparison to [**1**]^–^, or [**6**]^–^, and this is consistent with the electron occupying a more extended molecular framework, although the magnitude of shift indicates that the effect of this stabilisation is small.^52^ The addition of a second electron to give [**4**]^2–^ occurs at –1.07 V and this is more negative than the first reductions in **1** and **6**. Remembering that the reductions in [**4**]^2–^, [**1**]^–^ and [**6**]^–^ all correspond to one electron per PDI, this destabilisation of [**4**]^2–^ relative to [**1**]^–^, or [**6**]^–^, is unsurprising given the small stabilisation noted for [**4**]^–^ and the additional energy required to overcome the repulsive (coulombic) interactions introduced as a result of adding a second electron into this already occupied molecular framework. The extent of interaction between electrons in the molecular orbital of [**4**]^–/2–^ is given by the difference in redox potentials for the first and second reduction (Δ*E* = *E*_2_ – *E*_1_). A difference of 0.14 V between the first and second reductions in **4** gives a comproportionation constant, *K*_com_ (= 10^(*F*Δ*E*/2.303*RT*)^), of *ca.* 10^2^. On the electrochemical timescale this indicates that **4** is a Robin–Day Class II mixed valent species and that the extent of communication is limited.

In contrast to the first and second reductions of **4** addition of (two) subsequent electrons, to give [**4**]^4–^, occurs in a single reduction step. The reduction occurs at –1.25 V and is more negative than the corresponding reductions that generate [**1**]^2–^, or [**6**]^2–^, each corresponding to the addition of two electrons per PDI. This shift to negative potential is consistent with the electrons populating an extended molecular orbital, as noted for the first two reductions but now the interaction between the PDI centres has changed. It is noted that the couple exhibits scan rate dependence and, at slower scan rates (0.20 V s^–1^) the peak separation drops to 0.05 V, significantly less than that of the ferrocene couple under identical conditions (0.07 V). Such behaviour in electrochemical EE-type mechanisms occurs when there are attractive interactions on addition of a second electron (in this case between the addition of the 3^rd^ and 4^th^ electron) and this generally requires significant structural change or large solvation or ion-pairing effects.^53^ This difference may be a consequence of the non-rigid framework supporting the PDI centres whereby reorientation of PDIs occurs in the tetra-anionic species in order to minimise repulsive interactions.

The redox behaviour exhibited by **4** is distinct from that observed for a comparatively rigid PDI–PDI dimer described by Würthner and co-workers^21^ in which two PDIs are separated by diacetylene spacers. As a result of the rigid framework that separates the two PDI moieties a chemical reaction is observed following the third reduction process which is related to the separation and simultaneous opening of the PDI–PDI cyclic structure. This opening process in turn leads to the fourth reduction taking place at a (formally) more positive potential due to more open PDI–dimer structure. Thus, the third and fourth reduction processes of the rigid PDI–dimer system are electrochemically irreversible in contrast to our more loosely-bound handcuff structure that enables the PDI moieties to reposition upon reduction. The redox behaviour of **4** also differs significantly from that obtained from a cyclophane molecule consisting of two NDI moieties cofacially stacked in a rigid framework, in which a greater level of delocalisation was noted and significant interactions through the π-based molecule orbital were observed up to the tetra-anionic state in CV experiments.^27^

The CV of **5** (Fig. 4b) is significantly more complex than that of **4** and shows a series of five reduction steps, with the first and second reductions, centred at *ca.* –0.9 V, appearing as closely overlapped processes of similar intensity to each other but smaller than the subsequent reductions. The onset of reduction in **5** occurs at a potential less negative than those of the first reductions in reference compounds **1** and **7** suggesting some molecular interaction between the PDI and NDI on the CV timescale; the subsequent reductions fall within the potential range of reductions found in **1** and **7**.

The assignment of these reductions based on CV alone is problematic, however, PDI, [PDI]^–^, NDI and [NDI]^–^ all have very distinctive bands in their UV/vis spectra, therefore we have probed the progress of reduction in **4** and **5** using results from spectroelectrochemical measurements. UV-visible measurements gave significant insight into the nature of each redox state which was further supported by near- and mid-IR spectroscopic measurements which are discussed further in the ESI.† Each component (**1**, **6**, **7**) was studied and revealed a typical profile associated with each reductive process (Figs. S29–S31†).^54^,^55^ Thus, following the first reduction, forming [PDI/NDI]^–^, a significant bathochromic shift is observed for the absorption maxima followed by a hypsochromic shift following the second reduction to the dianion [PDI/NDI]^2–^. The PDI–PDI handcuff, **4**, exhibits quite distinct behaviour (Fig. 4d). As noted above, the UV-visible spectrum of the neutral handcuff is consistent with the formation of an H-aggregate within the handcuff. Following the first reduction, to give [**4**]^–^, the UV-visible spectrum is consistent with the spectrum anticipated for a PDI monoanion^54^ and a, non-aggregated, neutral PDI species suggesting that the added electron is localised on a single PDI and that aggregation is disrupted upon reduction. This observation is further confirmed by the FT-IR spectrum of [**4**]^–^ (see Fig. S36 and Table S3†) with anticipated^54^ shifts to lower energy for carbonyl bands upon reduction of the PDI moieties. The second reduction process, to give [**4**]^2–^ gives rise to a markedly different UV-visible spectrum with a large, broad, absorption observed (*λ*_max_ = 615 nm). The profile of this spectrum contrasts strikingly with the spectrum of a typical PDI monoanion or dianion^54^ and led us to investigate the NIR spectra of both [**4**]^–^ and [**4**]^2–^. In the case of [**4**]^2–^ a strong and broad absorption was observed in the NIR (*λ*_max_ = 1527 nm) (Fig. S37†); no such NIR absorption was observed in the spectrum of [**4**]^–^. Similarly to spectra for [**4**]^–^, the FT-IR spectrum of [**4**]^2–^ (Fig. S36†) exhibits the anticipated spectra for a direduced PDI^54^ but also reveals the growth of an unexpected small and broad absorption at 1818 cm^–1^. Following the third, two-electron, reduction the UV-visible spectrum of [**4**]^4–^ was consistent with the spectrum normally observed for PDI dianions.^54^ The spectra indicate that upon complete reduction of the PDI–PDI handcuff each PDI moiety is present as a dianion, *i.e.* [PDI]^2–^–[PDI]^2–^, with no significant communication between the two aromatic moieties. This observation confirms sufficient handcuff flexibility to allow the charged PDI^2–^ groups to repel each other.

For **5**, reduction at potential A (Fig. 4b) results in a *ca.* 50% loss of intensity for bands associated with PDI and the corresponding formation of bands of [PDI]^–^ (see Fig. S34, ESI†). Noting that for reference compounds **1** and **7** the PDI and NDI reductions occur at very similar potentials, it might be expected that distinct bands for [NDI]^–^ (Fig. S34†) would also be observed. This is not the case, although some loss of intensity is seen in the parent NDI bands. Reduction at potential B sees a complete loss of PDI bands in favour of [PDI]^–^ bands (Fig. 4e), further broadening of NDI bands but no generation of characteristic [NDI]^–^ bands. This suggests that the two small waves noted in the CV are associated with a localized one-electron PDI-based reduction. We suggest that the first electron added to **5** is shared between two species, explaining the lower than expected current for each of the first two reductions. Due to the presence of handcuff rotaxane stereoisomers (see below) an equilibrium between interacting (easier to reduce than **1**, see above) and non-interacting (reduction at a similar potential to that in **1**, Fig. 4c) PDI and NDI π-systems, is present leading to the complex behaviour of the first reduction process.

Further reduction, corresponding to the second reduction of **5**, to [**5**]^2–^, results in the loss of NDI and [PDI]^–^ bands and these are replaced by a broad feature at 612 nm and a new, broad band at *ca.* 1516 nm is developed in the NIR region (Fig. S37†). Further reduction at potential D, to give [**5**]^3–^, results in the loss of the broad features of [**5**]^2–^ and produces a spectral profile containing bands broadly characteristic of [**7**]^–^ and [**1**]^2–^. This indicates that the electron enters a PDI based orbital and on this timescale the electrons now occupy localised PDI or NDI based π-systems, *i.e.* [PDI]^2–^–[NDI]^–^. Reduction at potential *E*, to give [**5**]^4–^, results in the loss of the 751 nm band and features associated with [**7**]^–^ and produces a spectral profile containing bands characteristic of [**1**]^2–^ and [**7**]^2–^*i.e.* [PDI]^2–^–[NDI]^2–^.

EPR spectroscopy was used to further probe the localisation of charge in [**4**]^–^ and [**4**]^2–^, [**5**]^–^, [**5**]^2–^ and [**5**]^3–^. The results are discussed in depth in ESI† but are summarised here.

The electrochemical reduction of **4** to [**4**]^–^ gave an EPR active species. The spectral width of [**4**]^–^ (9.4 G) was *ca.* 72% that of [**1**]^–^ (13.0 G) and the spectrum was less well resolved (Fig. S41†). Nevertheless, the smaller spectral width and the observable features in the spectrum could be reasonably reproduced by simulation using three sets of eight hydrogen atoms and four equivalent nitrogen atoms, each at a magnitude half that required in the simulation of [**1**]^–^. These results are consistent with the unpaired electron in [**4**]^–^ being either fully delocalised or hopping between the two PDI moieties on the EPR timescale. EPR spectra of [**4**]^2–^ reveals a loss of signal suggesting the presence of a singlet ground state, or possibly a triplet state although cooling the solution to 77 K failed to provide evidence for the latter. It is not uncommon for triplet states to not be observed by EPR spectroscopy.^56^–^58^ The EPR spectra of [**5**]^–^ (Fig. S42†) indicates localisation of the unpaired electron in the π-based orbital of the PDI component, although a small degree of delocalisation (or hopping) to NDI may also be possible on this timescale. As with [**4**]^2–^, the EPR spectrum of [**5**]^2–^ suggests a majority presence of a ground state singlet, resulting from strong electron spin coupling and consistent with the electrons entering an orbital in a shared π-based molecular framework. Again, this observation is consistent with results from UV/vis spectroscopy for [**5**]^2–^. Further reduction to [**5**]^3–^ results in a return of EPR activity (Fig. S42†) whereby a spectral profile consistent with the presence of an NDI monoanion is observed. The spectral width and parameters used in the simulation of [**5**]^3–^ indicate that the unpaired electron now occupies a localised NDI based orbital, *i.e.* [PDI]^2–^–[NDI]^–^.

The behaviour of [**4**]^2–^ is unique and differs from that observed for Würthner and co-workers' rigid PDI dimer.^21^ We envisage two scenarios to explain the spectra of this species, the presence of [PDI]^–^–[PDI]^–^ or [PDI–PDI]^2–^. The former, in which electrons are localised on each PDI unit should give a spectral profile similar to those of [**1**]^–^ and [**6**]^–^, which is not the case. The spectrum is also not consistent with the formation of a σ-bonded PDI dimer.^59^ Thus, on the basis of the reversible cyclic voltammetry and the EPR spectra for [**4**]^2–^ we attribute the spectral features of [**4**]^2–^ to the presence of a π-dimer, *i.e.* [PDI–PDI]^2–^ in which the electrons occupy a molecular based orbital manifold.^27^,^60^ A theory further supported by DFT calculations discussed below.

**Fig. 5:**
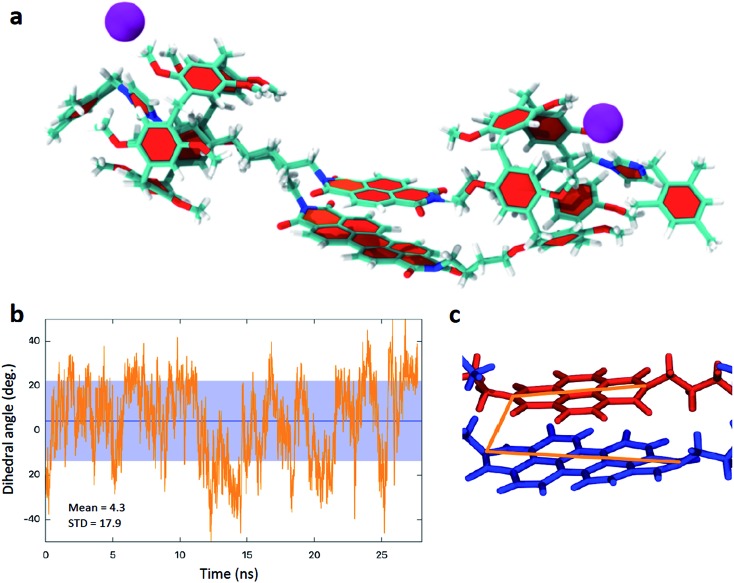
(a) Model of **5** from molecular dynamics calculations (also see ESI†) illustrating the co-planar rylene diimide arrangement. (b) Calculated variation in the dihedral angle adopted between the principal axes of the two rylene diimdes (shown in c) in **5**.

The unusual behaviour associated with the first reduction of **5** led us to investigate the compound further. Our results suggested that addition of the first electron added to **5** is shared between two species, involving interacting and non-interacting PDI and NDI π-systems. Close inspection of the ^1^H NMR spectra of **4** and **5** indicates the presence of stereoisomers of the handcuff rotaxanes.^7^ As the imidazolium arm can enter through either aperture of the pillararene, it is possible to prepare handcuff rotaxanes with head-to-head, tail-to-tail, or head-to-tail arrangements.^7^ In order to evaluate the conformational arrangement of the structures molecular dynamics modelling of **5**, suspended in CHCl_3_ solution, was undertaken (Fig. 5 and ESI†). For the most stable head-to-head isomer it can be readily seen from calculations, over hundreds of nanoseconds timescale, that the central rylene diimide cores adopt the anticipated co-facial arrangement, and that even during vibrations of the handcuff rotaxanes the aromatic species do not move significantly away from this preferred arrangement (see ESI† for movie illustrating the calculated motion of the handcuffs). For **4** there is no evidence from (spectro)electrochemical measurements that a co-facial arrangement is lost during the first reduction process, however, this may not be the case for **5** which is anticipated to have a weaker π-interaction between the PDI and the smaller NDI groups. Thus, we investigated the behaviour of the head-to-head (**5a**), tail-to-tail (**5b**), or head-to-tail arrangements (**5c**) (Fig. S45†). The head-to-head isomer **5a** was found to be the most stable conformation (–134.4 kcal mol^–1^) in comparison to the **5b** (–125.3 kcal mol^–1^) and **5c** (–122.2 kcal mol^–1^). The calculated dynamics of the tail-to-tail and head-to-tail isomers indicate that there is a tendency for the two rylene diimides to exhibit strong structural distortions, potentially removing face-to face interactions (see ESI† for further details). This is consistent with our conclusions with respect to the first reduction process associated with **5**, suggesting that both interacting and non-interacting π-systems are present in this handcuff.

The molecular dynamics modelling also indicates that for the head-to-head isomers although the two rylene diimide cores do not separate, the interplanar distance remaining consistent, there is a twisting motion such that the principal axes of the rylene diimides (defined as the axis that joins the two nitrogen atoms of the terminal imides, see Fig. 5c) rotate with respect to one another. However even this does not vary widely with an oscillation of the dihedral angle adopted between the principal axes between ±40° (**4**) and ±50° (**5**) with mean values of 6.65° ± 17.9° for **4** and 4.3° ± 17.9° for **5** (Fig. 5b).

The nature of the UV/vis spectra observed for **4** and **5** was investigated further using DFT calculations. Details of the calculations and assignment of the transitions observed for PDI and NDI models is discussed in ESI.† DFT modelling of **4** yielded results in which the calculated spectra are consistent with experiment for all charge states. Thus, for the neutral PDI–PDI dimer, **4**, the calculations predict a single band at 529 nm (Fig. S50†). This arises from the HOMO to LUMO transition that can be localized on either PDI molecule. For [**4**]^–^ the calculations confirm our experimental observations that the lower energy bands at 795 nm and 651 nm are associated with the reduced [PDI]^–^ species while the band at 502 nm corresponds to the HOMO to LUMO transition on the neutral PDI molecule (Fig. S51†). Perhaps the most interesting insight is into the nature of transitions observed for [**4**]^2–^ which has a highly unusual UV/vis spectrum with includes a broad band at 615 nm. DFT calculations indicate that [**4**]^2–^ adopts a state in which there is an unpaired electron on each molecule and the spin density is spread across both molecules (Fig. S52†). The unusual broad band in the UV-visible spectrum is assigned to excitation of one of these unpaired electrons to the LUMO (Fig. S53†). This state could have singlet or triplet spin; a triplet state was not observed by EPR spectroscopy but as noted above this is not uncommon for such states.^56^–^58^

DFT calculations for face-to-face PDI–NDI dimers, as observed in **5**, are also consistent with observed spectra. Thus, for the neutral species two bands are predicted which arise from the HOMO to LUMO transitions on PDI (492 nm) and NDI (343 nm) (Fig. S55†). Calculations for the mono-reduced species, [**5**]^–^, indicates that the additional electron is based on the PDI moiety, leading to a spectrum with an absorption, at 343 nm, associated with neutral NDI and bands at higher wavelength arising from the mono-reduced PDI (Fig. S56†). As with [**4**]^2–^, the direduced PDI–NDI species, [**5**]^2–^, has an unpaired electron on both PDI and NDI groups. The computed spectrum shows the bands associated with the mono-reduced state of each molecule (Fig. S57†).

## Conclusions

Through the design of a handcuff structure we have been able to create dimers of rylene diimides such that the two components are able to interact but are not constrained in their geometries by rigid covalent linkers. Thus, we are able to probe the nature of interactions between π-orbital manifolds held in close proximity. As a result of these close interactions we are able to observe unusual behaviour including the formation of an H-aggregate (an interaction not seen in the solution spectra of precursor molecules **1** and **2** at equivalent concentrations) and this interaction appears to extend into the excited state where fluorescence characteristic of an excimer-like state is observed and which shows solvent dependence. By contrast **5** shows neither of these characteristics. Interaction between the two π-systems in both **4** and **5** is clearly demonstrated by cyclic voltammetry, but these interactions are weaker than those found in rigidly linked units and appear to be modulated by the redox state of the molecule.

Reduction of the handcuffs reveals intriguing behaviour which further reinforces our understanding of the nature of the interactions between rylene diimides. Thus, mono-reduced **4** is paramagnetic and exhibits properties of full delocalisation (or of fast hopping) between the two PDI centres on the EPR timescale however the absence of a clear intervalence charge transfer band in the UV/vis/NIR region, together with bands associated with both neutral and monoreduced PDI in the spectrum of [**4**]^–^, suggests that the electron is unlikely to be delocalised on this faster, electronic transition, timescale. In contrast there is little direct evidence for the extensive delocalisation of the electron across the π-systems in [**5**]^–^ on the EPR timescale or faster.

DFT calculations successfully model the UV-visible spectrum for di-reduced **4**, [**4**]^2–^, which unusually exhibits a broad absorption observed at 615 nm, which is characteristic of neither mono nor di-reduced PDI monomers. In combination with EPR, cyclic voltammetric measurements and DFT calculations we conclude that the behaviour of [**4**]^2–^ is consistent with the formation of a π-dimer. Analogous bands in the electronic spectrum of [**5**]^2–^ are evidence for π–π interactions between NDI and PDI based orbitals. Further reduction of **4** leads to a fully reduced [**4**]^4–^ species whose spectroscopic and electrochemical profiles are consistent with non-interacting [PDI]^2–^ species, reflecting the flexible nature of the interlocked structure. Reduction of **5** leads to formation of a paramagnetic state in [**5**]^3–^ with an unpaired electron localised on the NDI group, [PDI]^2–^–[NDI]^–^, illustrating the ability to sequentially add electrons to specific components of the interlocked molecule through simple combinations of electron accepting sites. As with **4**, reduction to [**5**]^4–^ results in non-interacting [PDI]^2–^ and [NDI]^2–^ species as a result of the flexibility of the interlocked structure.

Our strategy demonstrates that it is possible to use handcuff structures to create and to probe the interactions between pairs of redox active molecules. We have successfully demonstrated that due to the flexibility of the interlocked arrangement, in comparison to more rigid covalently coupled structures, we observe unusual states, notably for the PDI–PDI systems, **4**. We believe that our approach provides a new strategy to allow systematic investigations between molecules placed in close proximity and that our approach is potentially widely applicable to other species of interest.

## Conflicts of interest

There are no conflicts to declare.

## Supplementary Material

Supplementary movie

Supplementary movie

Supplementary information
