# Supplementary material for: Synthesis and characterisation of rylene diimide dimers using molecular handcuffs
Source: Chem Sci. 2019 Feb 15;10(13):3723–32. doi: 10.1039/c9sc00167k (PMC6457202; doi:10.1039/c9sc00167k)
Supplement: Supplementary file 3 [file SC-010-C9SC00167K-s003.pdf]

Electronic Supplementary Material (ESI) for Chemical Science.
This journal is © The Royal Society of Chemistry 2019

Supporting Information for:

Synthesis and Characterisation of Rylene Diimide Dimers Using Molecular Handcuffs

Lixu Yang, Philipp Langer, E. Stephen Davies, Matteo Baldoni, Katherine Wickham, Nicholas A. Besley, Elena Besley and Neil R. Champness*

Supporting Information Table of Contents

Synthetic Methods Page 1

Electrochemical and Spectroelectrochemical Measurements Page 19

Near- and Mid-IR Spectroscopy Page 28

Further UV-visible Spectra Page 30

EPR spectroscopy Page 32

Molecular Dynamics Simulations of the Stability and Dynamic Behaviour Page 35

DFT Calculations Page 37

References Page 45

Synthetic Methods

All chemicals and dry solvents were purchased from Sigma-Aldrich or VWR. NMR spectra were recorded at room temperature using Bruker DPX400, AV400, AV(III)400 or AV(III)500 instruments. Deuterated solvents were used as specified in this section. Chemical shifts were recorded referenced to solvent residue. Mass Spectrometry were recorded using Bruker microTOF II, JEOL GCv4G, Bruker Impact II or Bruker Ultraflex III. Column chromatography was performed on silica gel 60Å. Size exclusive column chromatography was performed on Bio-beads SX-3. UV-Vis spectra were recorded using PerkinElmer Lambda 25 UV/Vis spectrometer in HPLC grade dichloromethane. Variable temperature UV/vis spectra were recorded on a PerkinElmer Lambda16 spectrometer and temperature control was achieved using a PerkinElmer PTP 1+1 Peltier system.

1
2
3
4
5
6
7

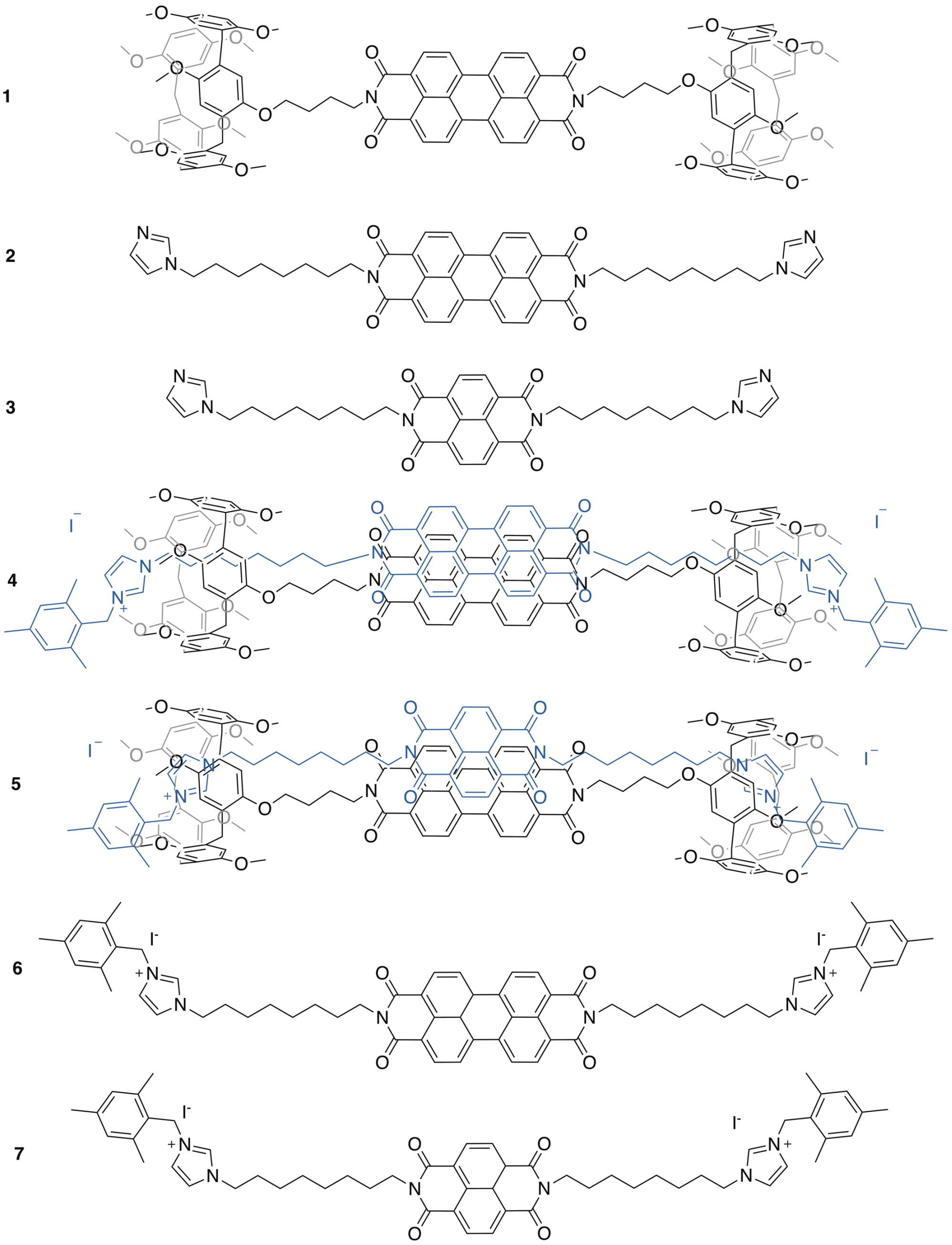

Figure S1. Compounds **1**-**7** as discussed in the main manuscript.

Compound 8[S1], 9[S2] and 2,4,6-trimethylbenzyl iodide[S3] were prepared according to literature procedures.

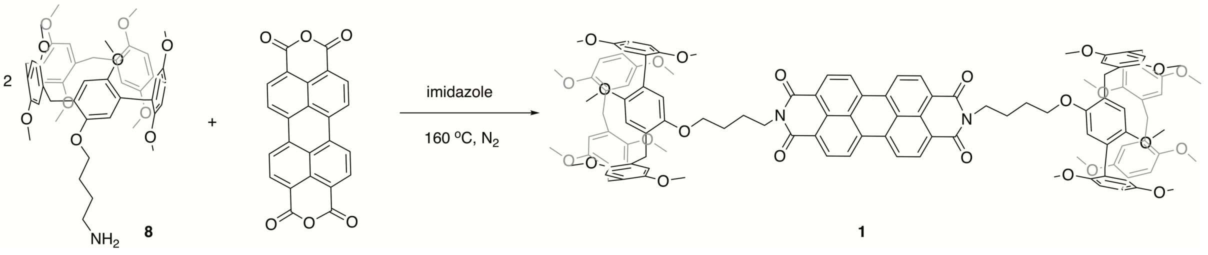

Figure S2. Synthetic route for compound **1**.

Synthesis of **1**: Compound **8** (410 mg, 0.508 mmol), perylene-3,4,9,10-tetracarboxylic dianhydride (98 mg, 0.25 mmol) and imidazole (600 mg) was added to a flask and degassed. The mixture was heated to 160°C under dinitrogen overnight. After cooling down, 2M HCl solution (20 ml) was added. The solution was extracted with CH_2Cl_2. The organic layer was washed with water twice, dried over $MgSO_4$ and filtered. Solvent was removed *in vacuo*. The residue was subject to chromatography with $CHCl_3$/MeOH=200:1. The resulting product, compound **1**, was obtained as red solid (400 mg, yield 81%). ^{1}H NMR (400 MHz, $CDCl_3$) δ ppm 8.70 (d, *J* = 8.0 Hz, 4H), 8.61 (d, *J* = 8.1 Hz, 4H), 6.78 - 6.68 (m, 20H), 4.36 (t, *J* = 7.0 Hz, 4H), 3.93 (t, *J* = 6.1 Hz, 4H), 3.83 - 3.51 (m, 74H), 2.11 - 2.04 (m, 4H), 2.03 - 1.93 (m, 4H). ^{13}C NMR (101 MHz, $CDCl_3$) δ ppm 163.31, 150.84, 150.82, 150.72, 150.01, 134.42, 131.23, 129.16, 128.43, 128.31, 128.26, 128.20, 128.14, 128.10, 127.94, 126.18, 123.13, 122.96, 115.06, 114.25, 114.17, 114.15, 114.12, 114.09, 114.02, 114.01, 113.91, 68.14, 55.89, 55.85, 55.80, 55.75, 55.69, 55.67, 40.40, 29.79, 29.75, 29.72, 29.45, 27.55, 25.12. EI-HRMS m/z calcd for $[M]^{\cdot+}$ 1970.8069; found 1970.8147.

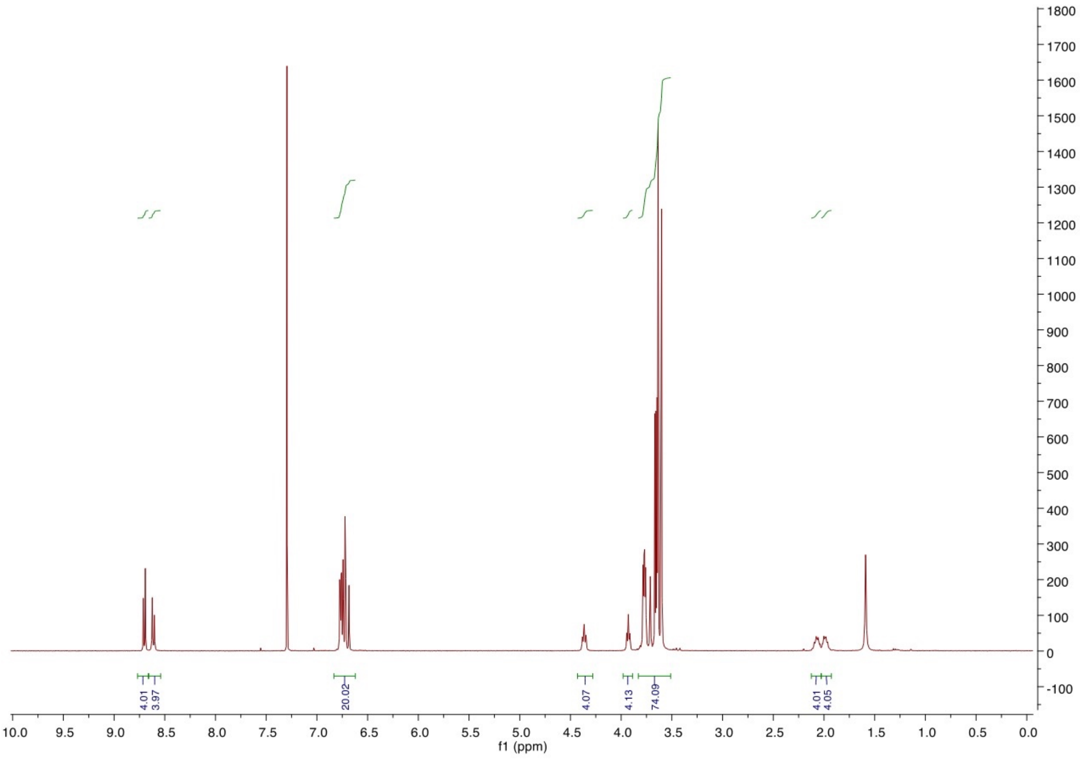

Figure S3. ^{1}H NMR (400 MHz, $CDCl_3$) spectrum of compound **1**.

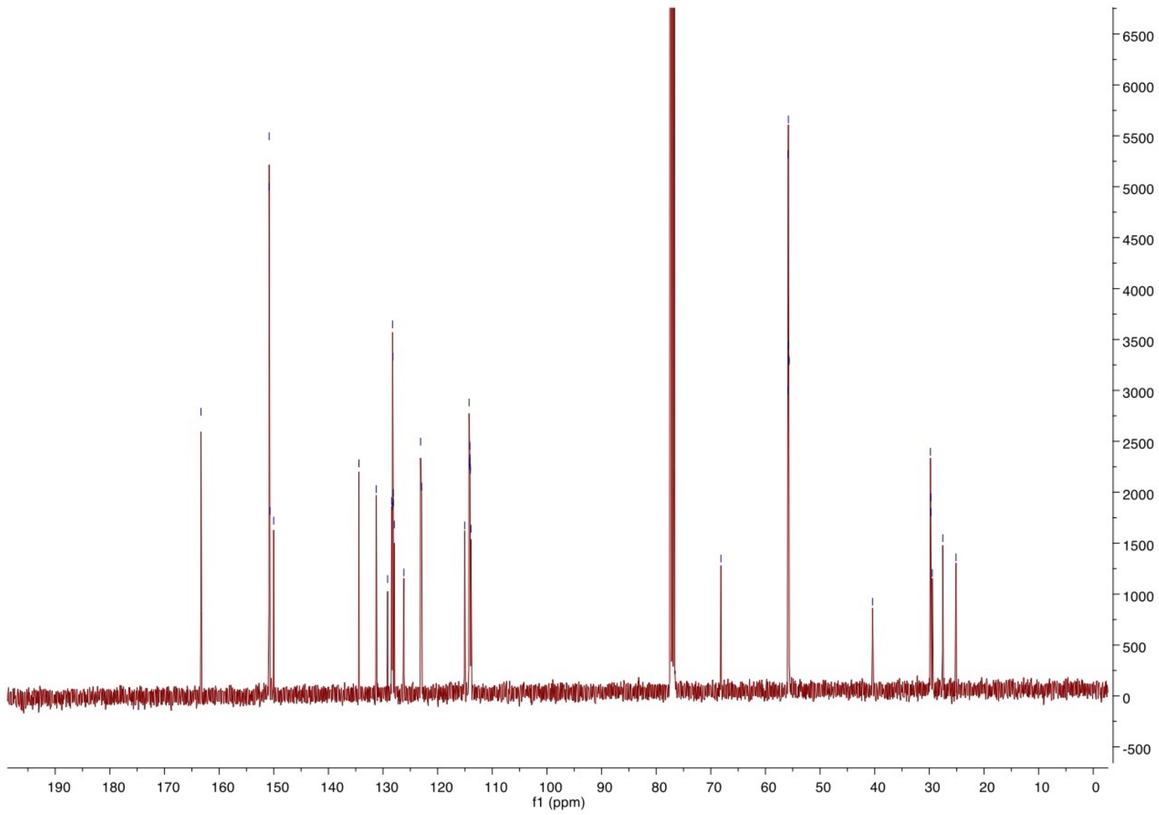

Figure S4. ^{13}C NMR (101 MHz, $CDCl_3$) spectrum of compound **1**.

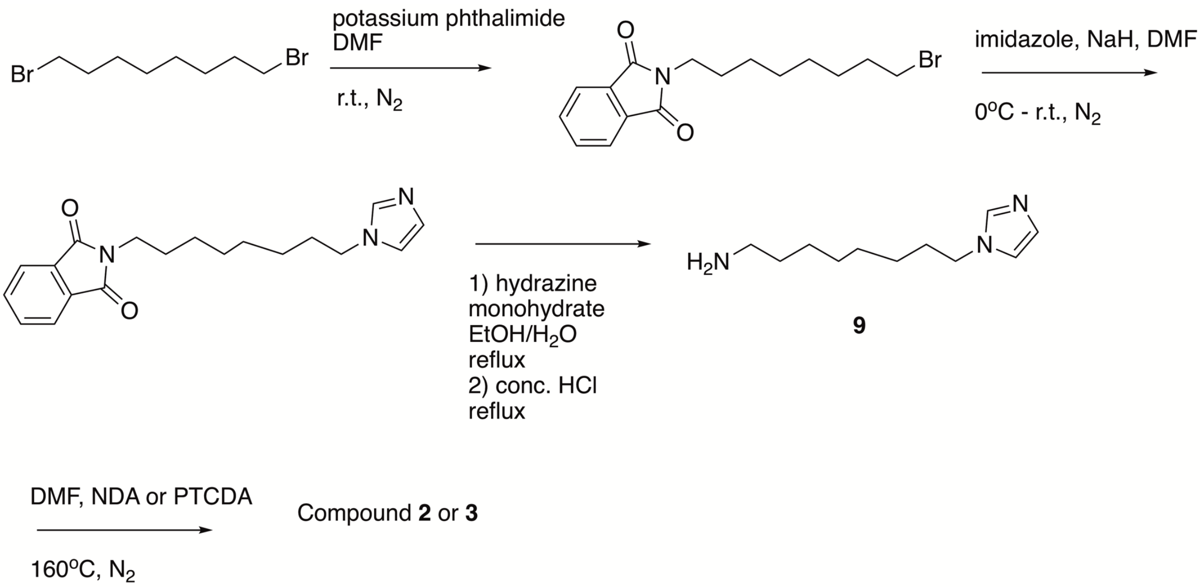

Figure S5. Synthetic route to **9** following the strategy of Gray *et al.* [S2]

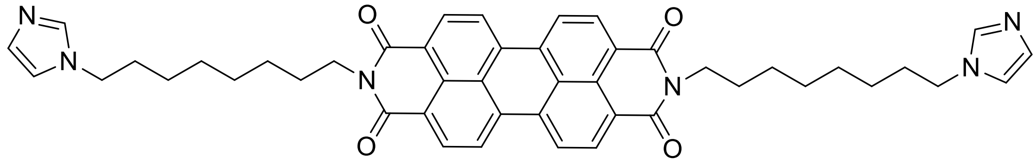

Synthesis of compound **2**: Perylene-3,4,9,10-tetracarboxylic dianhydride (157 mg, 0.4 mmol) and compound **9** (156 mg, 0.8 mmol) were mixed in 10 ml of DMF under a dinitrogen atmosphere. The mixture was heated to 160 °C for 24 h. After cooling to room temperature, the mixture was filtered and washed with acetone. The product, compound **2**, was obtained as red solid (241 mg, yield 80%). ^{1}H NMR (400 MHz, $CDCl_3$) δ ppm 8.66 (d, *J* = 8.0 Hz, 2H), 8.57 (d, *J* = 8.1 Hz, 2H), 7.50 (s, 1H), 7.08 (t, *J* = 1.0 Hz, 1H), 6.94 (t, *J* = 1.2 Hz, 1H), 4.28 - 4.15 (m, 2H), 3.96 (t, *J* = 7.2 Hz, 2H), 1.90 - 1.74 (m, 4H), 1.54 - 1.25 (m, 8H). ^{13}C NMR (101 MHz, $CDCl_3$) δ ppm 163.34, 137.08, 134.53, 131.36, 129.35, 129.29, 126.35, 123.28, 123.07, 118.80, 47.01, 40.58, 31.04, 29.05, 28.90, 27.96, 26.96, 26.48. ESI-HRMS m/z calcd for $[M+H]^+$ 747.3653; found 747.3629.

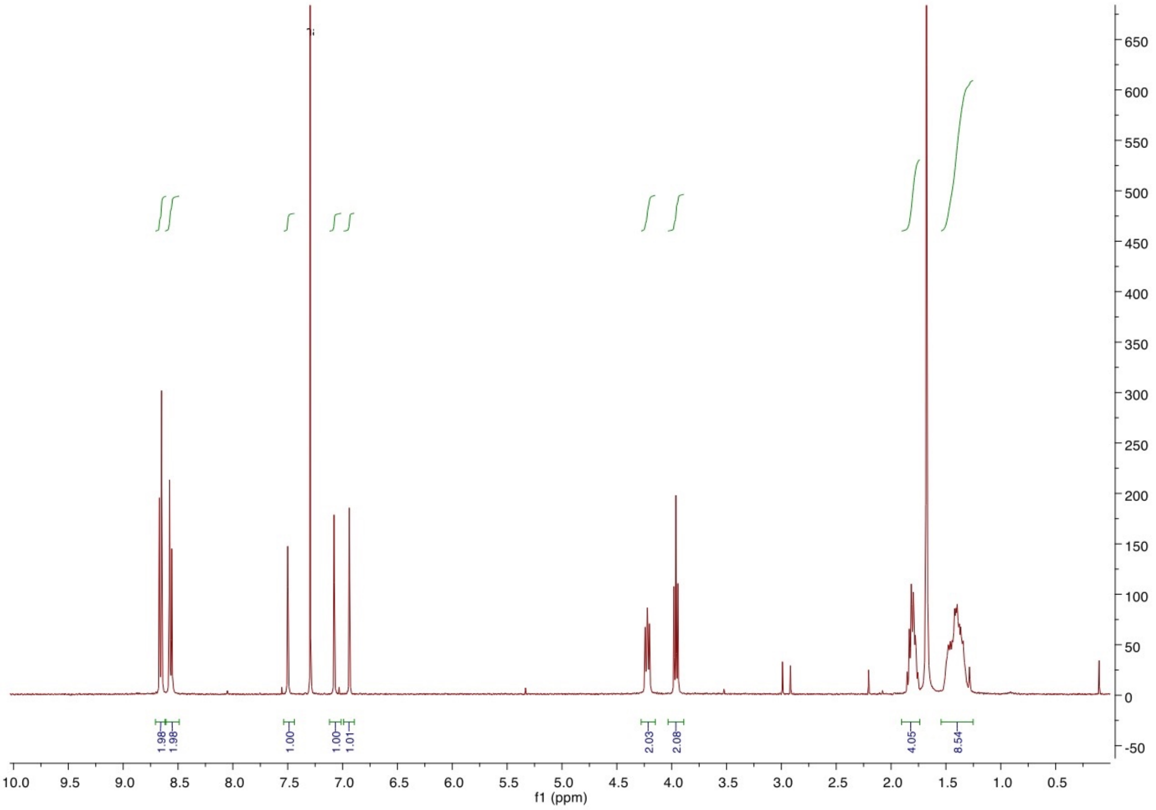

Figure S6. ^{1}H NMR (400 MHz, $CDCl_3$) spectrum of compound **2**.

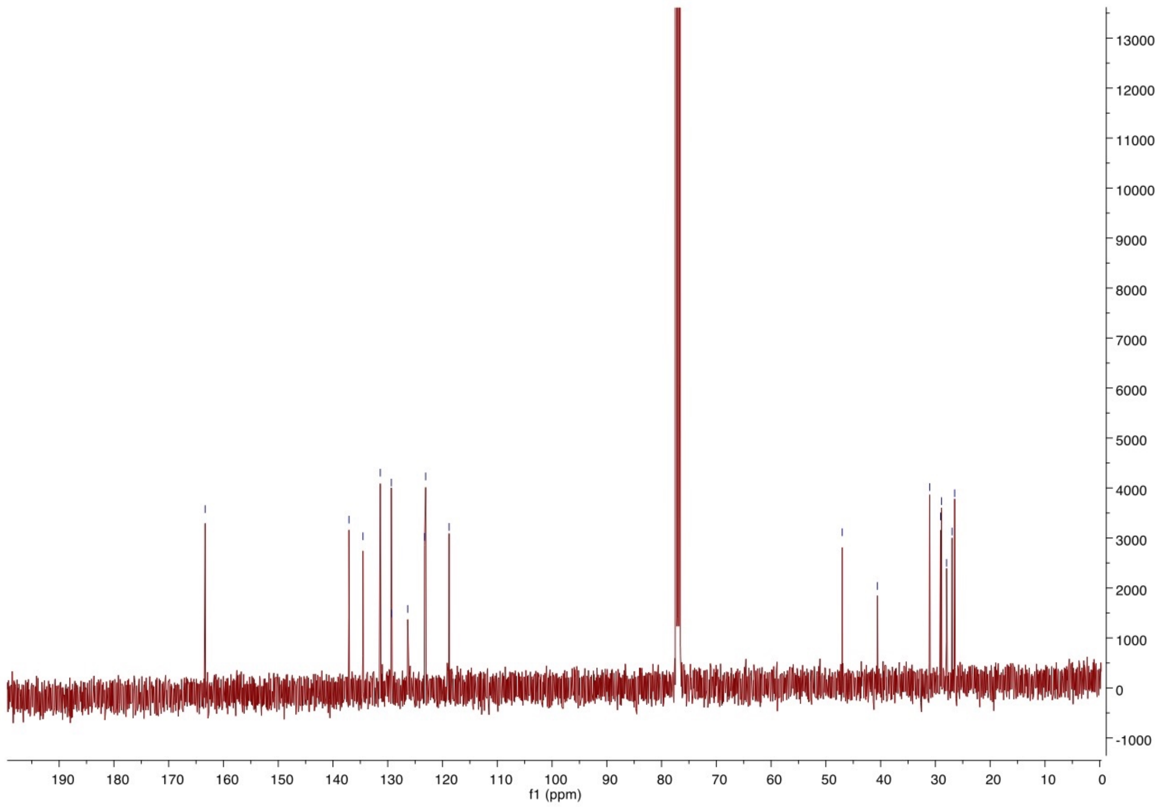

Figure S7. ^{13}C NMR (101 MHz, $CDCl_3$) spectrum of compound **2**.

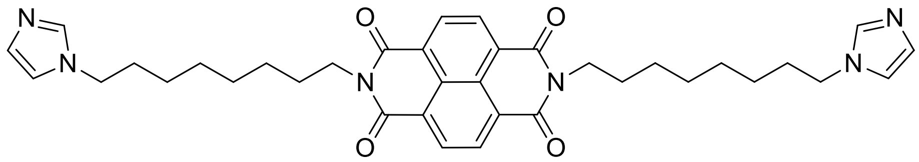

Synthesis of compound **3**: 1,4,5,8-Naphthalenetetracarboxylic dianhydride (107 mg, 0.399 mmol) and compound **9** (156 mg, 0.8 mmol) were mixed in 10 ml of DMF under a dinitrogen atmosphere. The mixture was heated to 160 °C for 24 h, cooled and filtered. The filtrate was evaporated to dryness. The residue was crystallized from $CHCl_3$ and *n*-hexane. The product, compound **3**, was obtained as brown solid (70 mg, yield 28%). ^1H NMR (400 MHz, $CDCl_3$) δ ppm 8.78 (s, 4H), 7.49 (s, 2H), 7.07 (t, *J* = 1.0 Hz, 2H), 6.93 (t, *J* = 1.2 Hz, 2H), 4.29 – 4.14 (m, 4H), 3.95 (t, *J* = 7.1 Hz, 4H), 1.83 – 1.67 (m, 8H), 1.50 – 1.27 (m, 16H). ^{13}C NMR (101 MHz, $CDCl_3$) δ ppm 162.86, 137.08, 130.98, 129.38, 126.65, 118.78, 46.99, 40.87, 31.03, 29.02, 28.88, 27.94, 26.89, 26.47. ESI-HRMS m/z calcd for $[M+H]^+$ 623.3340; found 623.3329.

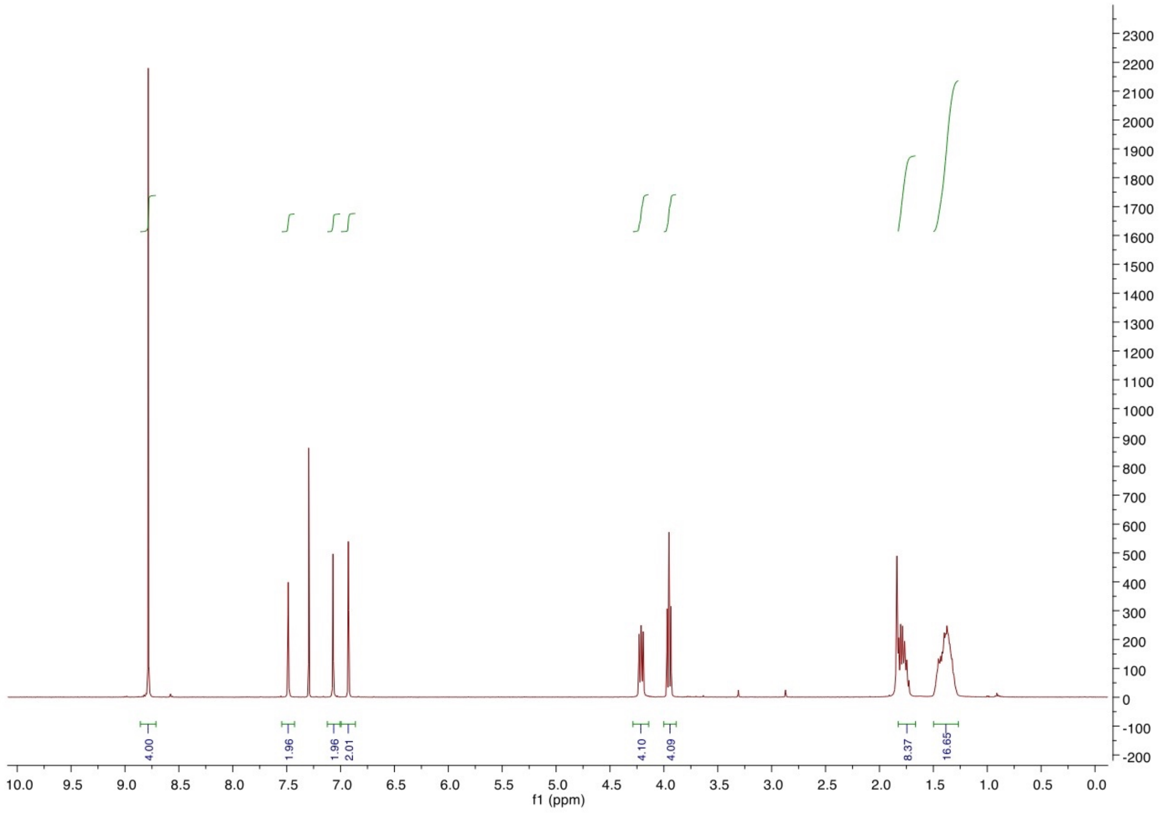

Figure S8. ^{1}H NMR (400 MHz, $CDCl_3$) spectrum of compound **3**.

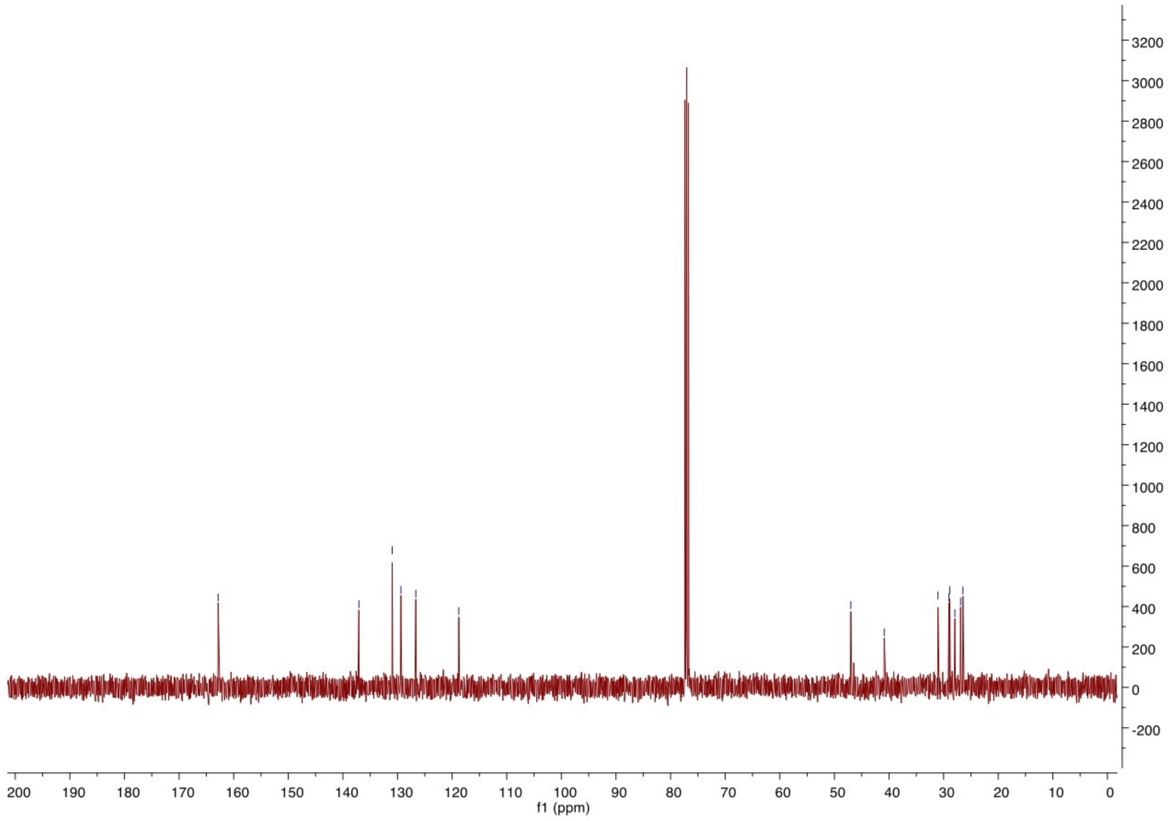

Figure S9. ^{13}C NMR (101 MHz, $CDCl_3$) spectrum of compound **3**.

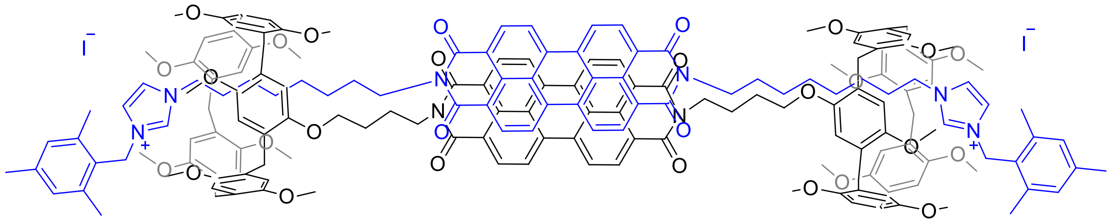

Synthesis of compound **4**: compound **1** (30 mg, 0.015 mmol) and compound **2** (12 mg, 0.016 mmol) were dissolved in dry $CHCl_3$ (1 ml) under a dinitrogen atmosphere and the mixture cooled to -10°C using an ice/salt bath. 2,4,6-trimethylbenzyl iodide (10 mg, 0.038 mmol) was added to the solution in the absence of light. The reaction mixture was slowly warmed to room temperature and stirred for 3 days. The crude product was purified by size exclusive chromatography using a $CHCl_3$ eluent and then by column chromatography on silica gel with CH_2Cl_2/MeOH=20:1. Compound **4** was obtained as a red solid (39 mg, yield 81%). ^{1}H NMR (400 MHz, $CDCl_3$) δ ppm 8.11 - 7.80 (m, 18H), 7.55/7.46 (s, 2H), 7.07 (s, 4H), 7.04 - 6.65 (m, 20H), 6.09/6.01 (s, 2H), 5.87 (dd, *J* = 15.0, 2.3 Hz, 2H), 5.70 (dd, *J* = 15.0, 7.3 Hz, 2H), 4.22 - 3.39 (m, 86H), 2.48 (s, 12H), 2.40 (s, 6H), 2.18 - 1.96 (m, 8H), 1.87 - 1.70 (m, 4H), 1.47 - 1.32 (m, 4H), 1.01 (br, 4H), 0.76 - 0.64 (m, 4H), 0.15 - 0.05 (m, 4H), – 0.83 - – 0.92 (m, 4H), – 1.41 - – 1.69 (m, 4H). ^{13}C NMR (101 MHz, $CDCl_3$) δ ppm 162.46, 162.44, 151.46, 151.30, 151.22, 150.78, 150.70, 150.35, 150.29, 150.25, 150.24, 150.16, 140.06, 138.06, 134.34, 133.03, 132.99, 132.79, 132.74, 132.41, 132.31, 130.36, 130.32, 130.20, 130.12, 130.08, 129.96, 129.78, 129.73, 129.04, 128.95, 128.87, 128.84, 128.09, 127.88, 125.78, 124.76, 124.48, 124.44, 123.10, 122.30, 122.06, 122.03, 121.85, 121.81, 121.10, 116.39, 116.34, 116.01, 115.93, 115.62, 113.73, 113.69, 113.66, 113.61, 68.28, 68.11, 58.63, 58.59, 58.57, 58.53, 58.30, 58.03, 56.17, 56.08, 55.86, 55.77, 55.75, 55.67, 55.58, 48.56, 47.61, 47.53, 40.29, 40.17, 40.13, 30.65, 29.15, 29.06, 28.54, 27.90, 27.71, 25.89, 25.72, 21.21, 20.02. ESI-HRMS m/z calcd for $[M-2I]^{2+}$ 1491.6826; found 1491.6839.

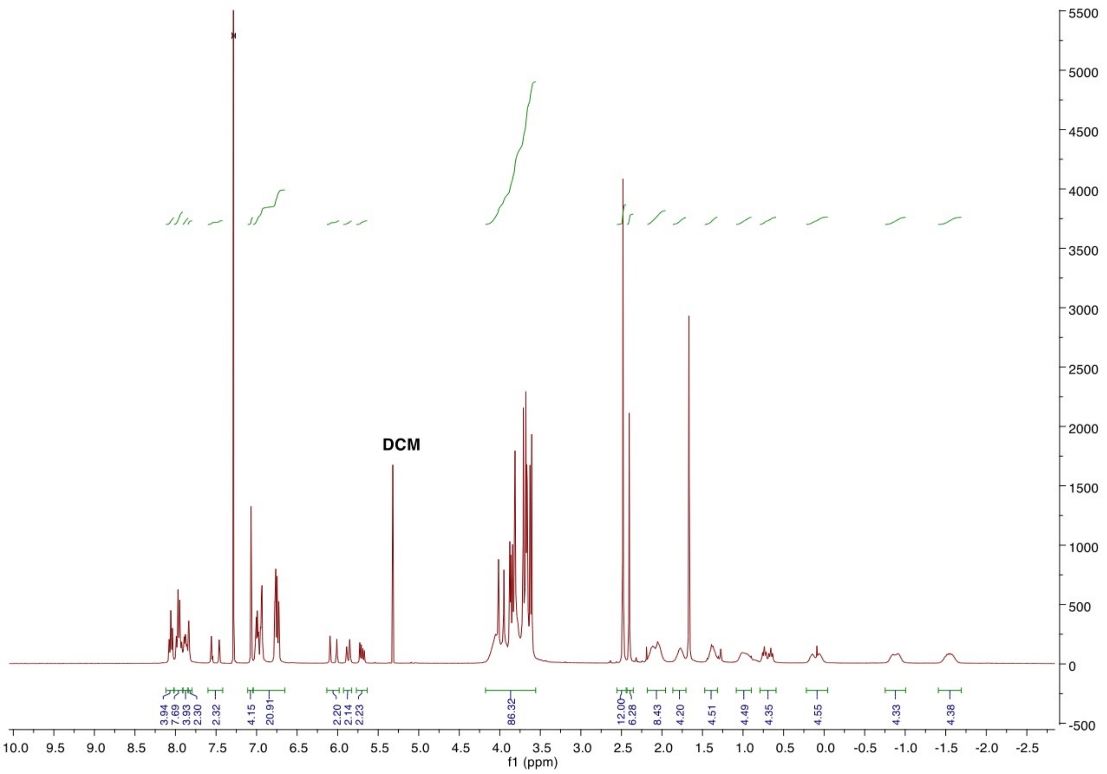

Figure S10. ^{1}H NMR (400 MHz, $CDCl_3$) spectrum of compound **4**.

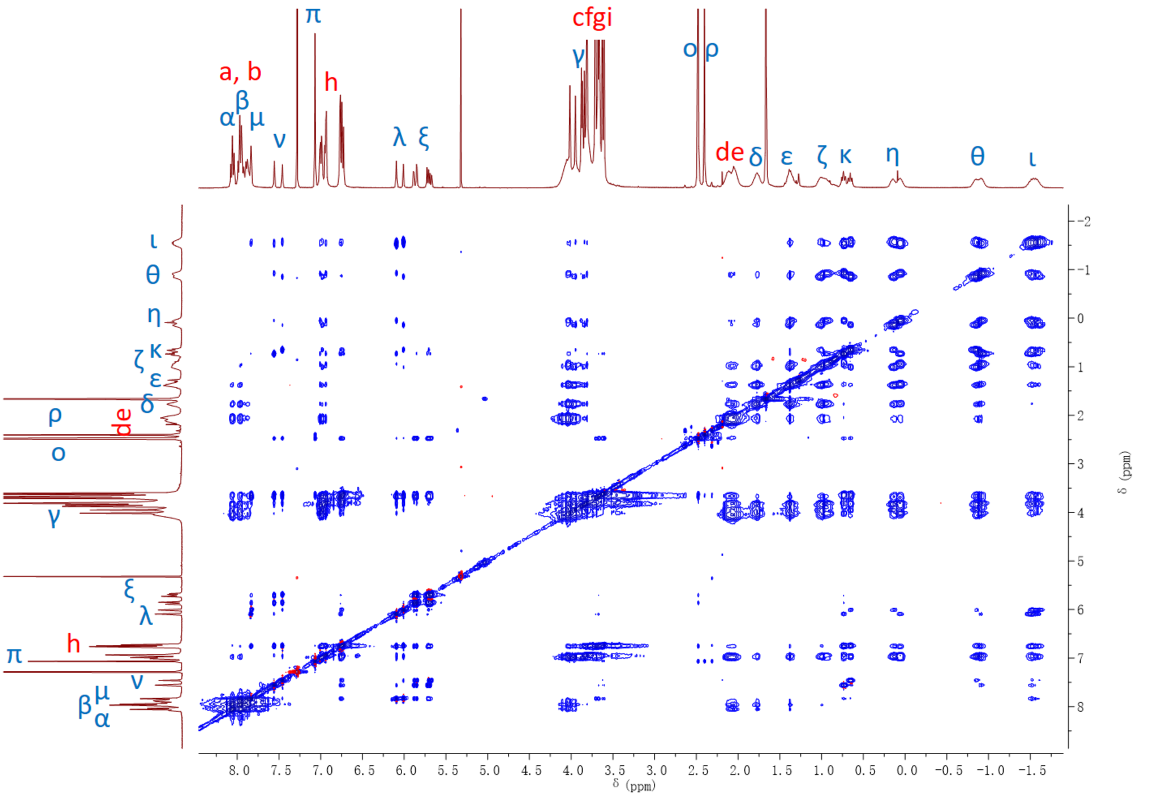

Figure S11. ^{1}H-^{1}H NOESY (400 MHz, $CDCl_3$) spectrum of compound **4**.

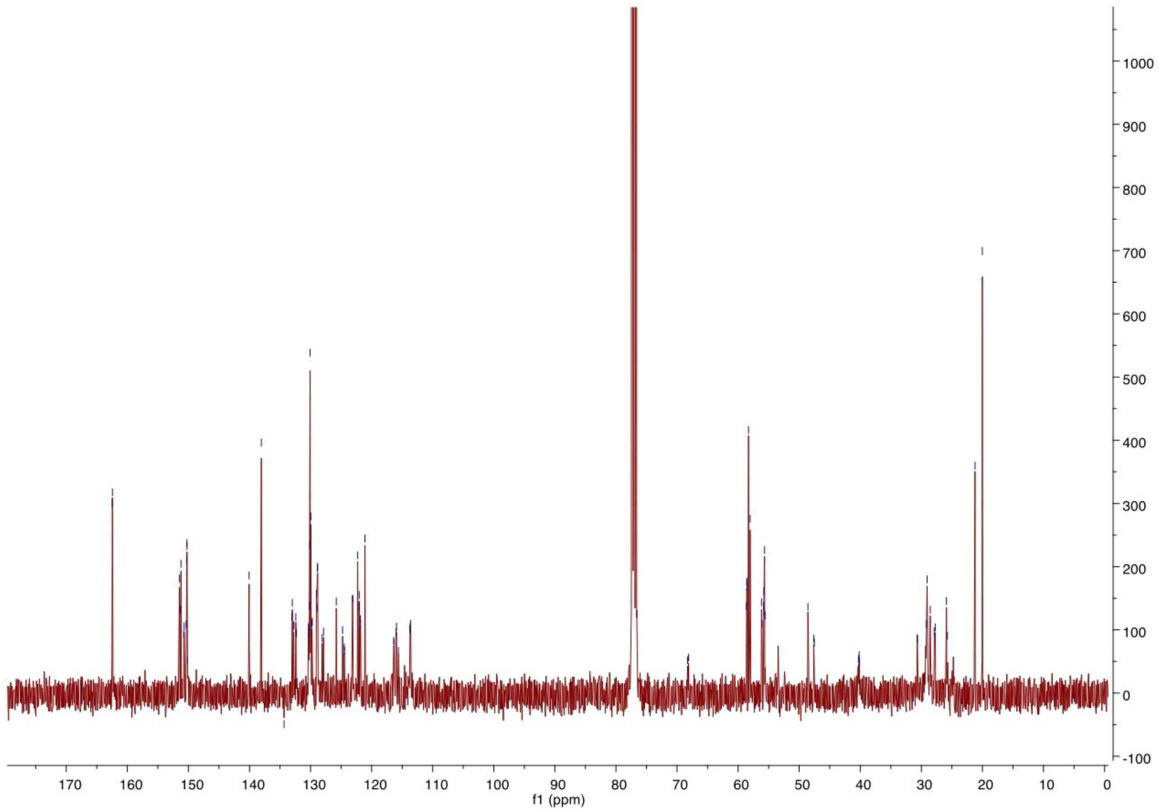

Figure S12. ^{13}C NMR (101 MHz, $CDCl_3$) spectrum of compound **4**.

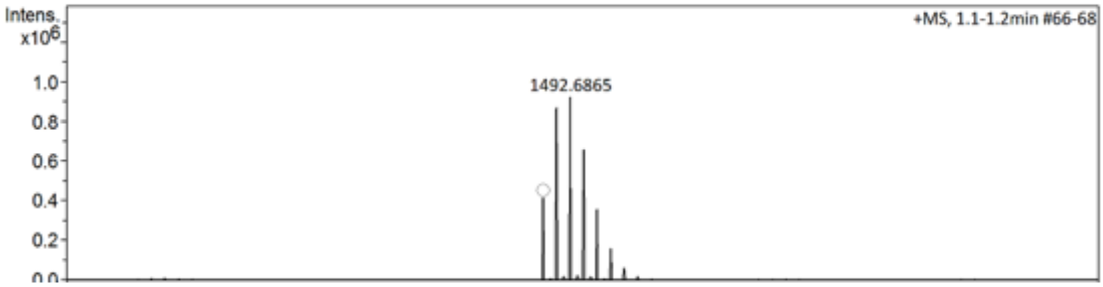

Figure S13. ESI-MS spectrum of compound **4**.

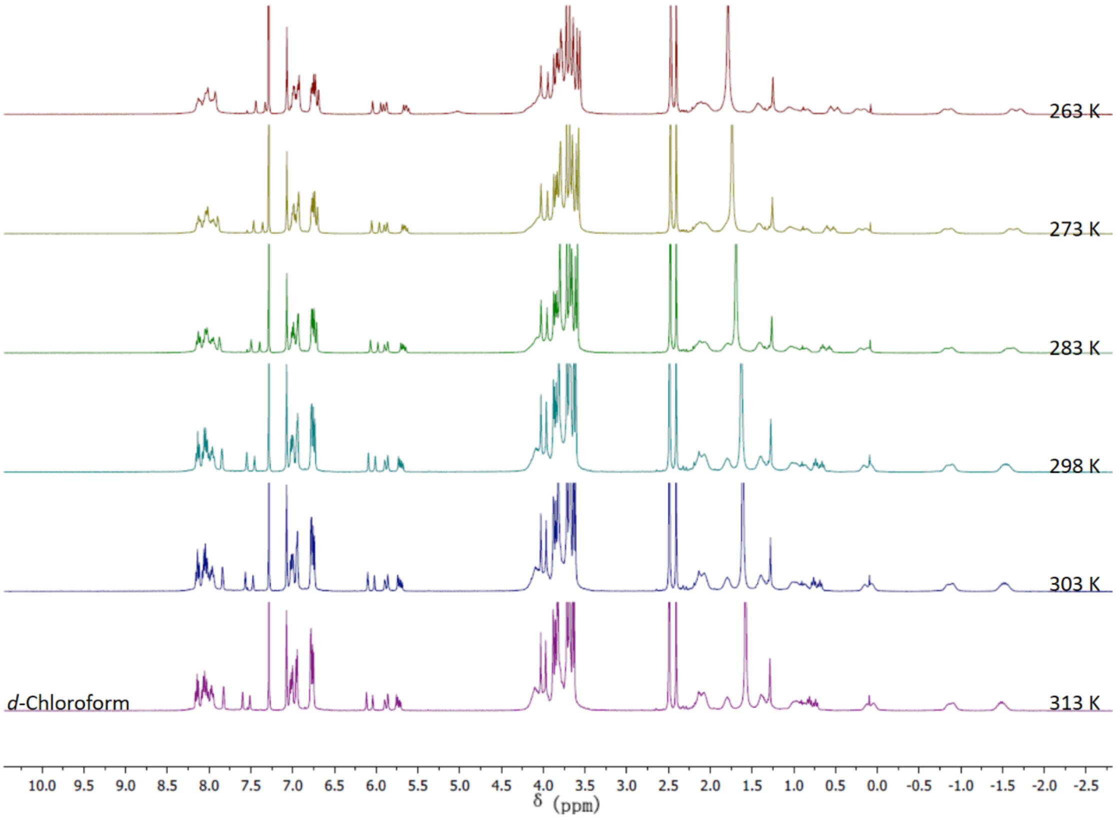

Figure S14. VT ^{1}H NMR (400 MHz, $CDCl_3$) spectrum of compound **4** over the temperature range 263-313K. Note that there are no significant changes to the spectrum over this temperature range.

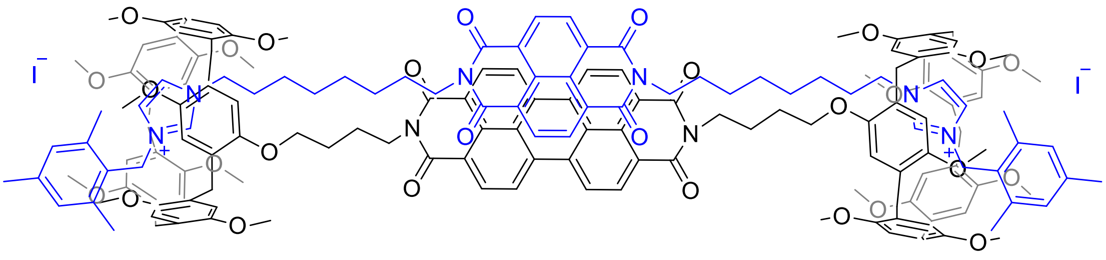

Synthesis of compound **5**: compound **1** (30 mg, 0.015 mmol) and compound **3** (10 mg, 0.016 mmol) were dissolved in 0.4 ml dry $CHCl_3$ under dinitrogen and the mixture cooled to -10°C using an ice/salt bath. 2,4,6-trimethylbenzyl iodide (16 mg, 0.062 mmol) was added to the solution in the absence of light. The reaction mixture was slowly warmed to room temperature and stirred for 3 days. The crude product was purified by size exclusive chromatography using a $CHCl_3$ eluent and then by column chromatography on silica gel with $CHCl_3$/MeOH=10:1. Compound **5** was obtained as red solid (41 mg, yield 89%). ^1H NMR (400 MHz, $CDCl_3$) δ ppm 8.31 (dd, *J* = 7.6, 4.4 Hz, 4H), 8.02 (d, *J* = 8.3 Hz, 4H), 7.96 - 7.78 (m, 6H), 7.50/7.40 (s, 2H), 7.06 (s, 4H), 7.02 - 6.56 (m, 20H), 6.05/5.95 (s, 2H), 5.88 (dd, *J* = 15.0, 6.6 Hz, 2H), 5.70 (dd, *J* = 15.0, 9.9 Hz, 2H), 4.40 (br, 4H), 4.21 - 3.39 (m, 82H), 2.48 (s, 12H), 2.40 (s, 6H), 2.33 - 2.19 (m, 8H), 1.52 - 1.16 (m, 8H), 0.92 - 0.84 (m, 4H), 0.76 - 0.47 (m, 4H), 0.23 - 0.00 (m, 4H), – 0.72 – – 1.02 (m, 4H), – 1.48 - – 1.75 (m, 4H). ^{13}C NMR (101 MHz, $CDCl_3$) δ ppm 162.86, 162.77, 161.42, 151.48, 151.40, 151.28, 151.25, 151.10, 151.02, 150.84, 150.38, 150.34, 150.28, 150.24, 140.05, 140.02, 138.08, 132.49, 132.47, 132.29, 132.23, 130.70, 130.13, 130.07, 130.02, 129.98, 129.74, 129.16, 129.12, 129.11, 129.03, 129.00, 128.80, 128.75, 128.02, 125.83, 125.76, 125.22, 125.01, 124.44, 123.41, 122.66, 122.01, 121.83, 121.02, 116.50, 116.27, 115.66, 115.59, 114.69, 114.38, 113.91, 113.77, 113.63, 113.50, 113.45, 68.31, 58.69, 58.61, 58.55, 58.46, 58.41, 58.30, 58.24, 58.21, 58.01, 56.25, 56.09, 55.80, 55.70, 55.59, 48.69, 48.62, 47.47, 47.32, 40.56, 40.52, 40.38, 30.20, 30.09, 29.21, 29.15, 29.01, 28.79, 28.69, 27.77, 25.63, 25.53, 21.21, 20.07. ESI-HRMS m/z calcd for $[M-2I]^{2+}$ 1429.6683; found 1429.6723.

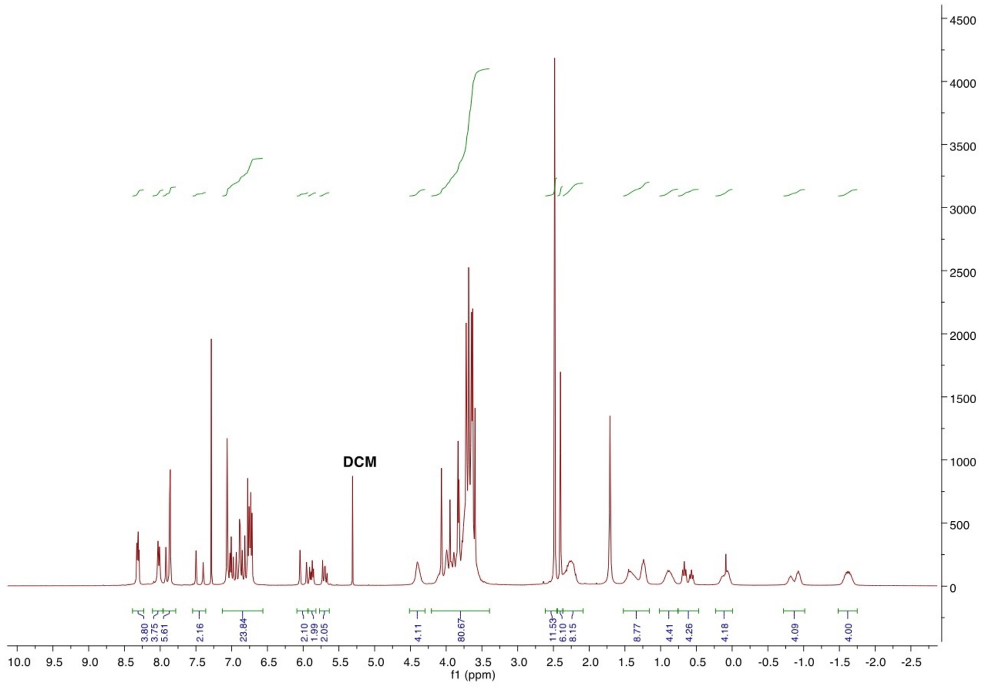

Figure S15. ^1H NMR (400 MHz, $CDCl_3$) spectrum of compound **5**.

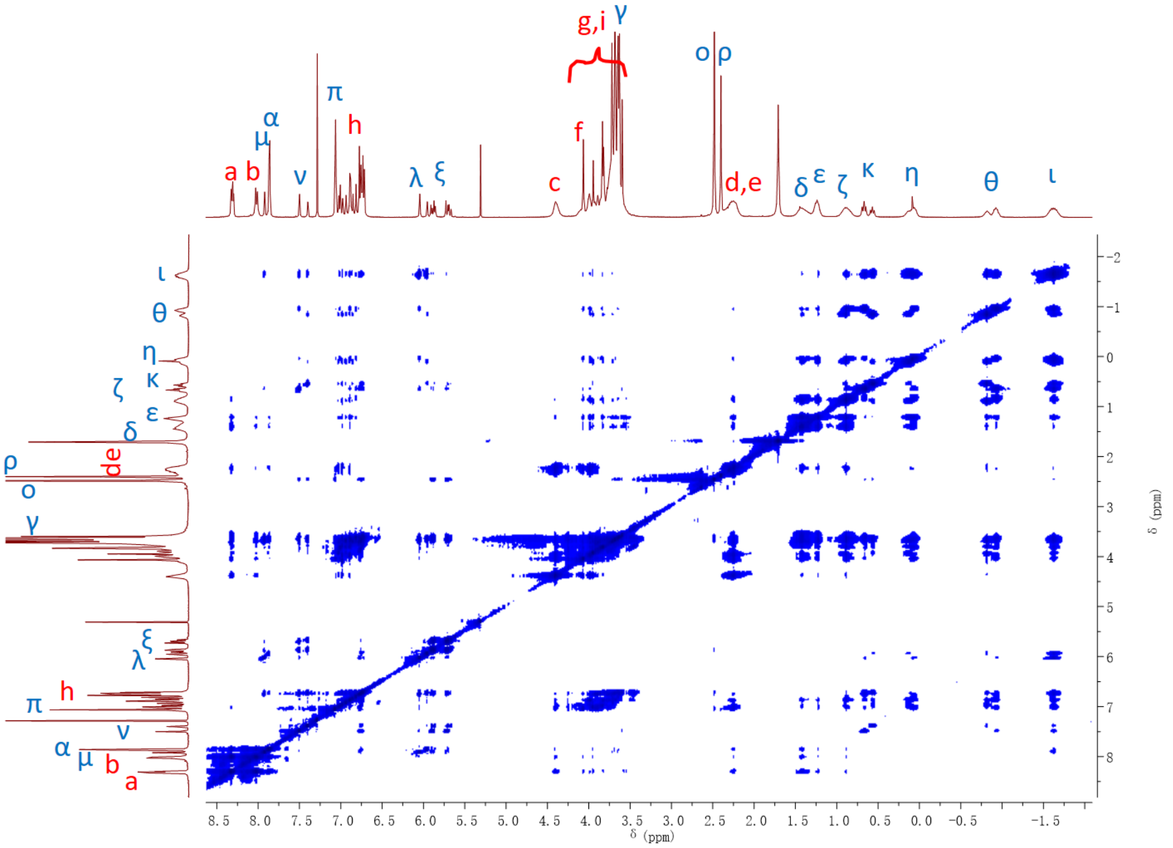

Figure S16. ^{1}H-^{1}H NOESY (400 MHz, $CDCl_3$) spectrum of compound **5**.

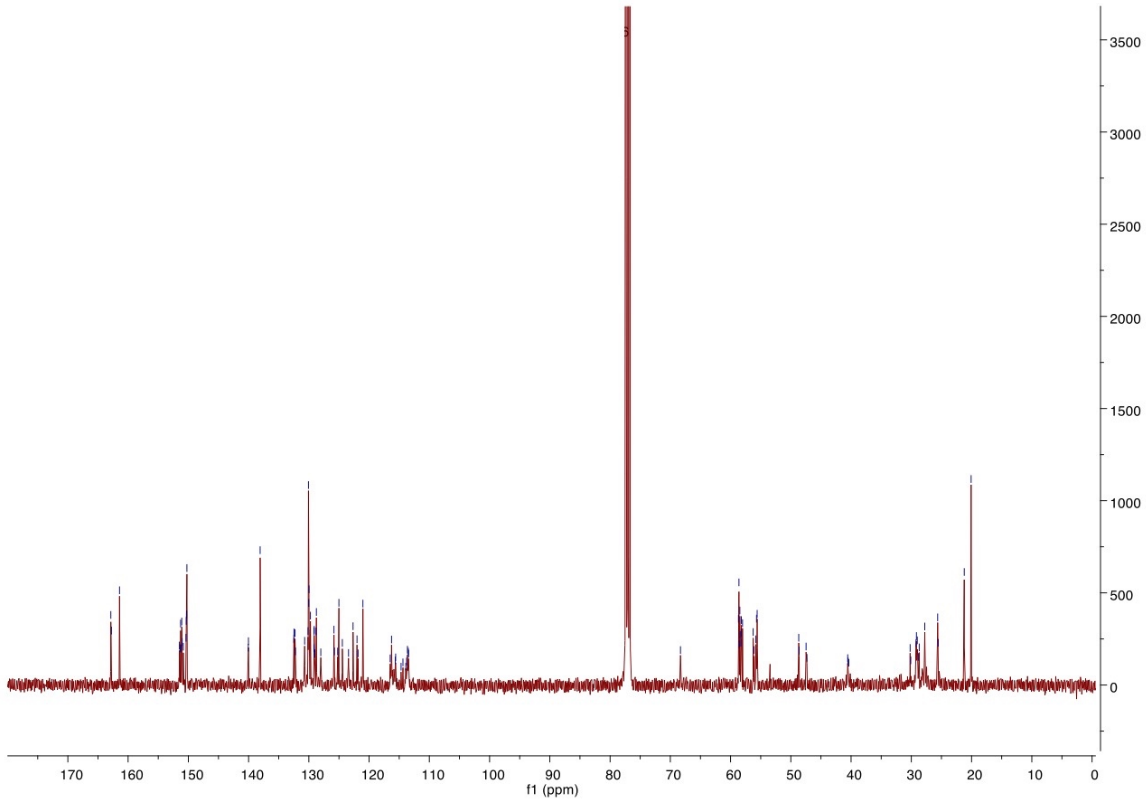

Figure S17. ^{13}C NMR (101 MHz, $CDCl_3$) spectrum of compound **5**.

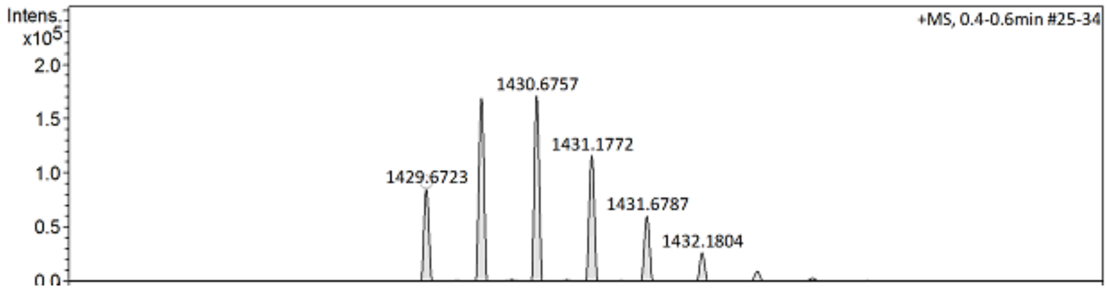

Figure S18. ESI-MS spectrum of compound **5**.

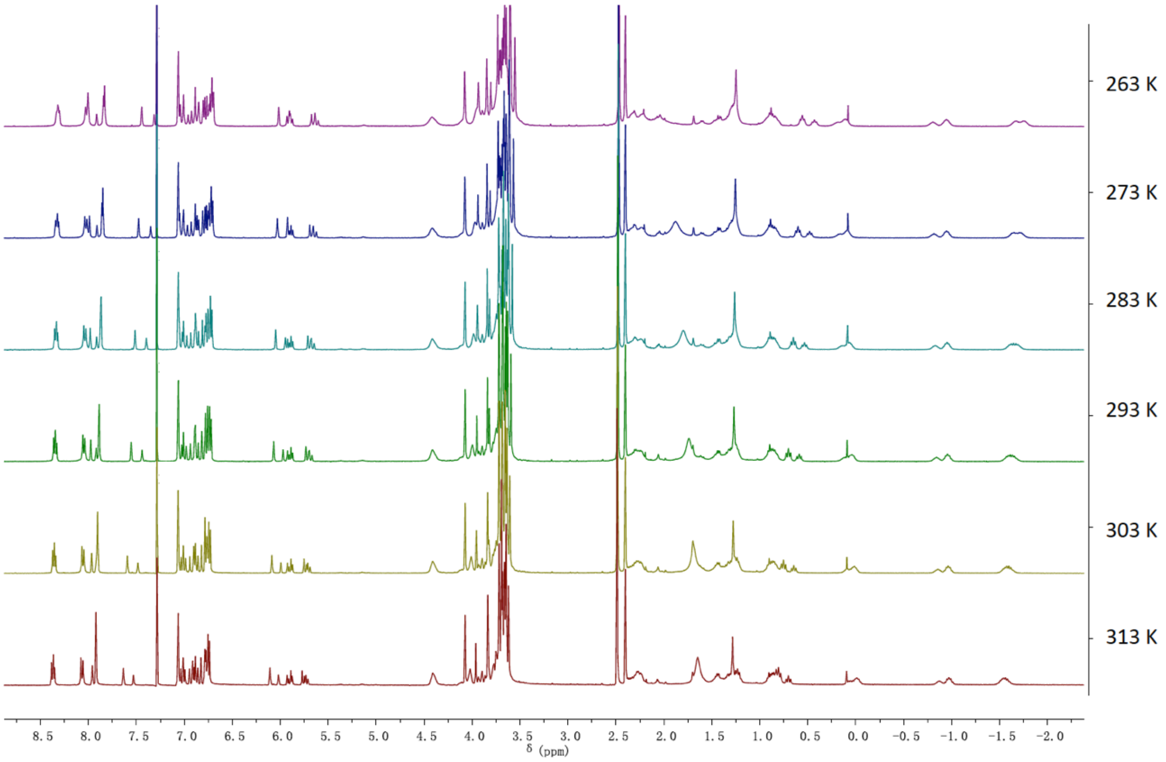

Figure S19. VT ^{1}H NMR (400 MHz, $CDCl_3$) spectrum of compound **5** over the temperature range 263-313K. As with VT NMR spectra of **4** there are no significant changes to the spectrum over this temperature range.

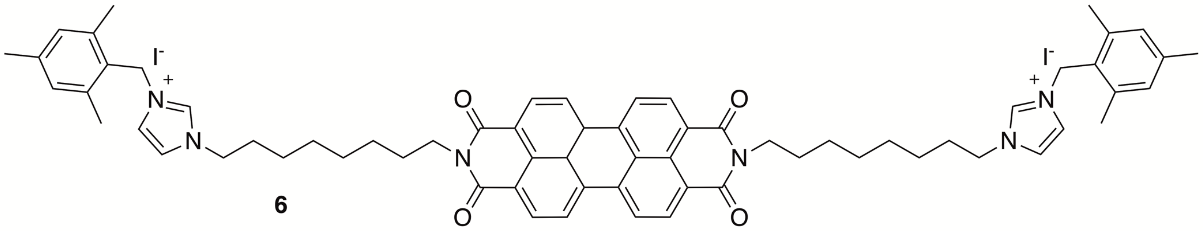

Synthesis of compound **6**: Compound **2** (30 mg, 0.040 mmol) and 2,4,6-trimethylbenzyl iodide (31 mg, 0.12 mmol) were dissolved in 5 ml of $CHCl_3$ under a dinitrogen atmosphere and in the absence of light. The mixture was stirred overnight. The solvent was removed *in vacuo*. The crude product was purified

by column chromatography on silica gel (CH_2Cl_2:MeOH=20:1). The product, compound **6**, was obtained as a red solid (19 mg, yield 38%). The compound forms an insoluble aggregate quickly in $CHCl_3$, therefore MeOH was added to prevent aggregation. ^{1}H NMR (400 MHz, $CDCl_3$/MeOD) δ ppm 9.32 (s, 2H), 8.16 (d, *J* = 7.9 Hz, 4H), 8.07 (d, *J* = 8.2 Hz, 4H), 7.45 (d, *J* = 1.9 Hz, 2H), 6.98 (d, *J* = 1.9 Hz, 2H), 6.86 (s, 4H), 5.43 (s, 4H), 4.25 (t, *J* = 7.5 Hz, 4H), 3.95 (t, *J* = 7.4 Hz, 4H), 2.22 (s, 12H), 2.19 (s, 6H), 1.93 - 1.81 (m, 4H), 1.68 - 1.56 (m, 4H), 1.33 (br, 16H). ^{13}C NMR (126 MHz, $CDCl_3$/MeOD) δ ppm 163.32, 140.06, 137.83, 134.38 (2C, one from imidazolium and one from PDI core), 131.22, 129.82, 128.86, 125.94, 124.85, 123.29, 122.68, 122.38, 121.64, 49.94, 47.78, 40.22, 29.80, 28.60, 28.32, 27.50, 26.54, 25.77, 20.65, 19.32. ESI-HRMS m/z calcd for $[M-2I]^{2+}$ 506.2802; found 506.2808.

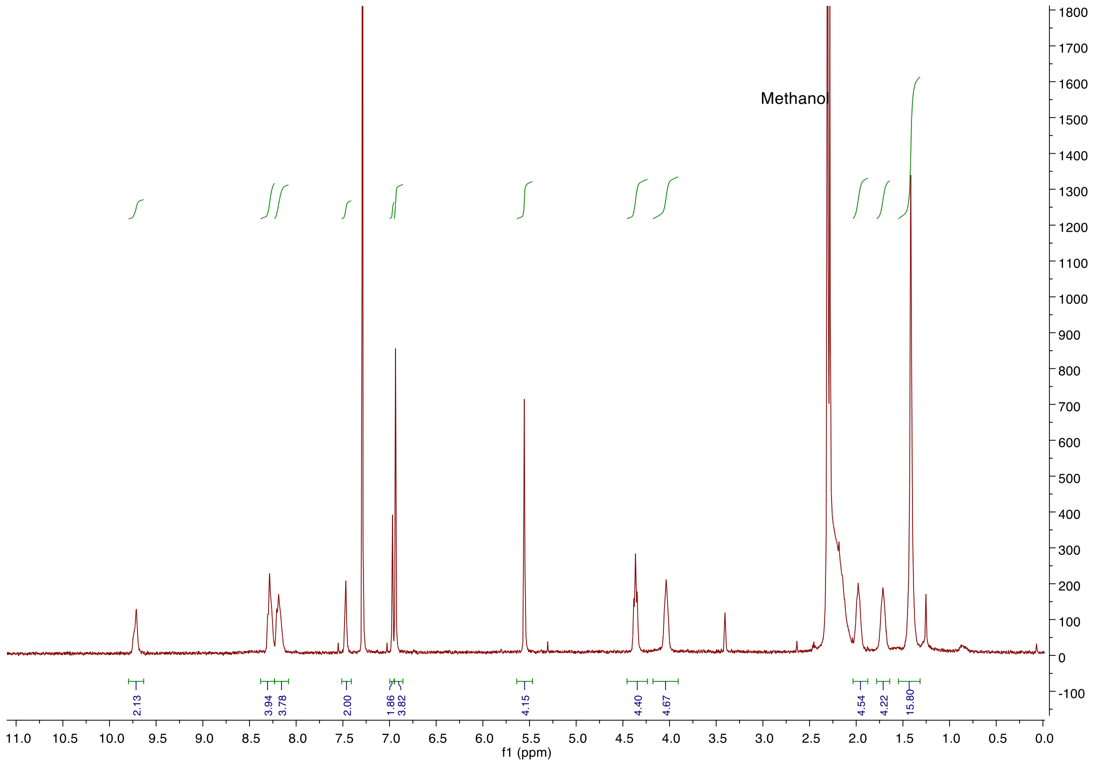

Figure S20. ^{1}H NMR (400 MHz, $CDCl_3$/MeOD) spectrum of compound **6** immediately taken after preparation.

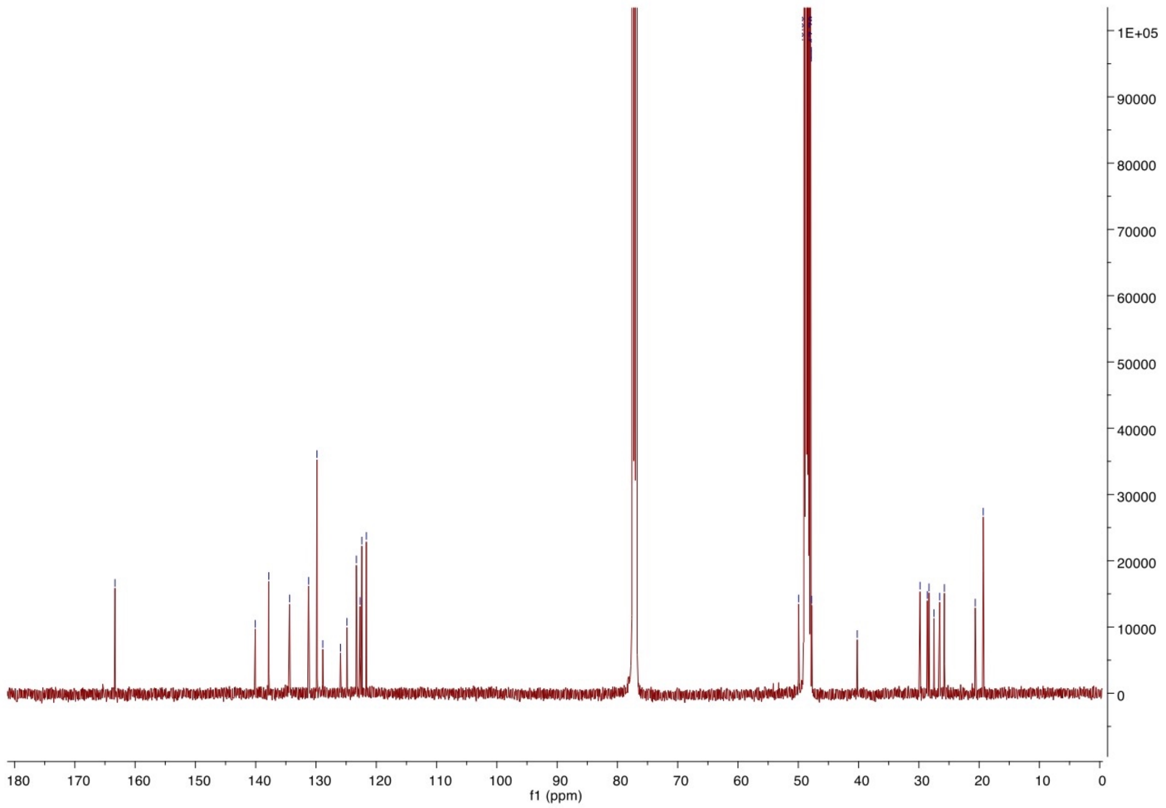

Figure S21. ^{13}C NMR (126 MHz, $CDCl_3$/MeOD) spectrum of compound **6**.

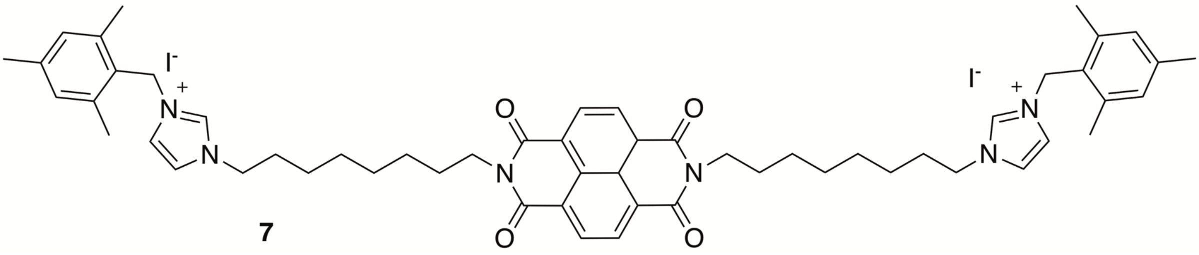

7

Synthesis of compound **7**: Compound **3** (20 mg, 0.032 mmol) and 2,4,6-trimethylbenzyl iodide (25 mg, 0.096 mmol) were dissolved in 3 ml of $CHCl_3$ under a dinitrogen atmosphere and in the absence of light. The mixture was stirred overnight. The solvent was removed *in vacuo*. The crude product was purified by column chromatography on silica gel (CH_2Cl_2:MeOH=10:1). The product, compound **11**, was obtained as brown solid (24 mg, yield 70%). ^{1}H NMR (400 MHz, $CDCl_3$) δ ppm 10.05 (s, 2H), 8.73 (s, 4H), 7.50 (s, 2H), 6.96 (s, 2H), 6.92 (s, 4H), 5.58 (s, 4H), 4.36 (t, *J* = 7.4 Hz, 4H), 4.17 (t, *J* = 7.3 Hz, 4H), 2.29 (s, 12H), 2.28 (s, 6H), 2.01 - 1.91 (m, 4H), 1.77 - 1.69 (m, 4H), 1.36 (br, 16H). ^{13}C NMR (101 MHz, $CDCl_3$) δ ppm 162.82, 140.04, 138.14, 136.07, 131.00, 130.00, 126.66, 126.54, 125.18, 122.30, 120.98, 50.33, 48.19, 40.67, 30.12, 28.88, 28.60, 27.81, 26.67, 26.02, 21.07, 20.09. ESI-HRMS m/z calcd for $[M-2I]^{2+}$ 444.2646; found 444.2659.

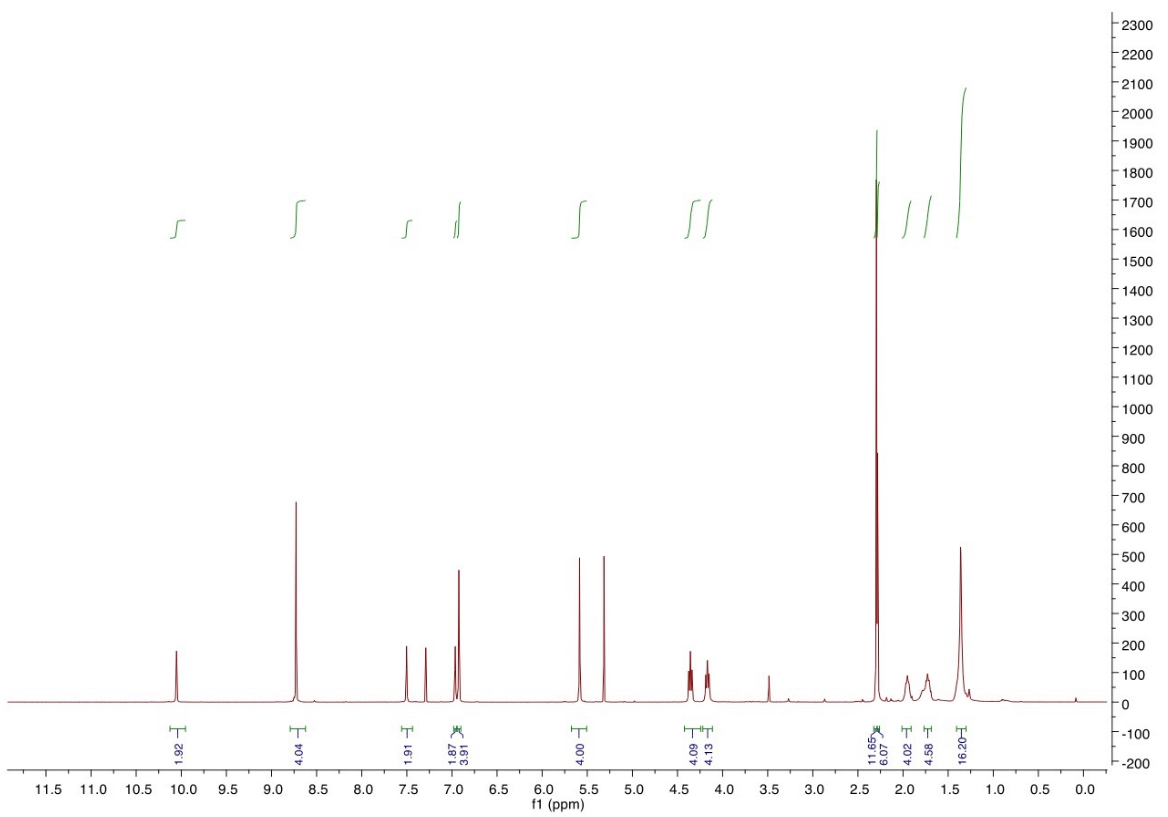

Figure S22. ^{1}H NMR (400 MHz, $CDCl_3$) spectrum of compound **7**.

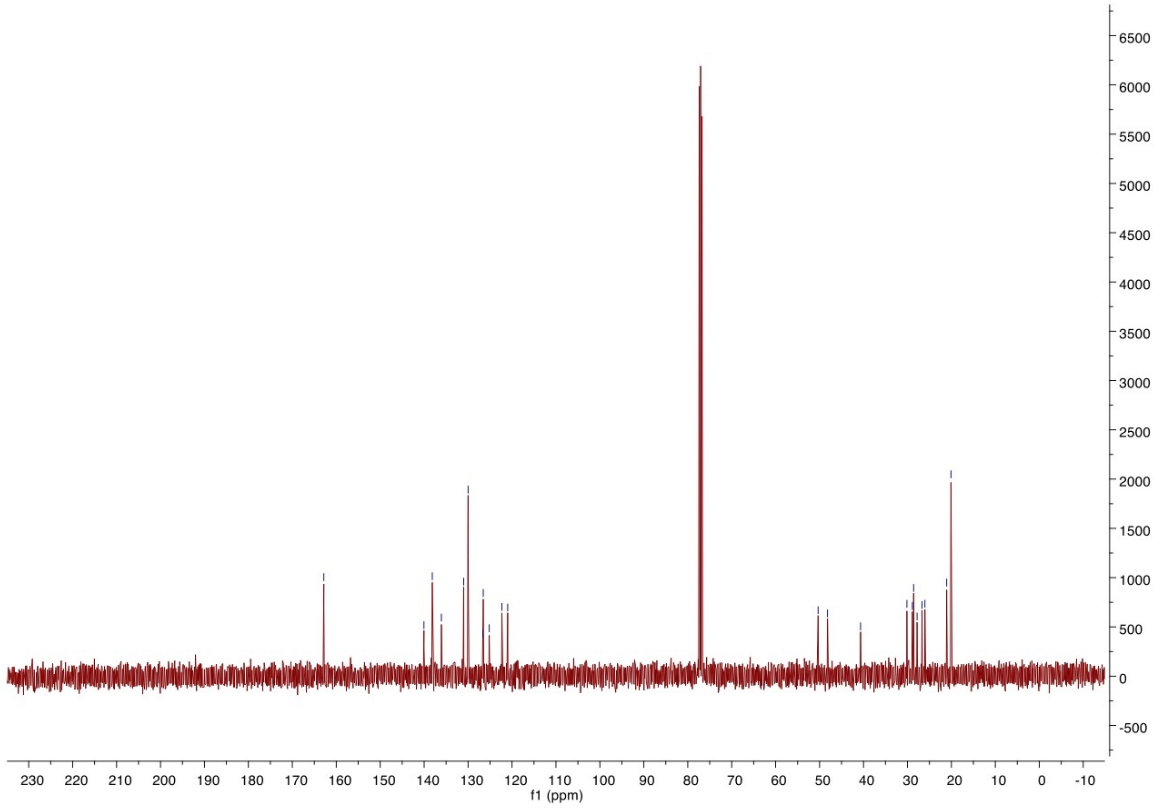

Figure S23. ^{13}C NMR (101 MHz, $CDCl_3$) spectrum of compound **7**.

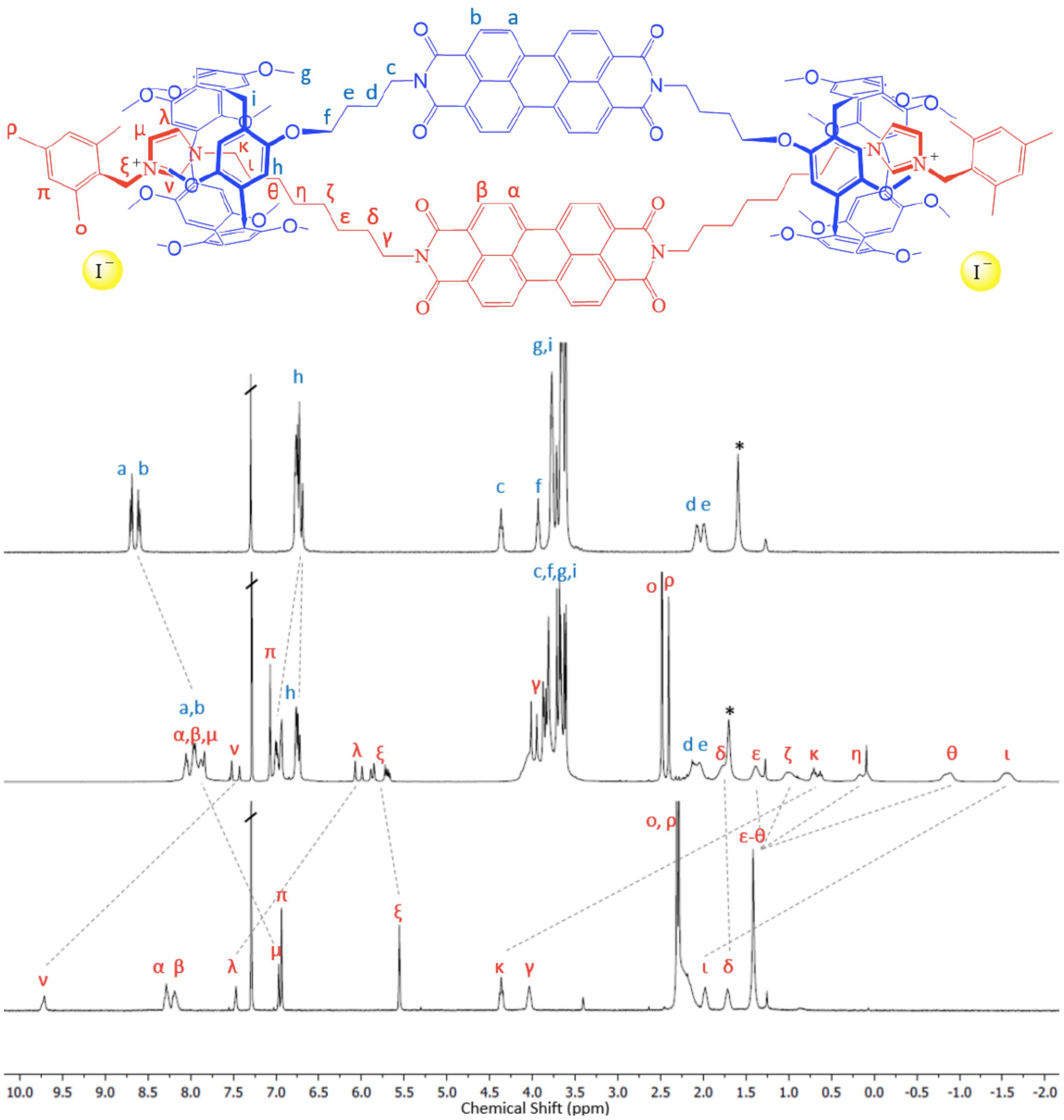

Figure S24. ^{1}H NMR spectra for compounds **1** (top); **4** (middle); **6** (bottom).

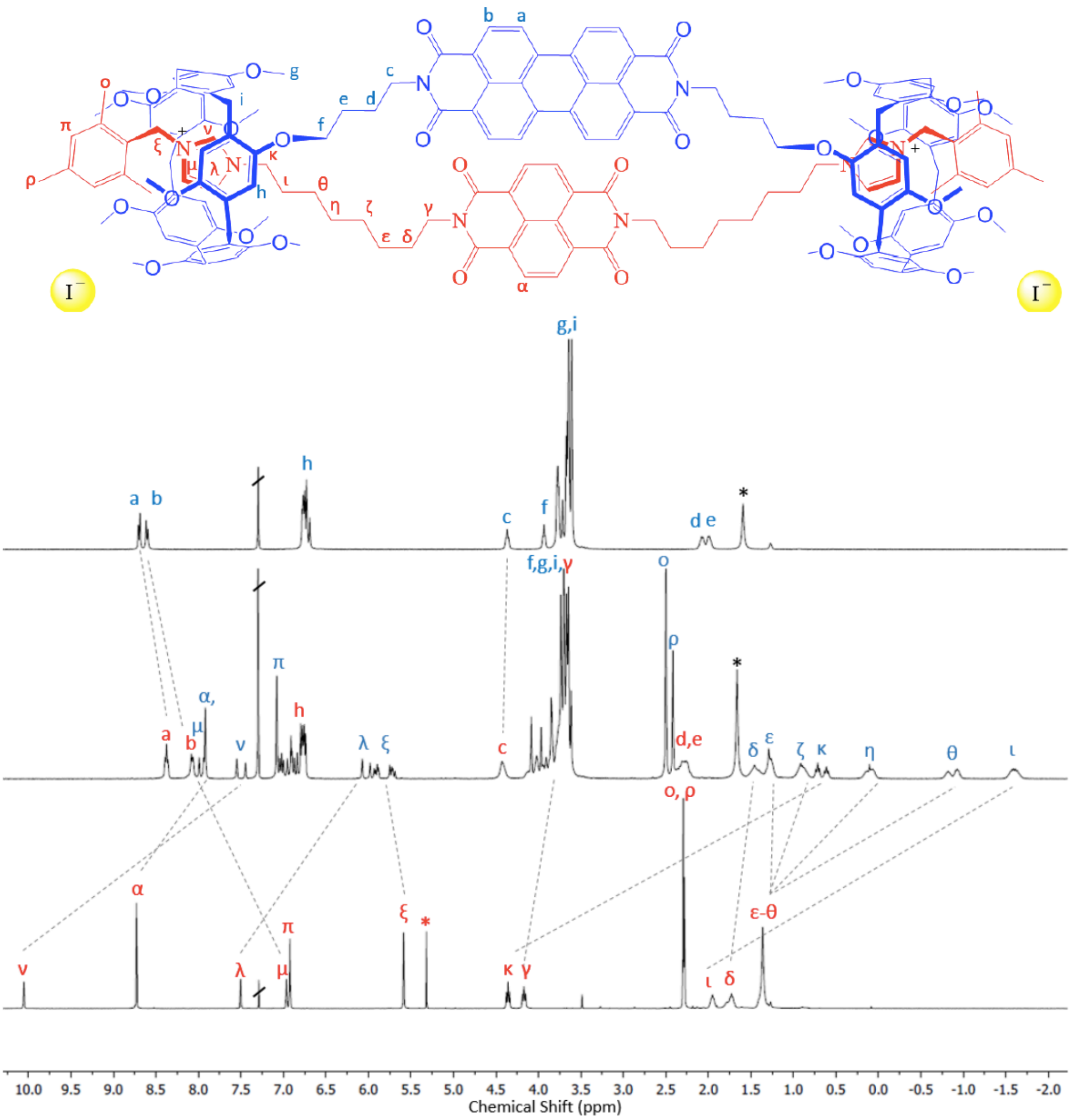

Figure S25. ^{1}H NMR spectra for compounds **1** (top); **5** (middle); **7** (bottom).

Electrochemical and Spectroelectrochemical Measurements

Materials: All chemical reagents were used as-purchased from Alfa Aesar, Fisher Scientific, Sigma-Aldrich, or VWR International, unless stated otherwise. Anhydrous toluene was dried by passing through a column packed with 4 Å molecular sieves, degassed and stored over a potassium mirror in a nitrogen atmosphere. Anhydrous dichloromethane was purchased from Sigma-Aldrich (Fluka) and stored over 4 Å molecular sieves. Ferrocene was purchased from Aldrich and used as received. $[^nBu_4N][BF_4]$ was prepared using literature methods.[S4] Column chromatography was performed on Merck silica gel 60 (0.2-0.5 mm, 50 - 130 mesh).

Experimental: Standard UV/vis spectra were collected on a Perkin Elmer Lambda 16 spectrophotometer using a 1cm pathlength quartz cuvette. Fluorescence spectra were recorded as aerated solutions using a Jobin Yvon Horiba FluoroMax-3 spectrometer at ambient temperature in a 1cm pathlength quartz cuvette. Quantum yields were calculated by comparison with the fluorescence observed for perylene orange (Φ = 0.99 in $CHCl_3$) for PDI containing compounds and quinine sulfate (Φ = 0.53 in 0.05 M H_2SO_4) for NDI containing **7** under identical conditions of irradiation.[S5] NIR data were recorded on either an Agilent Cary 5000 spectrometer using a 1 cm pathlength quartz cuvette or a Bruker Vertex 80 spectrometer using a 1 mm pathlength solution IR cell fitted with CaF_2 windows. FT-IR data were recorded on a Nicolet Avatar 360 spectrometer using a 1 mm pathlength solution IR cell fitted with KBr windows. X-band EPR spectroscopic data were recorded on a Bruker EMX spectrometer as fluid (ambient temperature) or frozen (77 K) solutions in quartz tubes. Spectra were simulated using WINEPR SimFonia software.

Cyclic and square wave voltammetry experiments were carried out using either an Autolab PGSTAT20 or an Autolab PGSTAT302N potentiostat under an argon atmosphere using a three-electrode arrangement in a single compartment cell. A glassy carbon working electrode, a platinum wire secondary electrode and a saturated calomel reference electrode were used in the cell. The reference electrode was chemically isolated from the test solution using a bridge tube terminated with a vycor fritt and containing electrolyte solution. An analyte concentration of 1 mM was used with $[^nBu_4N][BF_4]$ (0.4 M) as a supporting electrolyte. Redox potentials are referenced to the ferrocenium/ferrocene couple, however decamethylferrocene, using the $[(\eta^5\text{-}C_5Me_5)_2Fe]^+/[(\eta^5\text{-}C_5Me_5)_2Fe]$ couple, was employed as the internal standard.[S6] Under these circumstances, the redox processes of the test compounds were referenced to the Fc^+/Fc couple by an independent calibration. The separation between the $[(\eta^5\text{-}C_5Me_5)_2Fe]^+/[(\eta^5\text{-}C_5Me_5)_2Fe]$ couple and the Fc^+/Fc couple recorded under identical conditions was 0.52 V. No compensation was applied for internal resistance.

UV/vis spectroelectrochemical measurements were performed using an optically transparent electrochemical cell, consisting of a modified quartz cuvette with a 0.5 mm path length. A three-electrode configuration of a platinum/rhodium gauze working electrode, platinum wire secondary electrode and a saturated calomel reference electrode (chemically isolated as above) were used in the cell. The potential at the working electrode was controlled with a Sycopel Scientific Ltd DD10M potentiostat. Temperature control was achieved by passing a stream of chilled nitrogen across the surface of the cell. $[^nBu_4N][BF_4]$ (0.4 M) was used as the supporting electrolyte for the experiments. Concentrations of analyte were *ca.* 0.5 mM in CH_2Cl_2 containing 0.4 M $[^nBu_4N][BF_4]$.

Bulk electrolysis was performed to generate samples for study by FT-IR, NIR and EPR spectroscopies. Samples were prepared under an argon atmosphere at 0 °C in a two-component cell: a platinum/rhodium gauze working electrode and secondary electrode were separated by a glass frit. A saturated calomel reference electrode was bridged to the test solution through a vycor frit, oriented

at the centre of the working electrode. The working electrode compartment, containing analyte (1 mM), was stirred rapidly with a magnetic stirrer bar during electrolysis. [$^nBu_4N$][BF_4] (0.4 M) was used as the supporting electrolyte for the experiments. After electrolysis was completed, the prepared solution was transferred by Teflon cannula for analysis.

For comparison to studies of the handcuffs the cyclic voltammograms of a 1:1 mixture of **1** and **6** were used as a reference for our electrochemical investigations of **4**. The 1:1 mixture of **1** and **6** gave a response consistent with that of the sum of the individual components, that is, two reduction processes separated by *ca.* 0.2 V, precluding intra-molecular interactions arising from radical anion dimerization as the source of CV response in **4** – a result not unexpected given the low association constant for such a process.[S7]

Table S1. Absorption and Emission data for compounds **1**, **4**-**7**.[a]

		Absorption (λ_{abs} / nm)					Emission (λ_{em} / nm)			(υ_{abs} - υ_{em}) / cm^{-1}	$\lambda_{Excitation}$ / nm	Φ
		NDI bands		PDI bands								
**1**	$CHCl_3$	-	-	458	489	**525**	**533**	574	-	286	485	0.002
**4**	$CHCl_3$	-	-	-	**493**	533	**622**	-	-	2685	533	-
	CH_2Cl_2	-	-	-	**492**	531	**627**	-	-	2883	490	0.016
	MeCN	-	-	-	**489**	528	**655**	-	-	3672	529	-
	THF	-	-	-	**491**	530	**625**	-	-	2868	490	-
**5**	$CHCl_3$	364	385	464	495	**532**	**543**	585	-	381	486	-
	CH_2Cl_2	365	384	463	494	**532**	**544**	585	-	415	490	0.004
	MeCN	363	383	466	494	**530**	**532**	574	624	71	490	-
**6**	$CHCl_3$	-	-	458	489	**525**	**534**	575	624	321	490	0.800
	CH_2Cl_2	-	-	459	489	**525**	**534**	576	624	321	486	-
**7**	$CHCl_3$	360	**381**	-	-	-	**385**	408	-	273	360	0.001

[a] Bold indicates the most intense band

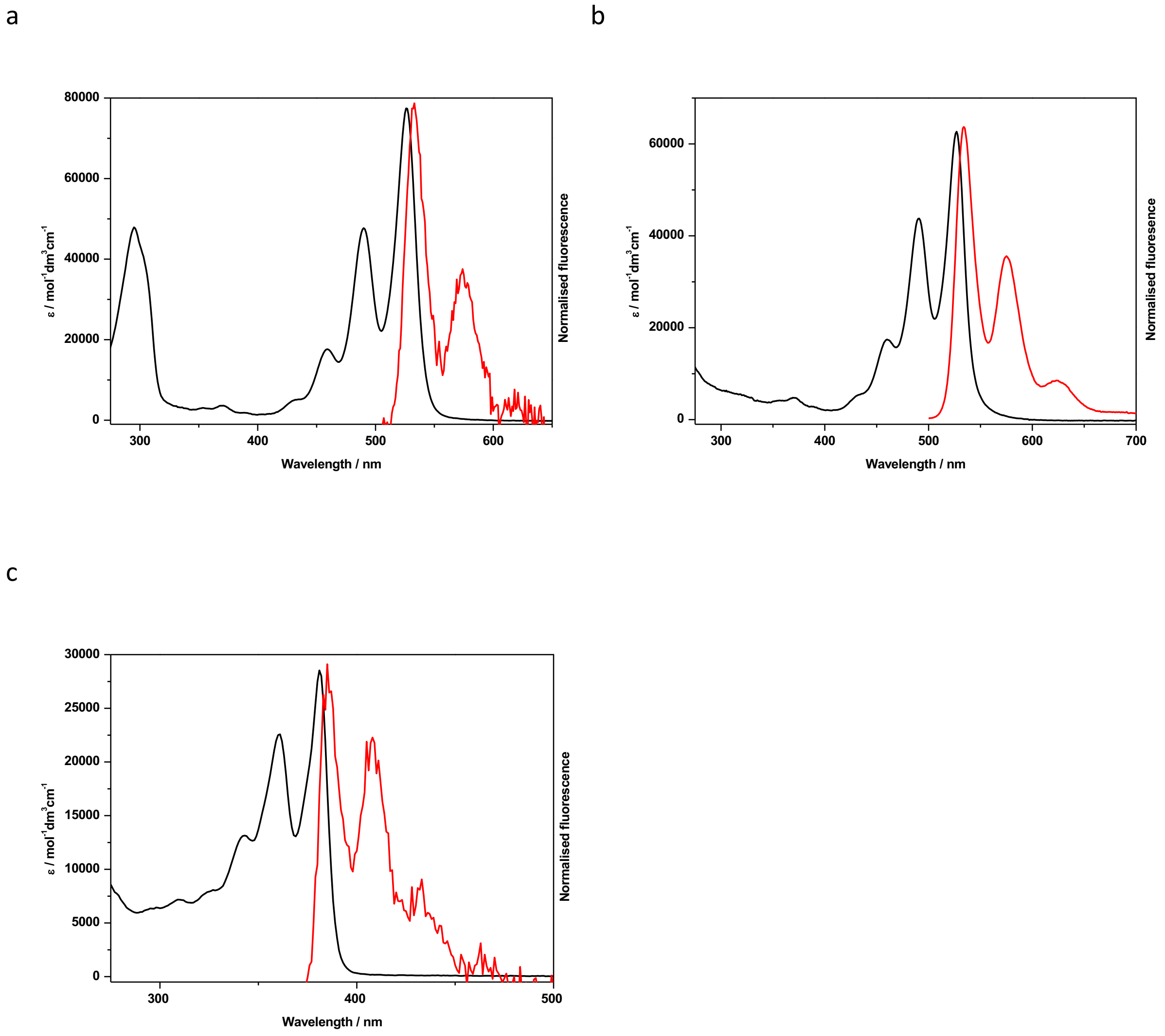

Figure S26. Absorption (black trace) and emission (red trace) spectra of: (a) **1** in $CHCl_3$; (b) **6** in $CHCl_3$; (b) **7** in $CHCl_3$.

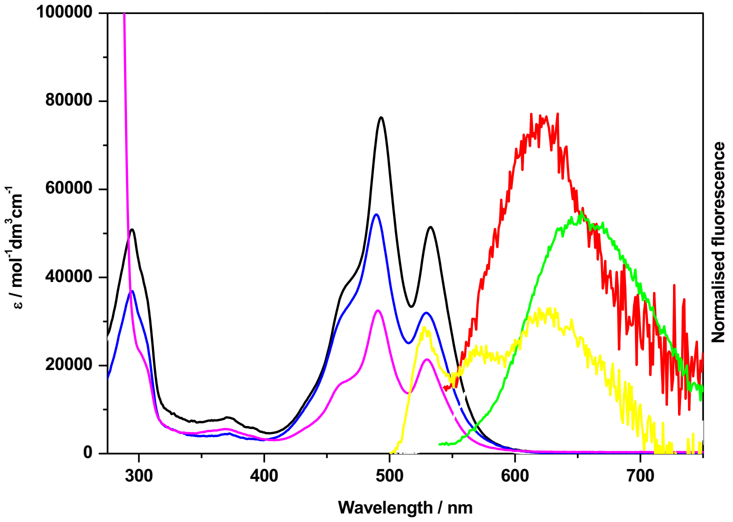

Figure S27. Absorption (black, blue and magenta traces) and emission (red, green and yellow traces) spectra of **4** in $CHCl_3$, CH_3CN and THF, respectively.

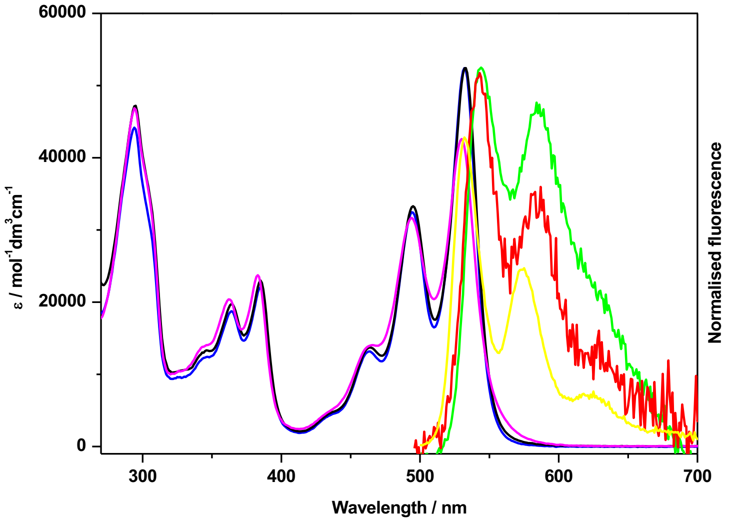

Figure S28. Absorption (black, blue and magenta traces) and emission (red, green and yellow traces) spectra of **5** in $CHCl_3$, CH_2Cl_2 and CH_3CN, respectively.

Table S2. UV/vis Spectroscopic data (230-900 nm) for compounds **1**, **4**-**7**.[a]

	UV/vis λ_{abs} / nm (ε x 10^{-3} / $mol^{-1}dm^3cm^{-1}$)				
Compound	Neutral	Monoanion	Dianion	Trianion	Tetraanion
**1**	260 (31.9), 295 (51.5), 371 (3.7), 460 (15.6), 491 (42.6), 528 (68.6)	295 (66.8), 350 (4.6), 529 (3.7), 680 (37.1), 713 (78.4), 766 (18.6), 796 (42.7)	292 (91.7), 389 (2.7), 568 (71.0), 635 (18.6)	-	-
**4**	294 (5.6), 494 (7.9), 534 (5.2)	293 (6.4), 495 (3.2), 534 (5.6), 719 (3.7), 803 (2.4)	292 (7.6), 399 (0.8), 615 (7.2)	-	278 (11.9), 291 (13.3), 533 (9.2), 565 (11.6), 634 (3.1)
**5**	294 (7.9), 365 (2.8), 386 (3.4), 466 (2.0), 497 (4.7), 535 (7.4)	294 (8.5), 364 (2.5), 384 (2.6), 498 (2.0), 535 (3.5), 714 (3.0), 798 (1.9)[b]	289 (10.3), 473 (2.5), 612 (4.6)	278 (12.8), 292 (15.0), 476 (5.7), 532 (6.0), 569 (8.9), 589 (6.9), 635 (3.0), 751 (0.9)	265 (10.7), 292 (15.6), 399 (4.7), 423 (6.3), 569 (10.7), 591 (8.8), 636 (2.5)
		293 (9.4), 364 (2.2), 381 (2.3), 714 (7.6), 770 (2.4)[c]			
**6**	260 (24.2), 292 (6.1), 370 (4.4), 459 (13.4), 491 (34.9), 527 (51.2)	236 (66.2), 276 (22.1), 352 (3.6), 382 (3.0), 635 (15.4), 680 (25.6), 713 (50.4), 767 (12.6), 797 (26.8)	266 (31.0), 278 (41.0), 289 (40.8), 387 (2.4), 567 (56.0), 632 (15.7)	-	-
**7**	240 (41.4), 309 (6.1), 342 (10.6), 360 (18.4), 381 (23.7)	271 (18.0), 332 (4.8), 381 (2.1), 404 (2.5), 479 (26.5), 612 (8.4), 673 (3.4), 745 (5.9)	242 (42.7), 399 (20.3), 423 (29.4), 507 (3.5), 546 (9.3), 594 (16.2)	-	-

[a]In CH_2Cl_2 containing [$^nBu_4N$][BF_4] (0.4 M) at 273 K; [b] reduction at point 'A' figure 3 gives these bands; [c] reduction at point 'B' Figure 3 gives these bands.

a b

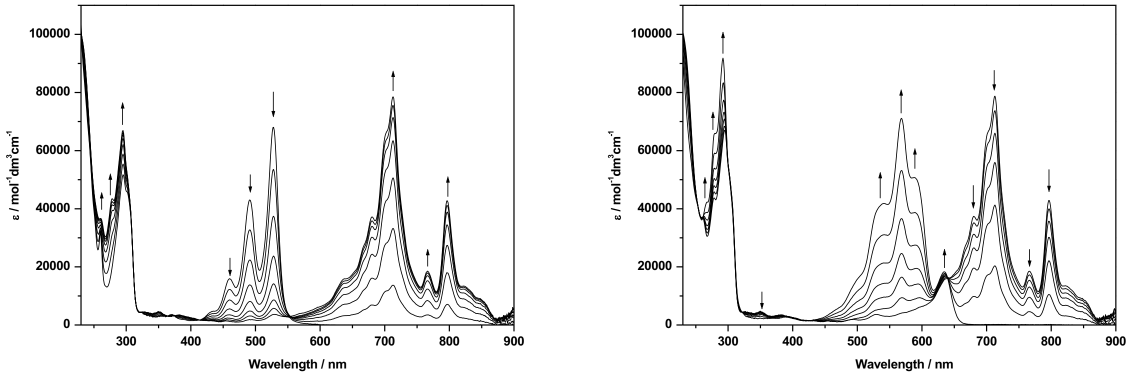

Figure S29. UV-vis absorption spectra showing the inter-conversion between (a) **1** and [**1**]$^{-}$ and (b) [**1**]$^{-}$ and [**1**]$^{2-}$. Arrows indicate the progress of reduction. Spectra were recorded in CH_2Cl_2 containing [$^{n}Bu_4N$][BF_4] (0.4 M) as the supporting electrolyte at 273 K.

a b

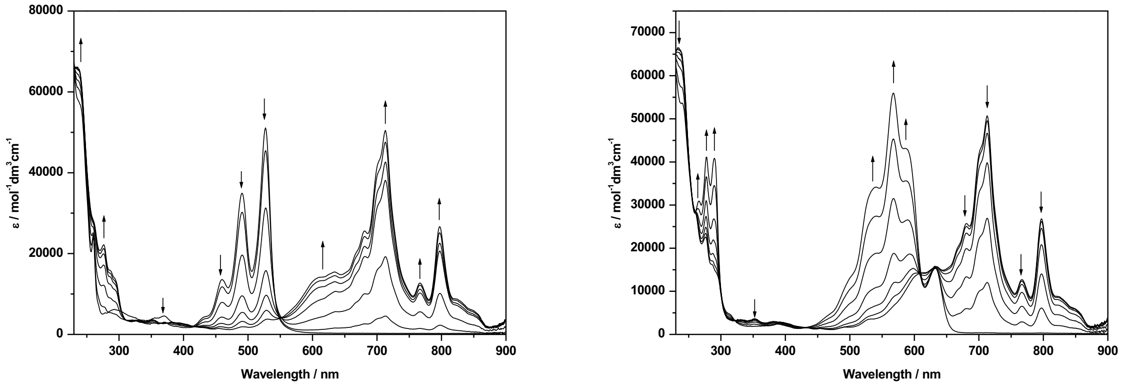

Figure S30. UV-vis absorption spectra showing the inter-conversion between (a) **6** and [**6**]$^{-}$ and (b) [**6**]$^{-}$ and [**6**]$^{2-}$. Arrows indicate the progress of reduction. Spectra were recorded in CH_2Cl_2 containing [$^{n}Bu_4N$][BF_4] (0.4 M) as the supporting electrolyte at 273 K.

a b

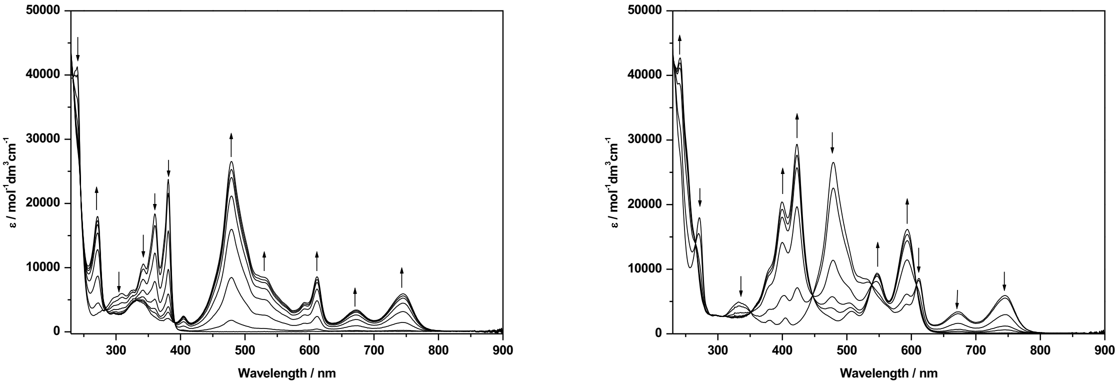

Figure S31. UV-vis absorption spectra showing the inter-conversion between (a) **7** and [**7**]$^-$ and (b) [**7**]$^-$ and [**7**]$^{2-}$. Arrows indicate the progress of reduction. Spectra were recorded in CH_2Cl_2 containing [$^nBu_4N$][BF_4] (0.4 M) as the supporting electrolyte at 273 K.

a b

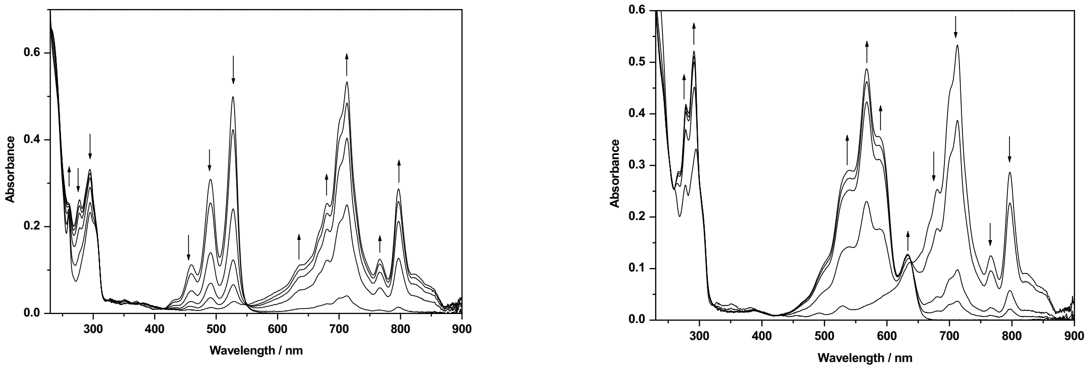

Figure S32. UV-vis absorption spectra recorded for a mixture of **1** and **6** showing the inter-conversion between (a) **1** and [**1**]$^-$, **6** and [**6**]$^-$; (b) [**1**]$^-$ and [**1**]$^{2-}$, [**6**]$^-$ and [**6**]$^{2-}$. Arrows indicate the progress of reduction. Spectra were recorded in CH_2Cl_2 containing [$^nBu_4N$][BF_4] (0.4 M) as the supporting electrolyte at 273 K. The spectra of a mixture of **1** and **6** show no indication of intermolecular π-stacking.

a

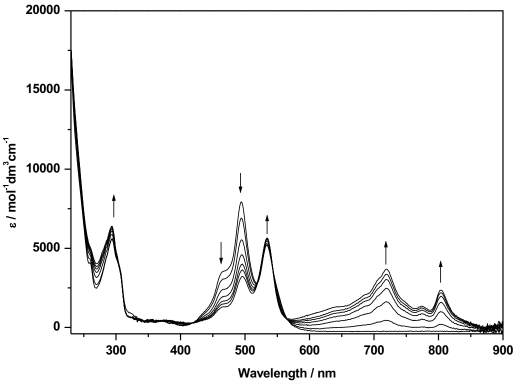

b

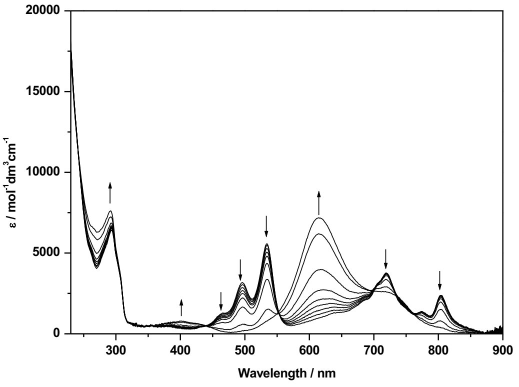

c

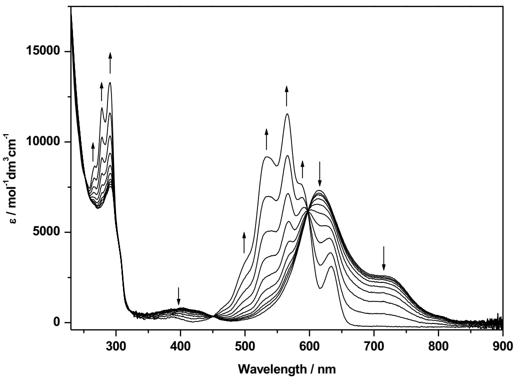

Figure S33. UV-vis absorption spectra showing the inter-conversion between (a) **4** and [**4**]$^{-}$; (b) [**4**]$^{-}$ and [**4**]$^{2-}$; (c) [**4**]$^{2-}$ and [**4**]$^{4-}$. Arrows indicate the progress of reduction. Spectra were recorded in CH_2Cl_2 containing [$^{n}Bu_4N$][BF_4] (0.4 M) as the supporting electrolyte at 273 K.

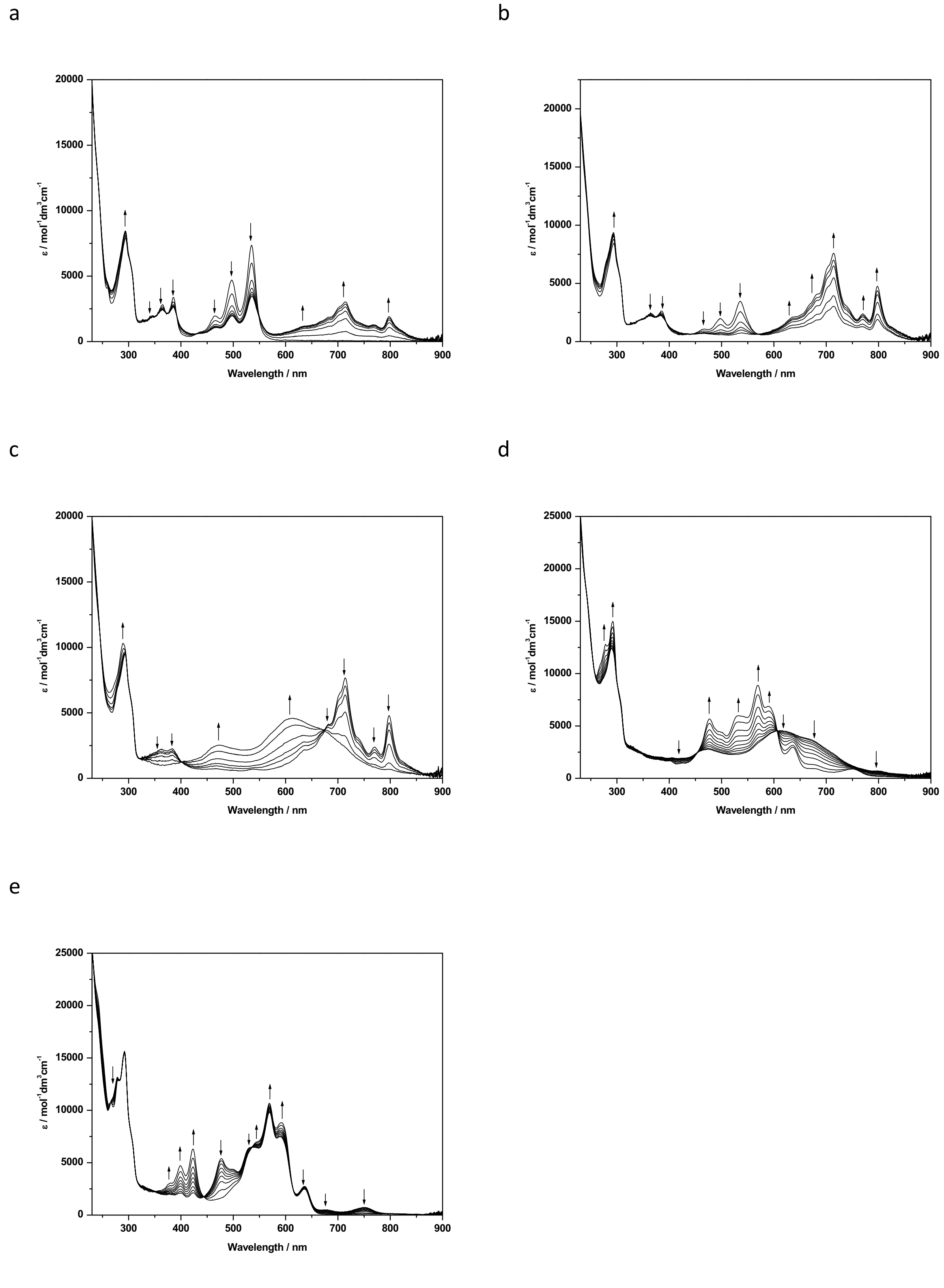

Figure S34. UV-vis absorption spectra showing the inter-conversion between (a) **5** and $[\mathbf{5}]^{1/2-}$; (b) $[\mathbf{5}]^{1/2-}$ and $[\mathbf{5}]^{-}$; (c) $[\mathbf{5}]^{-}$ and $[\mathbf{5}]^{2-}$; (d) $[\mathbf{5}]^{2-}$ and $[\mathbf{5}]^{3-}$; (e) $[\mathbf{5}]^{3-}$ and $[\mathbf{5}]^{4-}$. Arrows indicate the progress of reduction. Spectra were recorded in CH_2Cl_2 containing $[^{n}Bu_4N][BF_4]$ (0.4 M) as the supporting electrolyte at 273 K.

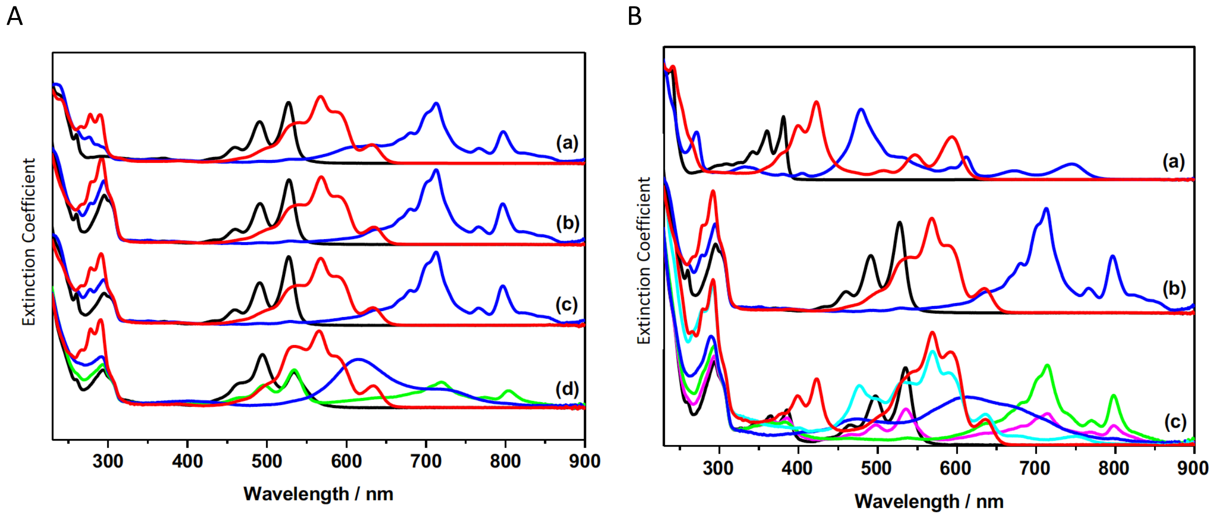

Figure S35. Comparison of spectral profiles for: (A)(a) **6** (black trace), [**6**]$^-$ (blue trace), [**6**]$^{2-}$ (red trace); (b) **1** (black trace), [**1**]$^-$ (blue trace), [**1**]$^{2-}$ (red trace); (c) 1:1 mixture of **1** and **6** (black trace), first reduction (blue trace), second reduction (red trace); (d) **4** (black trace), [**4**]$^-$ (green trace), [**4**]$^{2-}$ (blue trace), [**4**]$^{4-}$ (red trace). (B)(a) **7** (black trace), [**7**]$^-$ (blue trace), [**7**]$^{2-}$ (red trace); (b) **1** (black trace), [**1**]$^-$ (blue trace), [**1**]$^{2-}$ (red trace); (c) **5** (black trace), [**5**]$^{1/2-}$ (magenta trace), [**5**]$^-$ (green trace), [**5**]$^{2-}$ (blue trace), [**5**]$^{3-}$ (cyan trace), [**5**]$^{4-}$ (red trace). Spectra were recorded in dichloromethane containing [^{n}Bu$_4$N][BF$_4$] (0.4 M) as the supporting electrolyte at 273 K. See Table S2 for detailed values of extinction coefficients for each case.

Near- and Mid-IR Spectroscopy

We followed the re-oxidation of [**4**]$^{2-}$ to [**4**]$^-$ by NIR spectroscopy. Figure S32 shows the loss of the broad band at 1527 nm, associated with [**4**]$^{2-}$, and the formation of new bands at 719, 804 and 972 nm, all of which are consistent with the generation of an anionic PDI, the bands for which can extend to 1100 nm dependent upon substituent.[S8] We find no evidence of an NIR IVCT band for [**4**]$^-$ in this region. The absence of an intervalent charge transfer band in the NIR region for the one electron reduced pimer was unexpected. It is noted that the transition energy of a radical anion dimer (1527 nm, 6549 cm^{-1} for [**4**]$^{2-}$) is generally about twice that for the one-electron reduced pimer[S9] hence the IVCT band for the pimer may either not exist or occur in the MIR.

IR spectra of neutral and mono-reduced **1** and **6** and [**4**]$^-$ and [**4**]$^{2-}$ were recorded in the range between 4000 and 1500 cm^{-1}; experimental bands to lower energy and those between 2860-3120 cm^{-1} are obscured by CH_2Cl_2 and/or [^{n}Bu$_4$N][BF$_4$] electrolyte bands. In this region the IR spectra of **1**, **6** and **4** are very similar and are characterised by three distinct bands. The bands at *ca.* 1695 and 1657 cm^{-1} are assigned as the symmetric and asymmetric vibrations of the imide carbonyls, respectively. The band at 1596 cm^{-1} is assigned to a wag vibration of C-H on the perylene core.[S10-S13] Reduction of the precursor compounds to their monoanions, [**1**]$^-$ and [**6**]$^-$, depletes bands associated with the neutral species and shifts bands to lower energy (1640, 1585 and 1528 cm^{-1}). Reduction of **4** to [**4**]$^{2-}$ corresponding to one electron per PDI, gives a spectrum with main features very similar to those of [**1**]$^-$ and [**6**]$^-$ ie 1641, 1589 and 1530 cm^{-1}, although bands for [**4**]$^{2-}$ appear broader than those of [**1**]$^-$ and [**6**]$^-$ and additional bands are noted. No feature of the [**4**]$^-$ spectrum could be unequivocally assigned to an IVCT band in this region. Reduction of **4** to [**4**]$^-$ depletes, but does not remove completely, bands associated with its neutral form and develops bands that share characteristics with

$[\mathbf{4}]^{2-}$. Features consistent with both neutral and monoanionic PDIs would appear to suggest a localised configuration on this (faster) timescale, i.e. [PDI]-$[PDI]^{-}$, similar to that noted by UV/vis spectroscopy.

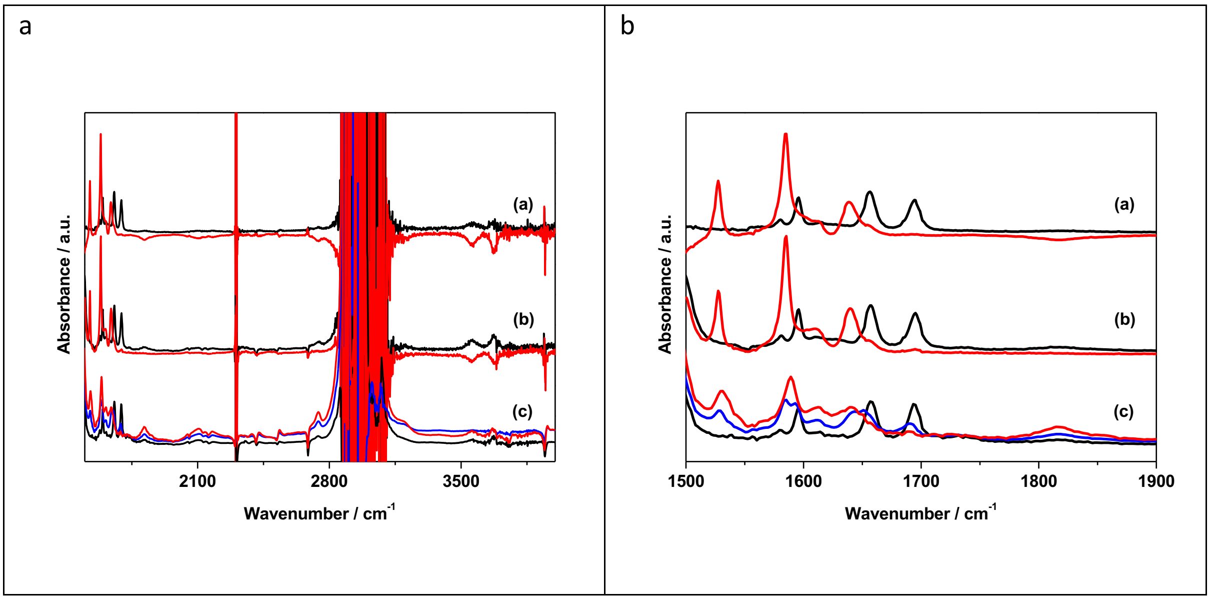

Figure S36. FT-IR spectra of: (a) **6** (black trace) and $[\mathbf{6}]^{-}$ (red trace); (b) **1** (black trace) and $[\mathbf{1}]^{-}$ (red trace); (c) **4** (black trace) $[\mathbf{4}]^{-}$ (blue trace) and $[\mathbf{4}]^{2-}$ (red trace). Solutions of the anions were electrogenerated at 273 K from their parent molecules in CH_2Cl_2 containing $[^nBu_4N][BF_4]$ (0.4 M) as the supporting electrolyte. Spectra were recorded at ambient temperature.

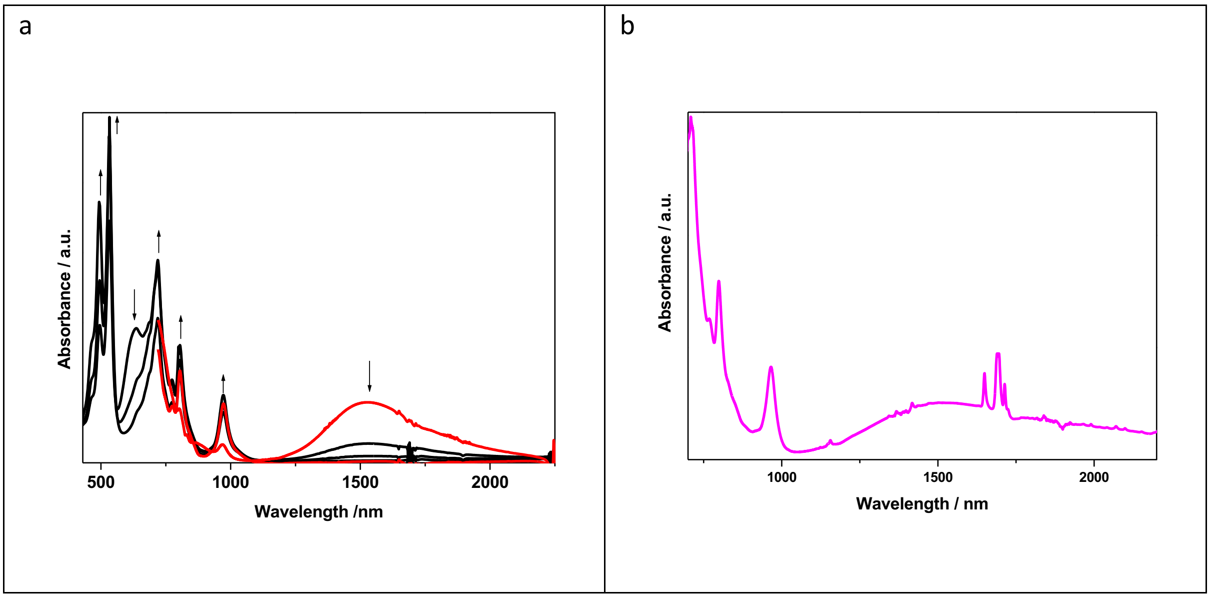

Figure S37. NIR spectra of: (a) the oxidation of $[\mathbf{4}]^{2-}$ to $[\mathbf{4}]^{-}$. Arrows indicate the progress of oxidation. Composite of two experiments with data recorded using a 1 cm pathlength quartz cuvette (black traces) and a 1 mm pathlength solution IR cell with CaF_2 windows (red traces) ; (b) $[\mathbf{5}]^{2-}$ (purple band). Spectra were recorded in CH_2Cl_2 containing $[^nBu_4N][BF_4]$ (0.4 M) as the supporting electrolyte at ambient temperature.

Table S3. FT-IR Spectroscopic data[a] (1500-1900 cm^{-1}) for compounds **1, 4** and **6**.

Compound	ν(CO) sym / cm^{-1}	ν(CO)asym / cm^{-1}	ν(CH-wag) / cm^{-1}	Anion	Band / cm^{-1}
**1**	1695	1657	1596	$[\mathbf{1}]^-$	1640, 1585, 1528
**4**	1694	1657	1595	$[\mathbf{4}]^-$	1690, 1650, 1644, 1611, 1592, 1585, 1528
				$[\mathbf{4}]^{2-}$	1818, 1640, 1612, 1589, 1530
**6**	1696	1656	1596	$[\mathbf{6}]^-$	1639, 1585, 1527

[a]In CH_2Cl_2 containing $[^nBu_4N][BF_4]$ (0.4 M) at ambient temperature.

Further UV-visible Spectra

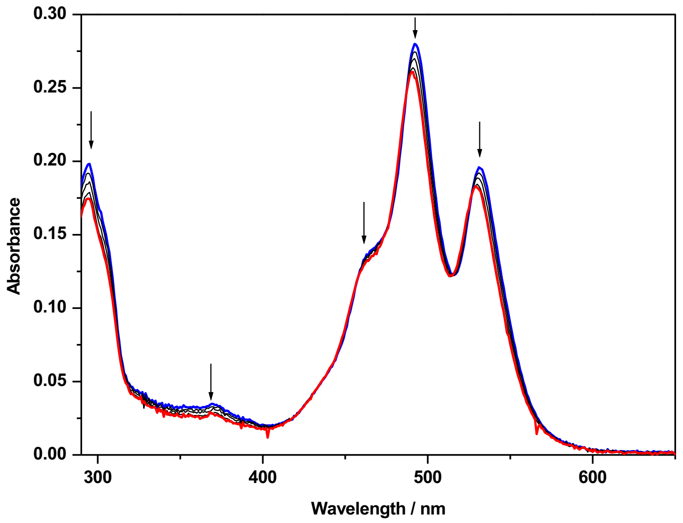

Figure S38. UV/vis spectra of **4** in $CHCl_3$ at 295 (blue line), 303, 313, 323 and 338 (red line) K. Arrows indicate the effect of increasing temperature. The change was reversed on cooling. There is a general decrease in absorbance and a small blue shift on warming.

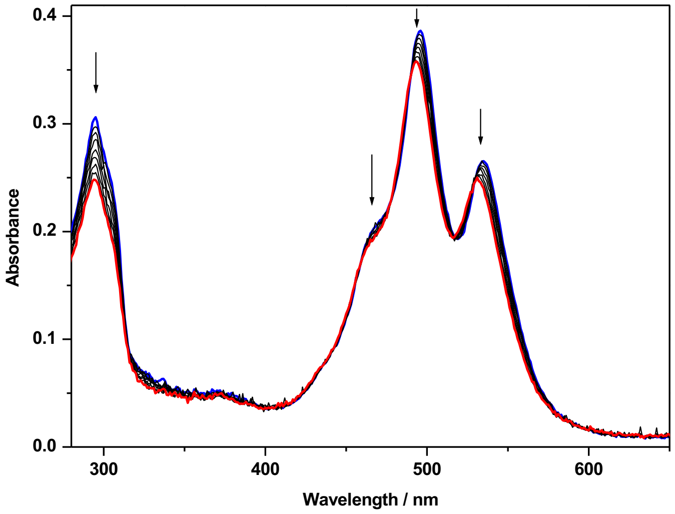

Figure S39. UV/vis spectra of **4** in DMSO at 295 (blue line), 303, 313, 323, 333, 343, 353, 363 and 368 (red line) K. Arrows indicate the effect of increasing temperature. The change was reversed on cooling. There is a general decrease in absorbance and a small blue shift on warming.

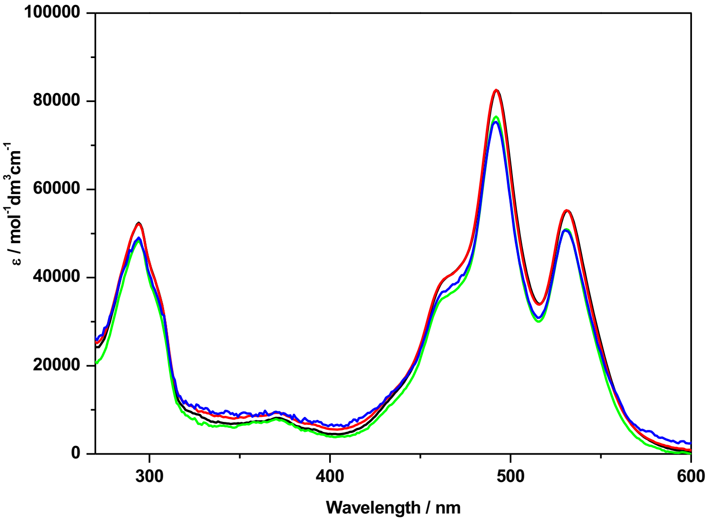

Figure S40. UV/vis spectra of **4** in dichloromethane at concentrations of 1.51 x 10^{-4} $moldm^{-3}$ (black line), 1.51 x 10^{-5} $moldm^{-3}$ (red line), 1.51 x 10^{-6} $moldm^{-3}$ (green line) and 1.51 x 10^{-7} $moldm^{-3}$ (blue line) using 0.05, 0.5, 2 and 10 cm pathlength cuvettes, respectively. Note that in order to maintain acceptable absorbance higher concentrations are not possible, even using a 0.05 cm pathlength cuvette.

EPR spectroscopy

The X-band EPR spectra of [**1**]$^-$ and [**6**]$^-$, generated by coulometric reduction of **1** and **6**, respectively, were recorded as fluid solutions at ambient temperature in CH_2Cl_2 containing 0.4 M [$^nBu_4N$][BF_4]. These spectra were very similar to each other and in each case the features were reasonably reproduced by simulation using couplings of the unpaired electron to three sets of four hydrogen atoms and two equivalent nitrogen atoms, although a slightly different linewidth was required for each simulation. The electrochemical reduction of **4** to [**4**]$^-$ also gave an EPR active species. The spectral width of [**4**]$^-$ (9.4 G) was ca. 72 % that of [**1**]$^-$ (13.0 G) and the spectrum was less well resolved. Nevertheless, the smaller spectral width and the observable features in the spectrum could be reasonably reproduced by simulation using three sets of eight hydrogen atoms and four equivalent nitrogen atoms, each at a magnitude half that required in the simulation of [**1**]$^-$, although a significantly larger linewidth was required in the simulation of [**4**]$^-$. This implies that the unpaired electron is either delocalised or is hopping over both PDI sites. For complete EPR line narrowing to occur, hence the unpaired electron to be fully shared between the two PDIs, then the charge hopping rate must exceed *ca*. 10^7 s^{-1} and this would result in a spectral width reduction to $1/\sqrt{2}$ (70 %) for a two centred system relative to the localised situation.[S14-S16] The line narrowing observed in the spectrum of [**4**]$^-$ is broadly consistent with this. Hence, on the EPR timescale the unpaired electron in [**4**]$^-$ appears to demonstrate properties of full delocalisation (or of fast hopping) between the two PDI centres.

The electrochemical reduction of [**4**]$^-$ to [**4**]$^{2-}$ resulted in the loss of solution EPR activity (double integration of the spectrum of [**4**]$^-$ versus that of [**4**]$^{2-}$ gave an 8 % residual signal). The loss of EPR activity for [**4**]$^{2-}$ excludes as a possibility the formation of a biradical containing two non-interacting electron spins i.e. two non-interacting doublet states. Furthermore, cooling the solution to 77 K failed to provide evidence for a ground state triplet. In the absence of a ground state triplet then loss of EPR activity in [**4**]$^{2-}$ suggests the presence of a ground state singlet. A singlet ground state must be derived from strong electron spin coupling consistent with the electrons entering an orbital in a shared π-based molecular framework. This conclusion appears consistent with results from UV/vis spectroscopy for [**4**]$^{2-}$ in which the electrons are considered to occupy a molecular based orbital manifold. Given that two-electron reduction of monomeric PDIs results in loss of EPR activity we did not investigate the [**4**]$^{4-}$ state by EPR spectroscopy.

The X-band EPR spectra of [**7**]$^-$, generated by coulometric reduction of **7**, was recorded as a fluid solution at ambient temperature in CH_2Cl_2 containing 0.4 M [$^nBu_4N$][BF_4] to act as a reference for an NDI based reduction in [**5**], with the spectrum of [**1**]$^-$, discussed above, acting as reference for a PDI based reduction. The EPR spectra of [**7**]$^-$ and [**1**]$^-$ are distinctly different. The experimental spectrum of [**7**]$^-$ was reasonable reproduced by simulation using couplings of the unpaired electron to two sets of four hydrogen atoms and two equivalent nitrogen atoms.

Coulometric reduction of **5**, at potential B (Figure 3b, main manuscript), gave an EPR active species. From the shape and position of the spectrum the electron appeared to be predominately occupied in a PDI based orbital. Comparison of the spectral width of [**5**]$^-$ with that of [**1**]$^-$ showed a small narrowing and the features in the experimental spectrum of [**5**]$^-$ were reasonably reproduced by simulation using couplings to the same set of nuclei as for [**1**]$^-$ but with the magnitude of these couplings scaled to 96 % compared to those for [**1**]$^-$. This suggests that the unpaired electron in [**5**]$^-$ is localised in the π-based

orbital of PDI for the majority of the time, although a small degree of delocalisation (or hopping) to NDI may also be apparent on this timescale.

a b

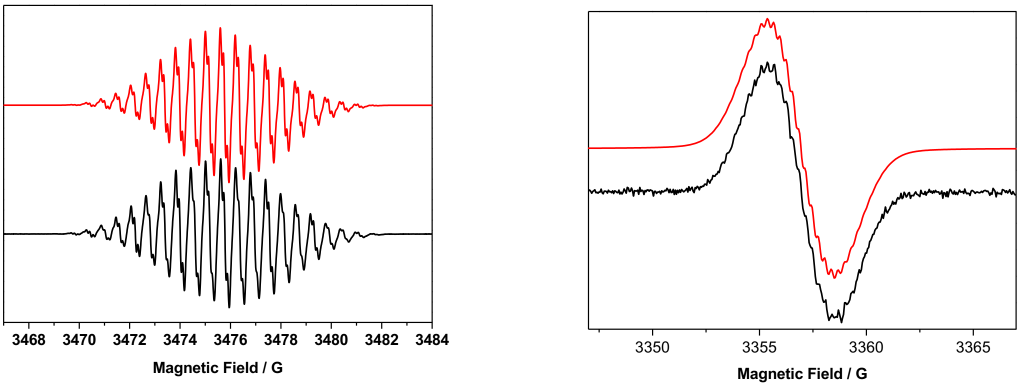

Figure S41. Experimental (black) and simulated (red) X-band EPR spectra of: (a) [**1**]$^-$; (b) [**4**]$^-$. Samples of the anions were electrogenerated by reduction of the parent molecules at 273 K as CH_2Cl_2 solutions containing [$^nBu_4N$][BF_4] (0.4 M) as the supporting electrolyte. Spectra were recorded at ambient temperature. Simulated spectra were generated using the parameters listed in Table S4.

The electrochemical reduction of [**5**]$^-$ to [**5**]$^{2-}$ resulted in reduced fluid solution EPR activity (double integration of the spectrum of [**5**]$^-$ versus that of [**5**]$^{2-}$ gave a 15 % residual signal). Cooling the solution to 77 K failed to provide evidence for a ground state triplet, although a signal consistent with the residual fluid solution spectrum was observed. We suggest that these data indicate the majority presence of a ground state singlet for [**5**]$^{2-}$ derived from strong electron spin coupling and consistent with the electrons entering an orbital in a shared π-based molecular framework. This conclusion appears consistent with results from UV/vis spectroscopy for [**5**]$^{2-}$. EPR activity is returned upon reduction to [**5**]$^{3-}$ whereby a spectral profile consistent with the presence of an NDI monoanion is observed. The spectral width and parameters used in the simulation of [**5**]$^{3-}$ indicate that the unpaired electron now occupies a localised NDI based orbital. This suggests that the third electron follows the trend noted in the UV/vis spectroelectrochemistry experiment and enters a PDI based orbital to form a spin paired, hence diamagnetic, PDI based dianion, thus the NDI derived electron, found in the ground state singlet of [**5**]$^{2-}$, is decoupled and this results in the observed NDI based EPR activity. Further reduction to [**5**]$^{4-}$ tends to reduce the intensity of bands associated with monoanionic NDI in the [**5**]$^{3-}$ spectrum consistent with the final electron entering an NDI based orbital to give two electrons in each of the π-centres, yielding [PDI]$^{2-}$-[NDI]$^{2-}$, as indicated by UV/vis spectroelectrochemistry (note that complete loss of the [NDI]$^-$ EPR spectrum was not achieved upon reduction to [**5**]$^{4-}$ but this may result from reoxidation of the sample to [**5**]$^{3-}$ during transfers of the tetra-anionic species).

Table S4. Parameters used in the simulation of experimental EPR spectroscopic data for compounds **1**, **4**-**7**.[a]

Compound	g_{iso}	a_{iso} / x 10^{-4} cm^{-1}	Linewidth / G	Lineshape
**[1]**$^-$	2.0034	1.670 (4H), 0.561 (4H), 0.122 (4H), 0.533 (2N)	0.10	Lorentzian
**[4]**$^-$	2.0034	0.835 (8H), 0.281 (8H), 0.061 (8H), 0.267 (4N)	0.30	Lorentzian
**[5]**$^-$	2.0034	1.603 (4H), 0.539 (4H), 0.117 (4H), 0.512 (2N)	0.53	Gaussian
**[5]**$^{3-}$	2.0038	1.768 (4H), 0.200 (4H), 0.910 (2N)	0.09	Lorentzian
**[6]**$^-$	2.0034	1.670 (4H), 0.561 (4H), 0.122 (4H), 0.533 (2N)	0.12	Lorentzian
**[7]**$^-$	2.0037	1.768 (4H), 0.200 (4H), 0.907 (2N)	0.09	Lorentzian

[a] In CH_2Cl_2 containing $[^nBu_4N][BF_4]$ (0.4 M) as supporting electrolyte, at ambient temperature.

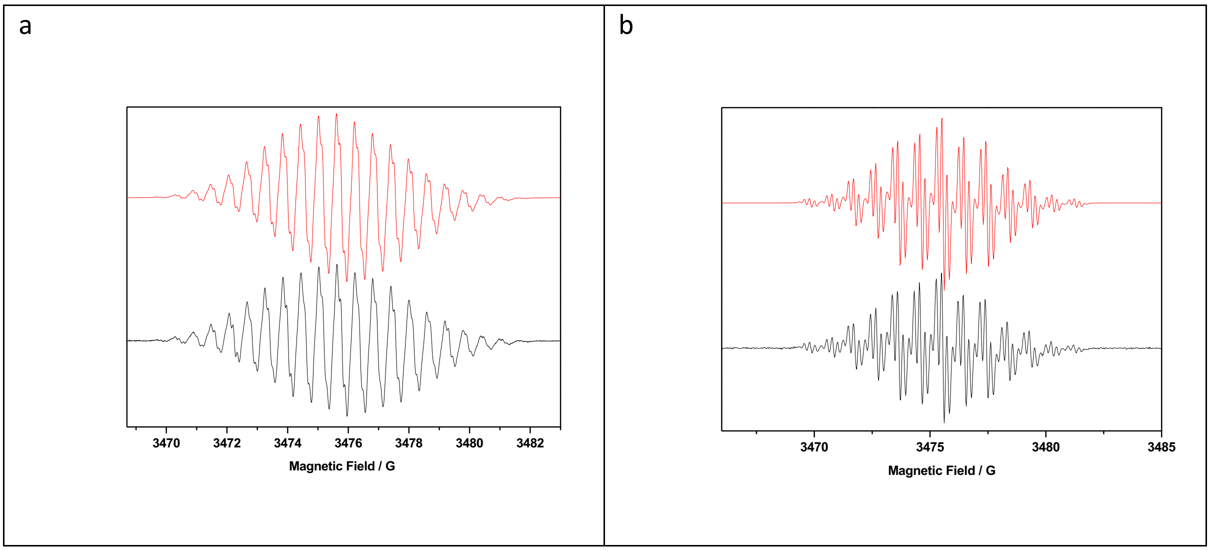

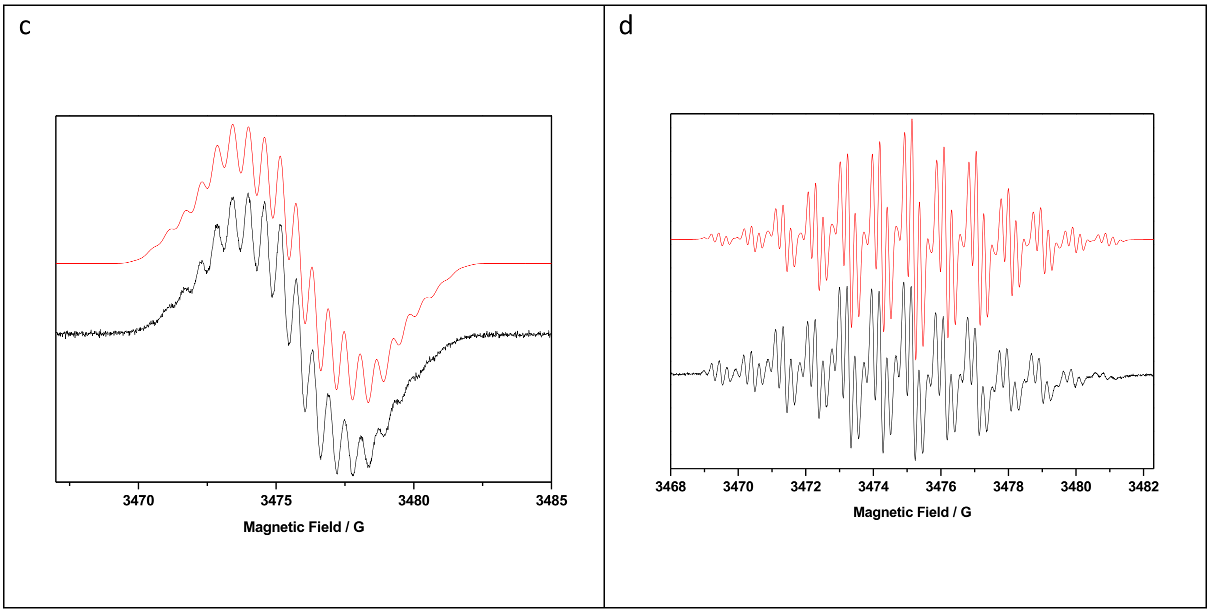

Figure S42. Experimental (black) and simulated (red) X-band EPR spectra of: (a) [**2**]$^{-}$; (b) [**7**]$^{-}$; (c) [**5**]$^{-}$; (d) [**5**]$^{3-}$. Samples of the anions were electrogenerated by reduction of the parent molecules at 273 K as CH_2Cl_2 solutions containing [$^nBu_4N$][BF_4] (0.4 M) as the supporting electrolyte. Spectra were recorded at ambient temperature. Simulated spectra were generated using the parameters listed in Table S4.

Molecular Dynamics Simulations of the Stability and Dynamic Behaviour

Methodology

The well-established force field, OPLS,[S17-S19] optimised for liquid simulations, was employed with the additional parameters derived from DFT calculations. Most of the OPLS parameters for the components of the molecule are tabulated in the general OPLS database, however for the PTCDI aromatic core and for the imidazolium chain, some parameters are not readily available. For the aromatic core, these parameters were taken from the Lennard-Jones potential and bonding terms (bonds, dihedral angles and improper torsions) describing uracil. Parameters for the charged chain are based on 1-ethyl-imidazole. The partial atomic charges were calculated using the restrained electrostatic potential (RESP) scheme[S20] based on DFT as implemented in the CP2K program package.[S21] Identical types of atoms were constrained to have the same charge. DFT calculations were performed at the gradient-corrected level by applying the BLYP[S22,S23] exchange-correlation functional within the GPW approach.[S24,S25] The electronic states were expanded by a double-ζ plus polarization basis set, DZVP,[S26] with norm-conserving pseudo-potentials for the description of core levels[S27-S29] and a plane-wave representation of the charge density with a cut-off of 500 Ry. The Martyna-Tuckerman Poisson solver[S30] was used in combination with a relatively large simulation box (up to 3.0 nm in the main dimension) to avoid computational artefacts. Chloroform molecules were described as a C-H united atom particle.

Due to the size limitations, only the molecular parts with unknown charges were considered in the RESP calculations to estimate the partial atomic charges, as shown in Figure S30. The position of cuts was carefully selected not to disrupt the electron delocalisation, *i.e.* in the proximity of single bonds. The charges on atoms near the cut, namely on CH_3 groups, were restricted to their standard OPLS values. The imidazolium chain was calculated in its charged (+1) state without the iodide counter-

anion. Molecules were initially optimised with the tight convergence criteria. The structures were built using VMD[S31] and Moltemplate[S32] visualisation packages.

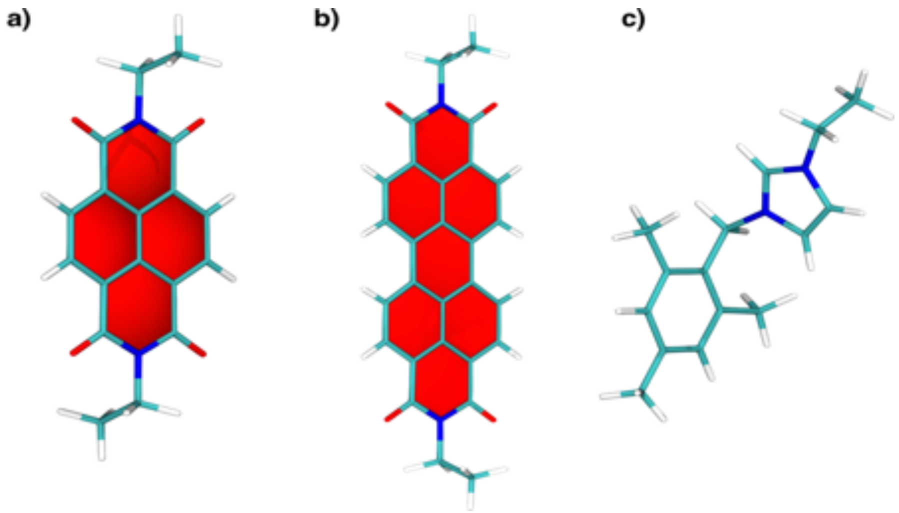

Figure S43. Models used for the RESP calculations.

Long-range electrostatic interactions were treated by the particle-particle particle-mesh method[S33] with the relative error in the electrostatic force of 10^{-5}. A cut-off distance of 1.2 nm was used for both Coulomb and van der Waals interactions. MD simulations were performed with a time step of 1 fs. The handcuff molecule was propagated in a NVE ensemble in a bath of chloroform molecules propagated as rigid bodies[S34] in a NPT ensemble (T=300k and P=1atm). Time constants for the thermostat and barostat were set to 0.1ps and 1ps, respectively.

The stability and dynamic behaviour of compound **5** suspended in chloroform solution was explored using classical molecular dynamics simulations as implemented in the LAMMPS simulation package[S17] and as shown in Figure S34.

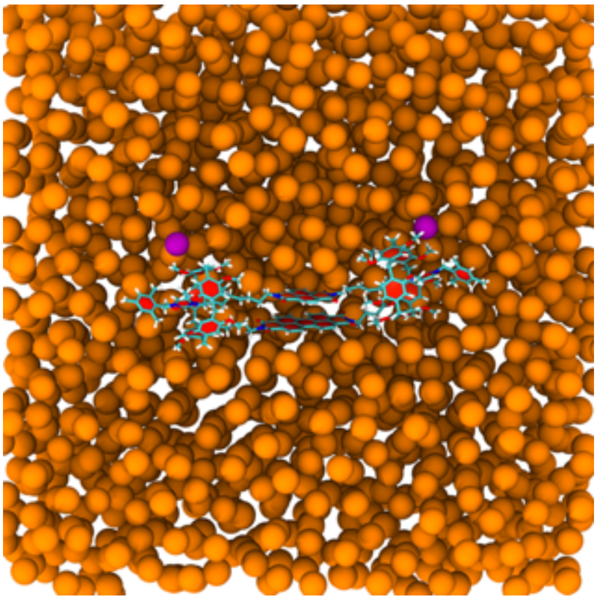

Figure S44. Simulation model: compound **5** surrounded by $CHCl_3$ molecules depicted, for clarity, as a single orange bead; iodine anions are shown in purple.

To discriminate between possible stable isomer structures, three different starting configurations were created as shown in Figures S32a-c. After 20 ns of equilibration and 180 ns of simulation time, the structure of isomer **5a** remains practically the same whereas isomers **5b** and **5c** exhibit some strong structural distortions (final structures are shown in Figures S32d-f). This indicates that isomer **5a** is the most stable conformation, which was further confirmed by the calculation of the interaction energy between two monomers of **5** (shown in red and blue), excluding the solvation energy. The interaction energy has been sampled every 10 ps and averaged over 25 ns, and the resulting values are predicted to be -134.4 kcal/mol for isomer **5a**, -125.3 kcal/mol for isomer **5b**, and -122.2 kcal/mol for isomer **5c**.

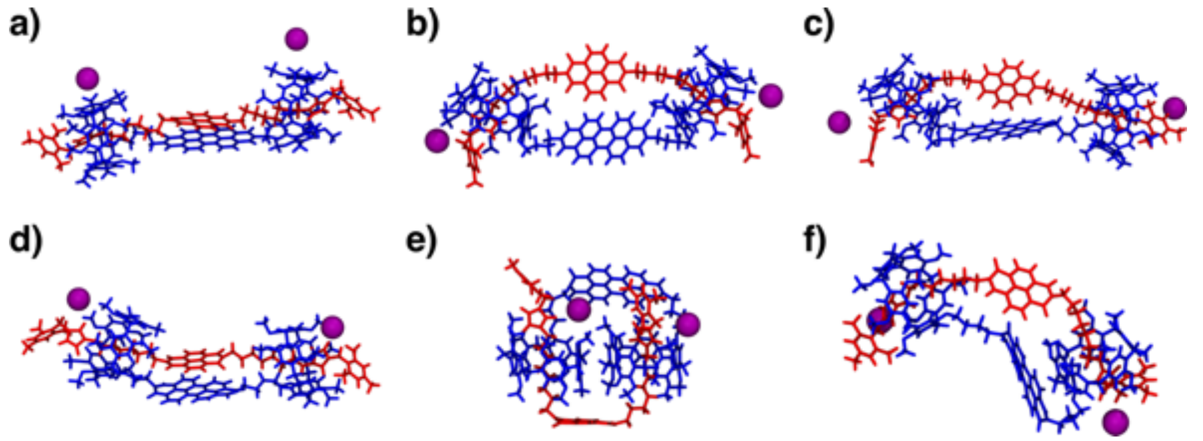

Figure S45. a-c: three starting configurations of **5** used in molecular dynamics simulations; d-f: snapshots taken after 200 ns of simulation time. Iodine anions are shown in purple, and the surrounding chloroform molecules are removed for clarity.

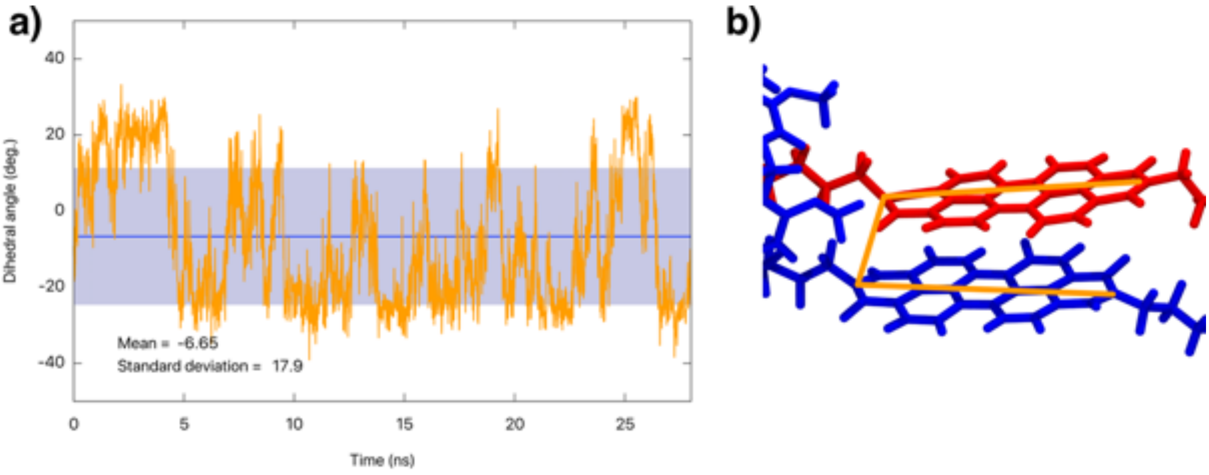

Figure S46. Calculated variation in the dihedral angle adopted between the principal axes of the two PDIs (shown in **b**) in **4**.

Details of DFT Calculations

Transition energies and oscillator strengths were computed with time-dependent density functional theory (TDDFT) with the CAM-B3LYP exchange-correlation functional[S35] and 6-311+G* basis set[S36] for the neutral, mono-reduced and di-reduced PDI and NDI species. Calculations were performed for the single molecule with a polarized continuum model (PCM)[S37-S39] with a dielectric constant of 78.39 to describe solvent and focus on the low energy region of the spectrum. Vibrational resolved spectra have been computed using the FCCLASSES code[S40] including Franck-Condon effects. The spectra were simulated at 100K, but we find the computed spectra to be relatively insensitive to the temperature. Harmonic vibrational frequencies have been computed using the B3LYP functional[S41] and 6-31G* basis set. The vibrational frequencies for the excited states were determined using the maximum overlap method.[S42] The spectra for PDI-PDI and PDI-NDI dimers were computed using TDDFT with the CAM-B3LYP functional and 6-31+G* basis set with the PCM solvent model. The structure for the PDI-PDI dimer was adapted from the molecular dynamics simulation, while a model PDI-NDI dimer was used with the two molecules placed 6 Å apart. Computed spectra were generated by convoluting Gaussian functions with a full-width at half maximum of 0.1 eV.

Computed Spectra for PDI and NDI monomers

Spectra were computed for monomer PDI and NDI species to serve as comparisons for further calculations. The computed transition energies are shown in Table S5 and computed spectra shown in Figure S40. The figures illustrate the electronic transitions associated with the observed bands in the computed spectra for the monomers. For the neutral species one intense band is observed which arises from a transition from the highest occupied molecular orbital (HOMO) to lowest unoccupied molecular orbital (LUMO). These transitions lie at 493 nm and 343 nm in PDI and NDI, respectively. For the mono-reduced species bands are observed at longer wavelength. These transitions arise from the excitation of the unpaired electron to higher lying unoccupied orbitals. For NDI two of these bands are observed at 682 and 519 nm, while for PDI only the lower energy of these bands can be distinguished clearly. The most intense transition is analogous to the HOMO to LUMO transition in the neutral species, although the band is shifted to lower energy. The lowest energy state for the di-reduced species is found to be a singlet state. For PDI the spectrum has one intense band at 568 nm that is associated with the (new) HOMO to LUMO transition.

Table S5. Computed transition energies (in nm) for PDI and NDI. Oscillator strengths are given in parenthesis. Only transitions with non-zero oscillator strength are shown.

Charge State	PDI	NDI
0	493 (1.01)	343 (0.48)
– 1	819 (0.37)	682 (0.08)
	690 (0.08)	519 (0.22)
	658 (0.71)	434 (0.34)
– 2	568 (1.76)	575 (0.23)
	561 (0.29)	431 (0.81)

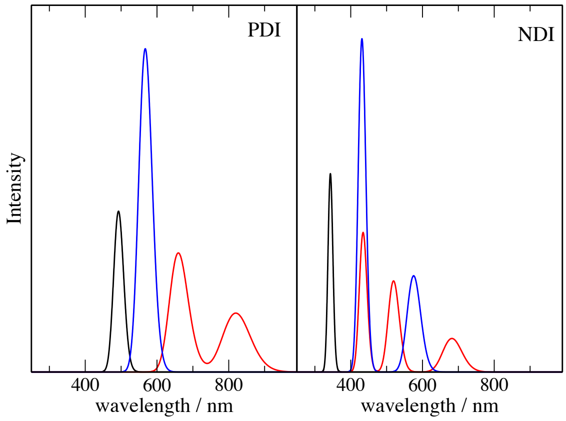

Figure S47. Computed spectra for PDI and NDI. Black line: neutral, red line: mono-reduced and blue line: di-reduced.

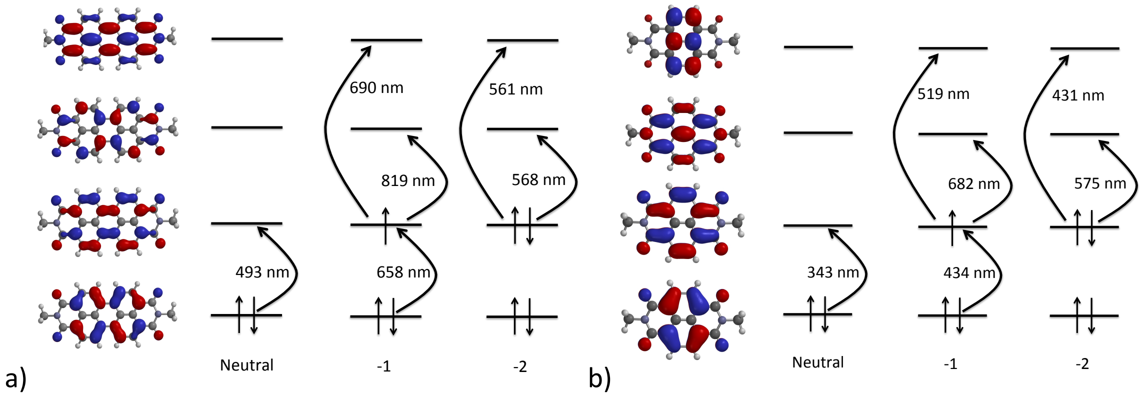

Figure S48. Electronic transitions associated with the observed bands a) in PDI and b) NDI.

Computed Vibronic Spectra: A feature of the experimental measurements is the spectral bands have a considerable degree of structure. For PDI we confirm that this structure is vibrational in origin. The excellent agreement between the computed spectra (shown below) and experiment confirms that the structure present in experiment can be attributed to vibrational structure.

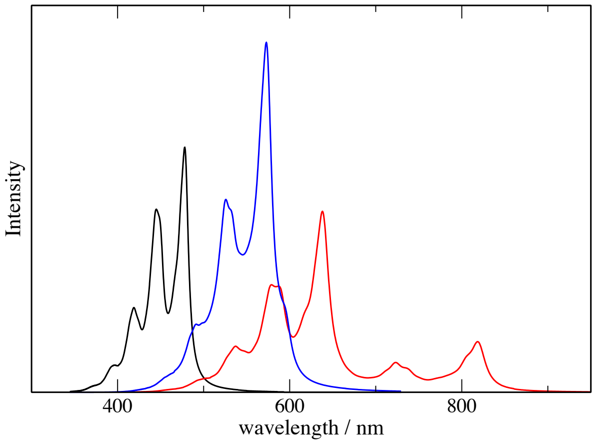

Figure S49. Computed vibrationally resolved spectra for PDI. Black line: neutral, red line: mono-reduced and blue line: di-reduced.

Compound 4, PDI-PDI Dimer

For all charge states, the calculated spectra are consistent with experiment. For the neutral PDI—PDI dimer, **4**, the calculations predict a single band a 529 nm. This arises from the HOMO to LUMO transition that can be localized on either PDI molecule. For the mono-reduced species, $[\mathbf{4}]^{-}$, the calculations show that the lower energy bands at 795 nm and 651 nm arise are associated with the PDI molecule that has been reduced while the band at 502 nm corresponds to the HOMO to LUMO on the neutral PDI molecule. In the di-reduced species $[\mathbf{4}]^{2-}$ a number of states are possible. Both electrons could localize on the same PDI molecule to form a closed-shell singlet state. The calculated spectrum for this state shows a single band at 490 nm, and does not agree with the measured spectrum. Alternatively, one electron can be localized on each PDI molecule. The resulting state can have either singlet or triplet spin. The spectra for these states are very similar and the computed spectrum for the triplet state is shown. This spectrum for this state gives a much closer agreement with experiment and confirms that in the di-reduced species each PDI molecule has a negative charge (additional electron). The band at longer wavelength arises from excitation of electrons in lower energy orbitals to the singly occupied orbital, while the band at 612 nm corresponds to excitation of the unpaired electron. We note that this band is shifted in energy compared with the mono-reduced species. For the quadruply reduced species, $[\mathbf{4}]^{4-}$, where two electrons have been added to each PDI molecule, the calculations predict an intense band at 494 nm with a much weaker band at 526 nm. These bands arise from transitions from the HOMO to the first and second unoccupied orbitals of the PDI molecules.

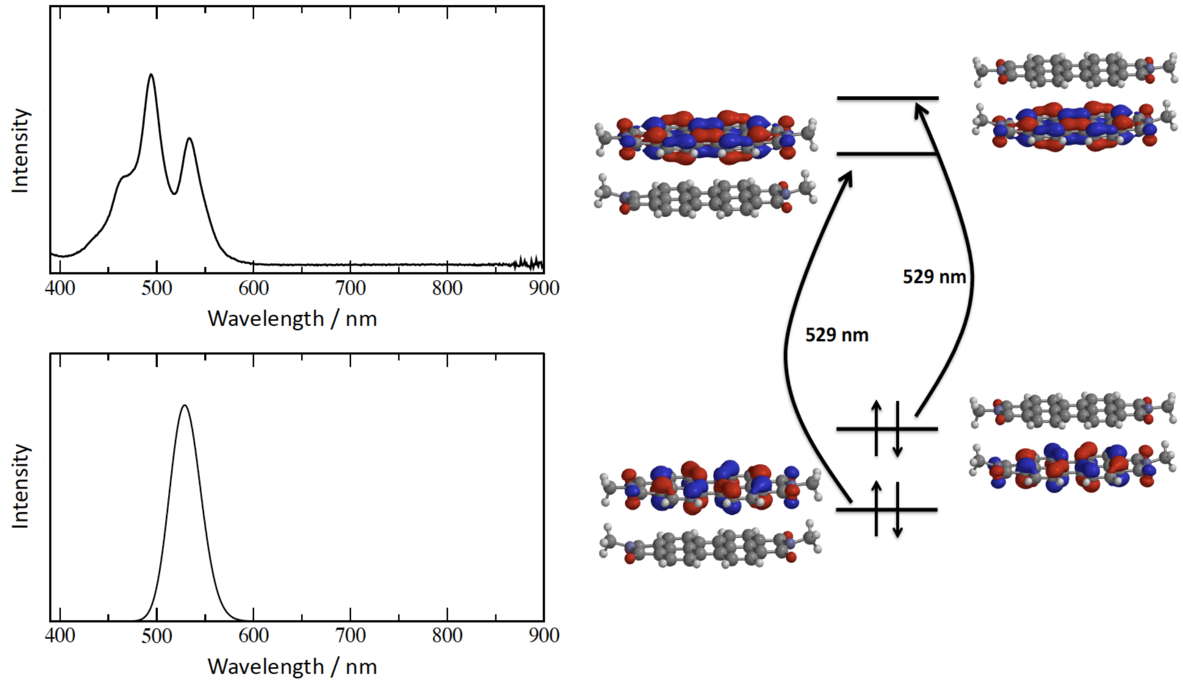

Figure S50. Experimental (upper panel) and computed (lower panel) spectra for [**4**] with the orbitals associated with the electronic transitions.

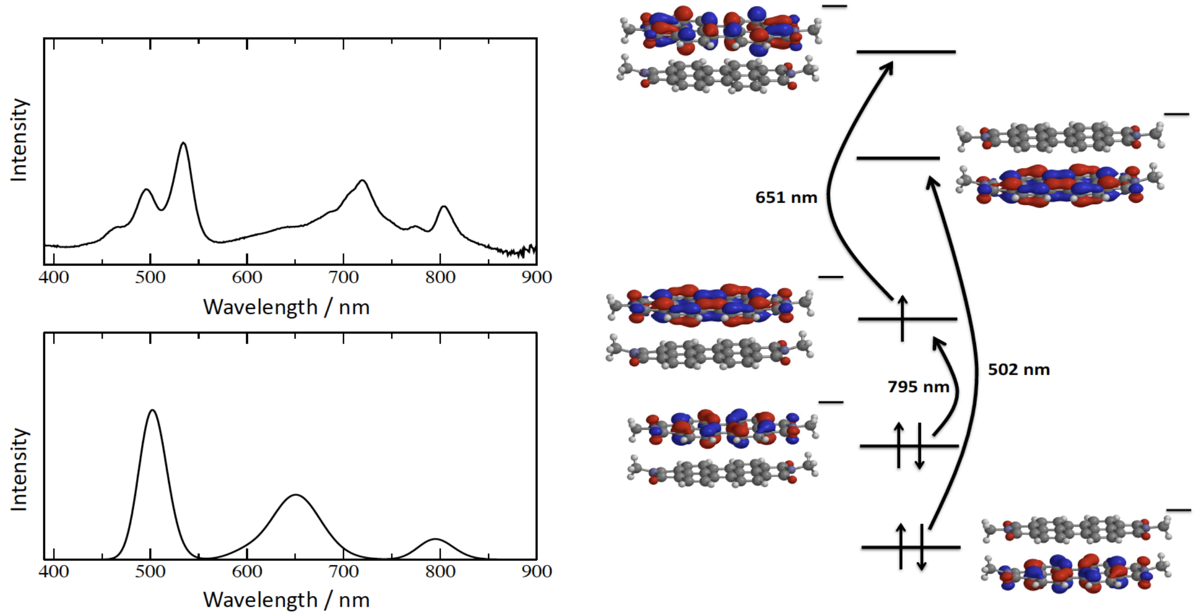

Figure S51. Experimental (upper panel) and computed (lower panel) spectra for mono-reduced, [**4**]$^{-}$, PDI^{-}-PDI dimer, with the orbitals associated with the electronic transitions. The negative charge is localized on the upper PDI molecule.

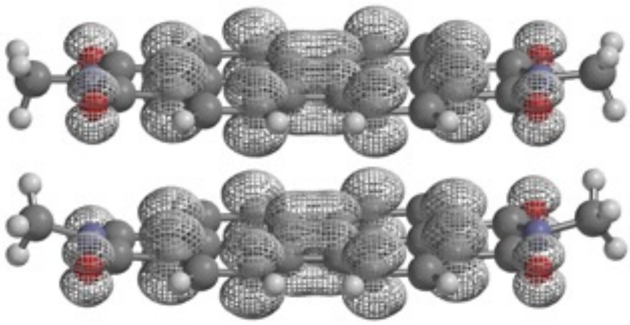

Figure S52. The HOMO of [**4**]$^{2-}$ exhibits spin density spread across both PDI species.

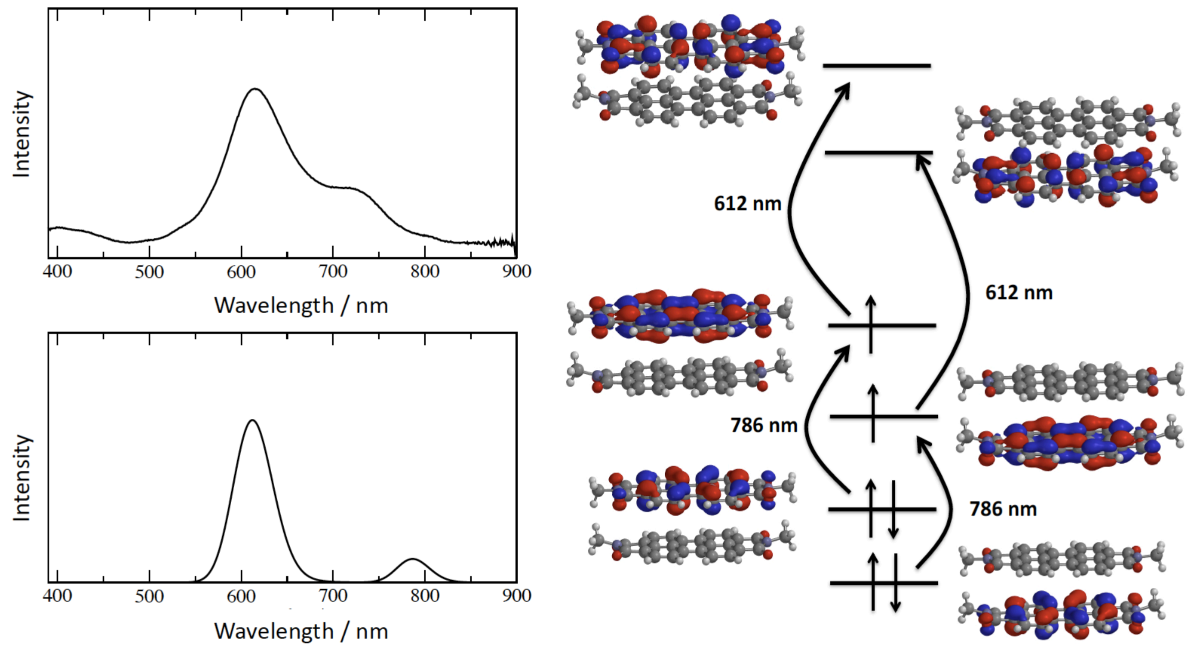

Figure S53. Experimental (upper panel) and computed (lower panel) spectra for di-reduced, [**4**]$^{2-}$, PDI^{-} -PDI^{-} dimer, with the orbitals associated with the electronic transitions.

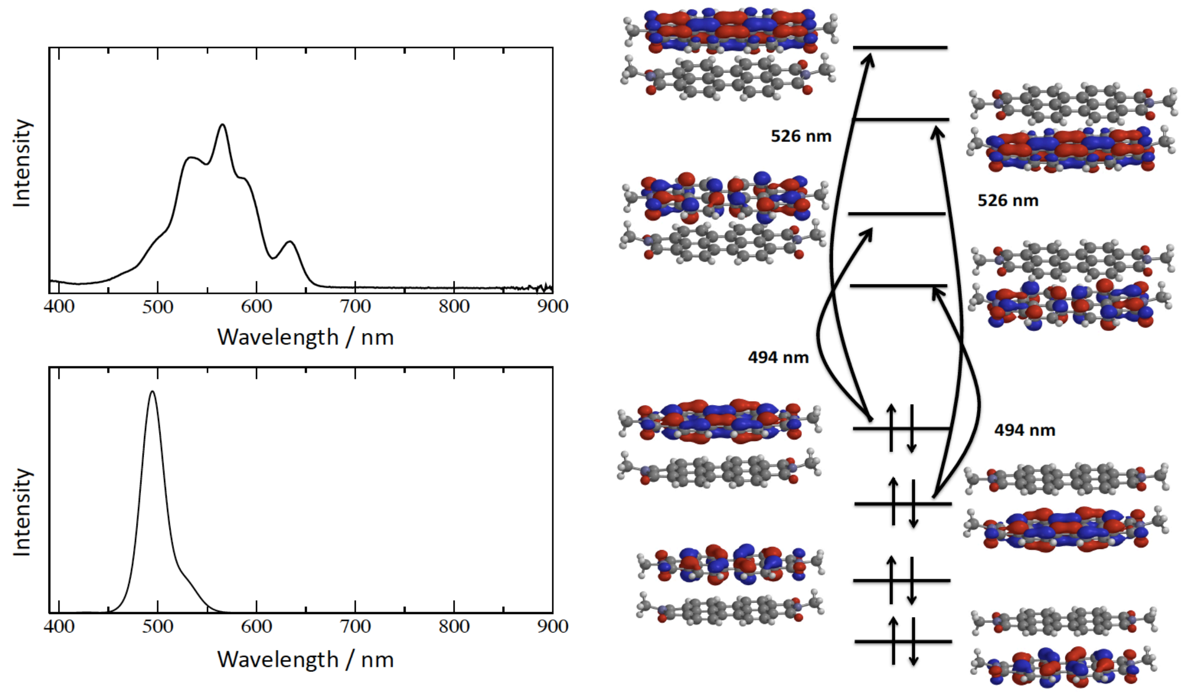

Figure S54. Experimental (upper panel) and computed (lower panel) spectra for [**4**]$^{4-}$, PDI^{2-} -PDI^{2-} dimer, with the orbitals associated with the electronic transitions.

Compound 5, PDI-NDI Dimer

For the neutral species two bands are predicted which arise from the HOMO to LUMO transitions on PDI (492 nm) and NDI (343 nm). In the mono-reduced species the lowest energy electron configuration has the additional electron on PDI. The resulting spectrum has a band a 343 nm associated with neutral NDI and bands at higher wavelength arising from the mono-reduced PDI. The experimental spectrum also has a band about 500 nm which is characteristic of neutral PDI. The di-reduced species has an unpaired electron on each molecule, see discussion above for compound **4**. The computed spectrum shows the bands associated with the mono-reduced state of each molecule. The calculated spectrum for the PDI^{2-}-NDI^{2-} dimer has a band at 570 nm arising from the HOMO to LUMO transition on PDI and bands at 400 nm and 522 nm arising from transition from the HOMO of NDI.

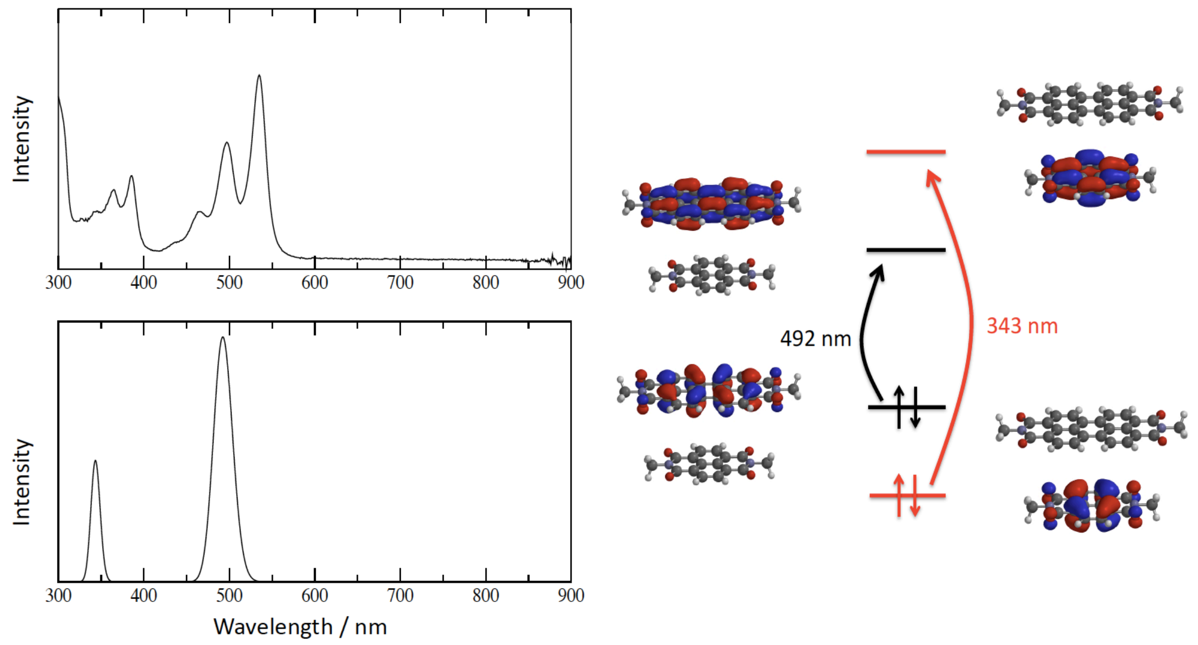

Figure S55. Experimental (upper panel) and computed (lower panel) spectra for the neutral PDI-NDI dimer, **5**, with the orbitals associated with the electronic transitions. Orbitals and transitions associated with NDI shown in red.

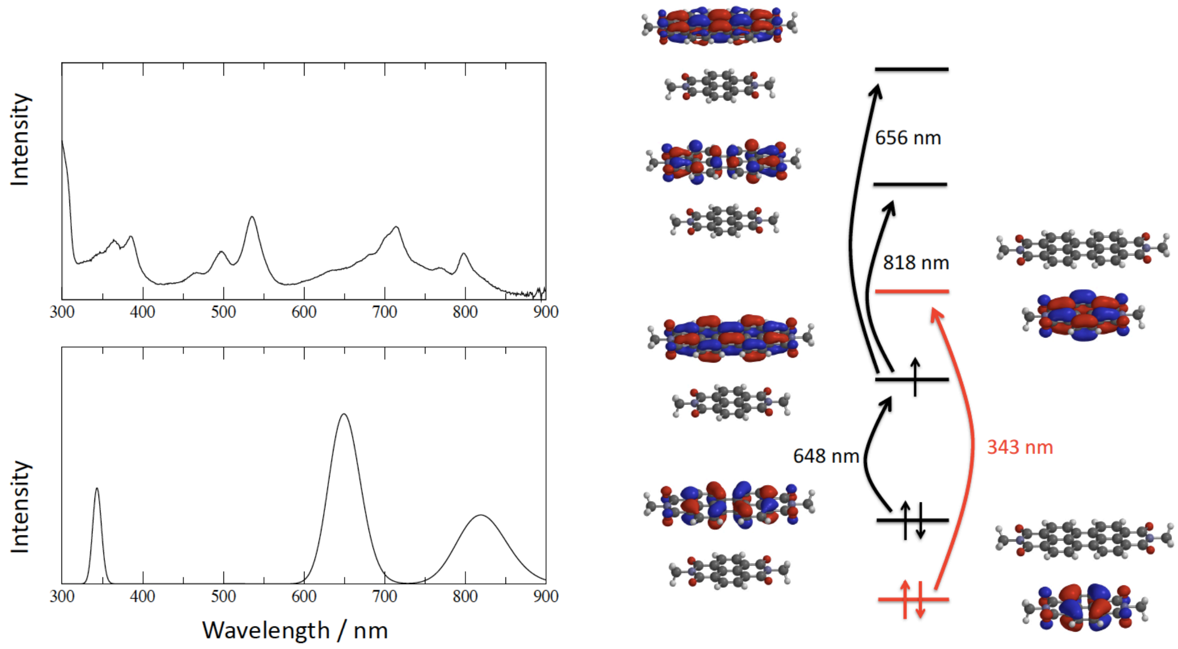

Figure S56. Experimental (upper panel) and computed (lower panel) spectra for the mono-reduced PDI$^-$-NDI dimer, [**5**]$^-$, with the orbitals associated with the electronic transitions. Orbitals and transitions associated with NDI shown in red.

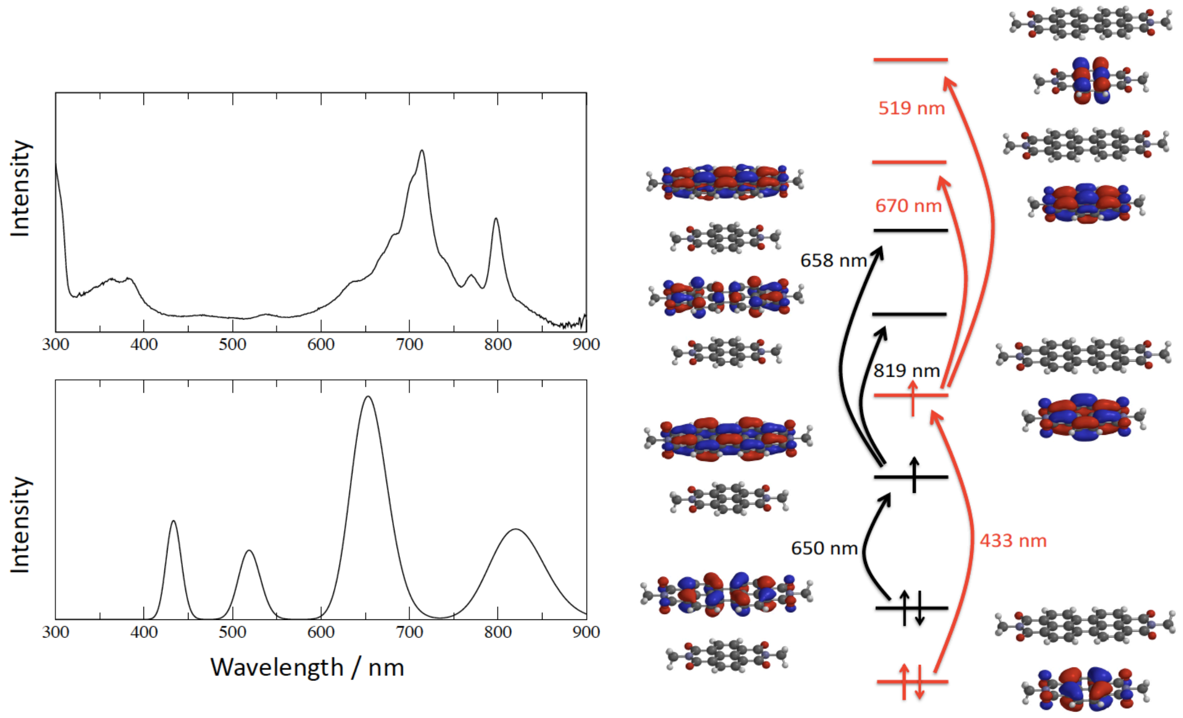

Figure S57. Experimental (upper panel) and computed (lower panel) spectra for the di-reduced PDI$^-$-NDI$^-$ dimer, [**5**]$^{2-}$, with the orbitals associated with the electronic transitions. Orbitals and transitions associated with NDI shown in red.

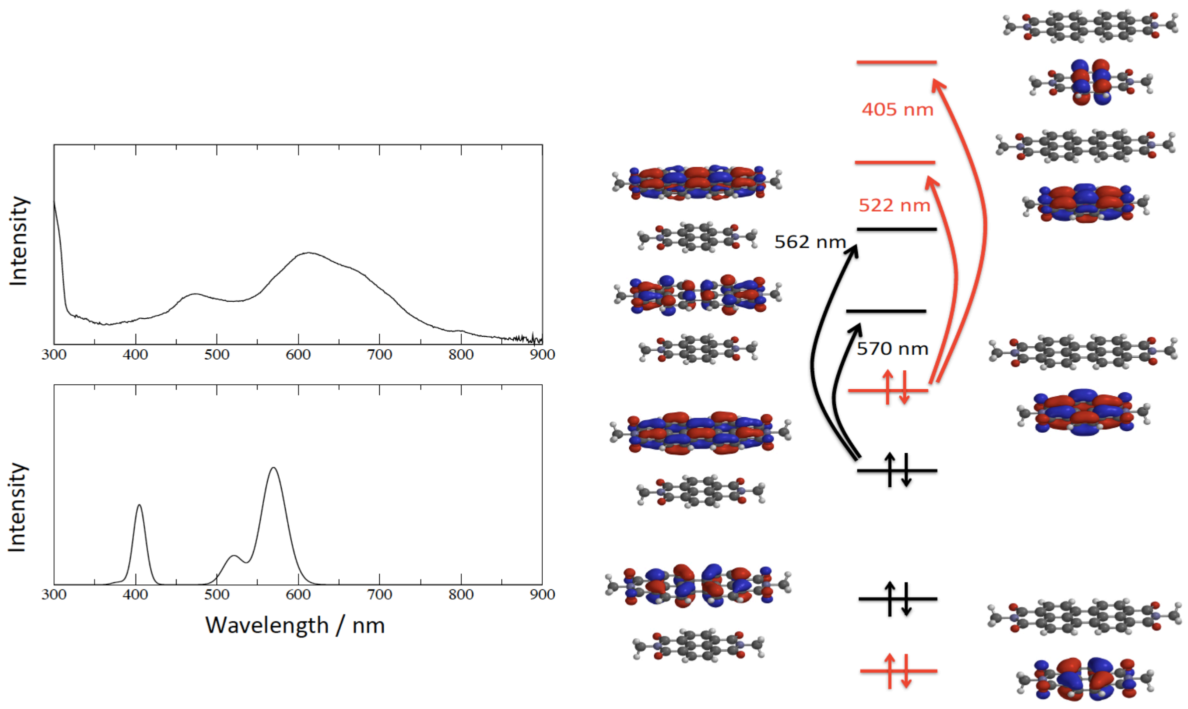

Figure S58. Experimental (upper panel) and computed (lower panel) spectra for the di-reduced PDI^{2-}-NDI^{2-} dimer, [**5**]$^{4-}$, with the orbitals associated with the electronic transitions. Orbitals and transitions associated with NDI shown in red.

References

S1. M. Ni, X.-Y. Hu, J. Jiang and L. Wang, *Chem. Commun.*, 2014, **50**, 1317-1319.

S2. Y.H.; Le Nguyen, J.R.; Winkler and H.B. Gray, *J. Phys. Chem. B*, 2007, **111**, 6628-6633.

S3. B.P. Bandgar, V.S. Sadavarte and L.S. Uppalla, *Tetrahedron Lett.,* 2001, **42**, 951-953.

S4. J.Kubas, *Inorg. Synth.*, 1990, **28**, 68.

S5. A. M. Brouwer, *Pure Appl. Chem*., 2011, **83**, 2213-2228.

S6. I. Noviandri, K.N. Brown, D.S. Fleming, P.T. Gulyas, P.A. Lay, A.F. Masters and L. Phillips, *J. Phys. Chem. B*, 1999, **103**, 6713-6722.

S7. V. Ganesan, S.V. Rosokha and J.K. Kochi, *J. Am. Chem. Soc.*, 2003, **125**, 2559-2571.

S8. C.-C. You and F. Würthner, *J. Am. Chem. Soc*., 2003, **125**, 9716-9725.

S9. J.-M. Lü, S. V. Rosokha and J. K. Kochi, *J. Am. Chem. Soc*., 2003, **125**, 12161-12171.

S10. C. Zhao, Y. Zhang, R. Li, X. Li and J. Jiang, *J. Org. Chem.*, 2007, **72**, 2402-2410.

S11. B. Liang, Y. Zhang, Y. Wang, W. Xu and X.Li, *J. Mol. Struct.*, 2009, **917**, 133-141.

S12. A.G. Slater (neé Phillips), E.S. Davies, S.P. Argent, W. Lewis, A.J. Blake, J. McMaster and N.R. Champness, *J. Org. Chem.*, 2013, **78**, 2853-2862.

S13. C. M. Mauck, R. M. Young and M. R. Wasielewski, J. Phys. Chem. A, 2017, **121**, 784-792.

S14. J. R. Norris, R. A. Uphaus, H. L. Crespi and J. Katz, *Proc. Natl. Acad. Sci. USA*, 1971, **68**, 625-628.

S15. Y. Wu, M. Frasconi, D.M. Gardner, P.R. McGonigal, S.T. Schneebeli, M.R., Wasielewski and J.F. Stoddart, *Angew. Chem. Int. Ed.*, 2014, **53**, 9476-9481.

S16. T. H. Wilson, T. A. Zeidan, M. Hariharan, F. D. Lewis and M. R. Wasielewski, *Angew. Chem. Int. Ed.*, 2010, **49**, 2385-2388.

S17. S. Plimpton, *J. Comput. Phys*. 1995, **117**, 1–19.

S18. W.L. Jorgensen and J.Tirado-Rives, *J. Am. Chem. Soc*. 1988, **110**, 1657–1666.

S19. W.L. Jorgensen, D. S. Maxwell and J. Tirado-Rives, *J. Am. Chem. Soc*. 1996, **118**, 11225–11236.

S20. C.C.I. Bayly, P. Cieplak, W.D. Cornell and P.A. Kollman, *J. Phys. Chem*. 1993, **97**, 10269–10280.

S21. J. Hutter, M. Iannuzzi, F. Schiffmann and J. VandeVondele, *WIREs Comput. Mol. Sci*. 2014, **4**, 15-25.

S22. A.D. Becke, *Phys. Rev. A* 1988, **38**, 3098–3100.

S23. C. Lee, W. Yang and R.G. Parr, *Phys. Rev. B* 1988, **37**, 785–789.

S24. G. Lippert, J. Hutter and M. Parrinello, *Mol. Phys*. 1997, **92**, 477–488.

S25. J. Vandevondele, M. Krack, F. Mohamed, M. Parrinello, T. Chassaing and J. Hutter, *Comput. Phys. Commun*. 2005, **167**, 103–128.

S26. J. VandeVondele and J.Hutter, *J. Chem. Phys*. 2007, **127**, 114105.

S27. S. Goedecker, M. Teter and J. Hutter, *Phys. Rev. B* 1996, **54**, 1703–1710.

S28. C. Hartwigsen, S. Goedecker and J. Hutter, *Phys. Rev. B* 1998, **58**, 3641–3662.

S29. M. Krack, *Theor. Chem. Acc*. 2005, **114**, 145–152.

S30. G.J. Martyna and M.E. Tuckerman, *J. Chem. Phys*. 1999, **110**, 2810–2821.

S31. W. Humphrey, A. Dalke and K. Schulten, *J. Mol. Graph*. 1996, **14**, 33–38.

S32. A.I. Jewett, Z. Zhuang and J-E. Shea, *Biophys. J*. 2013, **104**, 169.

S33. R.W. Hockney and J. W. Eastwood, Computer simulation using particles. (Adam Hilger, 1989).

S34. G.J. Martyna, D. J. Tobias and M.L. Klein, *J. Chem. Phys*. 1994, **101**, 4177.

S35. T. Yanai, D. Tew and N. Handy, *Chem. Phys. Lett*. 2004, **393**, 51-57.

S36. R. Krishnan, J.S. Binkley, R. Seeger and J.A. Pople, *J. Chem. Phys*. 1980, **72**, 650.

S37. T. N. Truong and E. V. Stefanovich, *Chem. Phys. Lett*. 1995, **240**, 253-260.

S38. V.; Barone and M. Cossi, *J. Phys. Chem. A* 1998, **102**, 1995-2001.

S39. M. Cossi, N. Rega, G. Scalmani and V. Barone, *J. Comput. Chem*. 2003, **24**, 669-681.

S40. F.Santoro, FCclasses: A Fortran 77 Code; 2008. (available via the Internet at http://www.pi.iccom.cnr.it/fcclasses)

S41. P. J. Stephens, F. J. Devlin, C. F. Chabolowski and M. J. Frisch, *J. Phys. Chem*. 1994, **98**, 11623-11627.

S42. A. T. B. Gilbert, N. A. Besley, P. M. W. Gill, *J. Phys. Chem. A* 2008, **112**, 13164-13171.
